# Antiviral efficacy of molnupiravir versus ritonavir-boosted nirmatrelvir in patients with early symptomatic COVID-19 (PLATCOV): an open-label, phase 2, randomised, controlled, adaptive trial

**DOI:** 10.1016/S1473-3099(23)00493-0

**Published:** 2023-09-28

**Authors:** William H K Schilling, Podjanee Jittamala, James A Watson, Simon Boyd, Viravarn Luvira, Tanaya Siripoon, Thundon Ngamprasertchai, Elizabeth M Batty, Cintia Cruz, James J Callery, Shivani Singh, Manisaree Saroj, Varaporn Kruabkontho, Thatsanun Ngernseng, Nuttakan Tanglakmankhong, Jaruwan Tubprasert, Mohammad Yazid Abdad, Wanassanan Madmanee, Jindarat Kouhathong, Kanokon Suwannasin, Watcharee Pagornrat, Nattaporn Piaraksa, Pongtorn Hanboonkunupakarn, Borimas Hanboonkunupakarn, Kittiyod Poovorawan, Manus Potaporn, Attasit Srisubat, Bootsakorn Loharjun, Walter R J Taylor, Vasin Chotivanich, Kesinee Chotivanich, Mallika Imwong, Sasithon Pukrittayakamee, Arjen M Dondorp, Nicholas P J Day, Mauro M Teixeira, Watcharapong Piyaphanee, Weerapong Phumratanaprapin, Nicholas J White

**Affiliations:** Mahidol Oxford Tropical Medicine Research Unit; Faculty of Tropical Medicine, Mahidol University, Bangkok, Thailand; Centre for Tropical Medicine and Global Health, Nuffield Department of Medicine, University of Oxford, Oxford, UK; Mahidol Oxford Tropical Medicine Research Unit; Department of Tropical Hygiene; Mahidol Oxford Tropical Medicine Research Unit; Faculty of Tropical Medicine, Mahidol University, Bangkok, Thailand; Centre for Tropical Medicine and Global Health, Nuffield Department of Medicine, University of Oxford, Oxford, UK; Mahidol Oxford Tropical Medicine Research Unit; Faculty of Tropical Medicine, Mahidol University, Bangkok, Thailand; Centre for Tropical Medicine and Global Health, Nuffield Department of Medicine, University of Oxford, Oxford, UK; Department of Clinical Tropical Medicine; Department of Clinical Tropical Medicine; Department of Clinical Tropical Medicine; Mahidol Oxford Tropical Medicine Research Unit; Faculty of Tropical Medicine, Mahidol University, Bangkok, Thailand; Centre for Tropical Medicine and Global Health, Nuffield Department of Medicine, University of Oxford, Oxford, UK; Mahidol Oxford Tropical Medicine Research Unit; Faculty of Tropical Medicine, Mahidol University, Bangkok, Thailand; Centre for Tropical Medicine and Global Health, Nuffield Department of Medicine, University of Oxford, Oxford, UK; Mahidol Oxford Tropical Medicine Research Unit; Faculty of Tropical Medicine, Mahidol University, Bangkok, Thailand; Centre for Tropical Medicine and Global Health, Nuffield Department of Medicine, University of Oxford, Oxford, UK; Mahidol Oxford Tropical Medicine Research Unit; Mahidol Oxford Tropical Medicine Research Unit; Mahidol Oxford Tropical Medicine Research Unit; Mahidol Oxford Tropical Medicine Research Unit; Mahidol Oxford Tropical Medicine Research Unit; Mahidol Oxford Tropical Medicine Research Unit; Mahidol Oxford Tropical Medicine Research Unit; Faculty of Tropical Medicine, Mahidol University, Bangkok, Thailand; Centre for Tropical Medicine and Global Health, Nuffield Department of Medicine, University of Oxford, Oxford, UK; Mahidol Oxford Tropical Medicine Research Unit; Mahidol Oxford Tropical Medicine Research Unit; Mahidol Oxford Tropical Medicine Research Unit; Mahidol Oxford Tropical Medicine Research Unit; Department of Clinical Tropical Medicine; Bangplee Hospital, Ministry of Public Health, Bangplee, Thailand; Mahidol Oxford Tropical Medicine Research Unit; Department of Clinical Tropical Medicine; Mahidol Oxford Tropical Medicine Research Unit; Department of Clinical Tropical Medicine; Department of Medical Services, Ministry of Public Health, Nonthaburi, Thailand; Department of Medical Services, Ministry of Public Health, Nonthaburi, Thailand; Department of Medical Services, Ministry of Public Health, Nonthaburi, Thailand; Mahidol Oxford Tropical Medicine Research Unit; Faculty of Tropical Medicine, Mahidol University, Bangkok, Thailand; Centre for Tropical Medicine and Global Health, Nuffield Department of Medicine, University of Oxford, Oxford, UK; Faculty of Medicine, Navamindradhiraj University, Bangkok, Thailand; Mahidol Oxford Tropical Medicine Research Unit; Department of Clinical Tropical Medicine; Mahidol Oxford Tropical Medicine Research Unit; Department of Molecular Tropical Medicine and Genetics; Mahidol Oxford Tropical Medicine Research Unit; Department of Clinical Tropical Medicine; Mahidol Oxford Tropical Medicine Research Unit; Faculty of Tropical Medicine, Mahidol University, Bangkok, Thailand; Centre for Tropical Medicine and Global Health, Nuffield Department of Medicine, University of Oxford, Oxford, UK; Mahidol Oxford Tropical Medicine Research Unit; Faculty of Tropical Medicine, Mahidol University, Bangkok, Thailand; Centre for Tropical Medicine and Global Health, Nuffield Department of Medicine, University of Oxford, Oxford, UK; Clinical Research Unit, Center for Advanced and Innovative Therapies, Universidade Federal de Minas Gerais, Belp Horizonte, Brazil; Department of Clinical Tropical Medicine; Department of Clinical Tropical Medicine; Mahidol Oxford Tropical Medicine Research Unit; Faculty of Tropical Medicine, Mahidol University, Bangkok, Thailand; Centre for Tropical Medicine and Global Health, Nuffield Department of Medicine, University of Oxford, Oxford, UK

## Abstract

**Background:**

Molnupiravir and ritonavir-boosted nirmatrelvir are the two leading oral COVID-19 antiviral treatments, but their antiviral activities in patients have not been compared directly. The aim of this ongoing platform trial is to compare different antiviral treatments using the rate of viral clearance as the measure of antiviral effect.

**Methods:**

PLATCOV is an open-label, multicentre, phase 2, randomised, controlled, adaptive pharmacometric platform trial running in Thailand, Brazil, Pakistan, and Laos. The component of the trial reported here was conducted in the Hospital for Tropical Diseases, Faculty of Tropical Medicine, Mahidol University, Bangkok, Thailand. We recruited low-risk adult patients aged 18–50 years with early symptomatic COVID-19 (<4 days of symptoms). Eligible patients were randomly assigned using block randomisation via a centralised web app to one of seven treatment groups: molnupiravir, ritonavir-boosted nirmatrelvir, casirivimab–imdevimab, tixagevimab–cilgavimab, favipiravir, fluoxetine, or no study drug. The no study drug group comprised a minimum proportion of 20% of patients at all times, with uniform randomisation ratios applied across the active treatment groups. Results for the concurrently randomised molnupiravir, ritonavir-boosted nirmatrelvir, and no study drug groups are reported here. The primary endpoint was the rate of oropharyngeal viral clearance assessed in a modified intention-to-treat population, defined as patients with more than 2 days of follow-up. Safety was assessed in all participants who took at least one dose of the medication. The viral clearance rate was derived under a Bayesian hierarchical linear model fitted to the log_10_ viral densities in standardised duplicate oropharyngeal swab eluates taken daily over 1 week (18 measurements). Treatment groups with a probability of more than 0·9 that viral clearance was accelerated by more than 20% compared with no drug entered a non-inferiority comparison (with a 10% non-inferiority margin) compared with the platform’s current most effective drug. This ongoing trial is registered at ClinicalTrials.gov, NCT05041907.

**Findings:**

Between June 6, 2022, and Feb 23, 2023, 209 patients in Thailand were enrolled and concurrently randomly assigned to molnupiravir (n=65), ritonavir-boosted nirmatrelvir (n=59), or no study drug (n=85). 129 (62%) of the patients were female and 80 (38%) were male. Relative to the no study drug group, the rates of viral clearance were 37% (95% credible interval 16–65) faster with molnupiravir and 84% (54–119) faster with ritonavir-boosted nirmatrelvir. In the non-inferiority comparison, viral clearance was 25% (10–38) slower with molnupiravir than ritonavir-boosted nirmatrelvir. Molnupiravir was removed from the study platform when it reached the prespecified inferiority margin of 10% compared with ritonavir-boosted nirmatrelvir. Median estimated viral clearance half-lives were 8·5 h (IQR 6·7–10·1) with ritonavir-boosted nirmatrelvir, 11·6 h (8·6–15·4) with molnupiravir, and 15o5 h (11·9–21·2) with no study drug. Viral rebound occurred more frequently following nirmatrelvir (six [10%] of 58) compared with the no study drug (one [1%] of 84; p=0·018) or the molnupiravir (one [2%] of 65; p=0·051) groups. Persistent infections following molnupiravir had more viral mutations (three of nine patients had an increased number of single nucleotide polymorphisms in samples collected at 7 or more days compared with those at baseline) than after nirmatrelvir (zero of three) or no study drug (zero of 19). There were no adverse events of grade 3 or worse, or serious adverse events in any of the reported treatment groups.

**Interpretation:**

Both molnupiravir and ritonavir-boosted nirmatrelvir accelerate oropharyngeal SARS-CoV-2 viral clearance in patients with COVID-19, but the antiviral effect of ritonavir-boosted nirmatrelvir was substantially greater. Measurement of oropharyngeal viral clearance rates provides a rapid and well tolerated approach to the assessment and comparison of antiviral drugs in patients with COVID-19. It should be evaluated in other acute viral respiratory infections.

**Funding:**

Wellcome Trust through the COVID-19 Therapeutics Accelerator.

## Introduction

Effective antiviral drugs and monoclonal antibodies accelerate viral clearance and prevent progression to severe disease and death in early COVID-19.^1,2^ Large randomised controlled trials have shown clinical efficacy with corresponding accelerated nasopharyngeal viral clearance for monoclonal antibodies, parenteral remdesivir, and new oral antiviral drugs molnupiravir and ritonavir-boosted nirmatrelvir.^1–5^ Molnupiravir and ritonavir-boosted nirmatrelvir have been used widely, although availability in low resource settings is still limited, particularly for nirmatrelvir.^6^ Both medications have disadvantages. For molnupiravir, there have been doubts over the drug’s efficacy. The initial reported clinical benefit, based on an interim analysis, was revised down when further data became available and, despite approvals, some countries opted to cancel their orders.^7^ An open-label randomised study of molnupiravir in the UK of 26 411 vaccinated patients at high risk in the community showed no decrease in subsequent COVID-19 hospitalisation or death, although there were improvements in time to recovery and viral clearance rates.^8^ An additional concern is the creation of mutant viruses; molnupiravir is the prodrug of N^4^-hydroxycytidine, which causes such a high frequency of SARS-CoV-2 mutations that replication is prevented (known as error catastrophe).^9^ Nirmatrelvir, a 3C-like (main) protease inhibitor is a potent antiviral in vitro, but it has been associated with viral and symptom rebounds,^10^ although there is uncertainty about whether viral rebound is more common with nirmatrelvir than other antivirals or no treatment.^11^ Clinically significant dysgeusia is common with ritonavir-boosted nirmatrelvir. Concomitant administration of ritonavir is required to boost nirmatrelvir levels, and thereby increase exposure. Ritonavir is contraindicated in many patients because of drug–drug interactions.

Although widely purchased by governments and promoted in the private health sector, molnupiravir and nirmatrelvir have not been compared directly or compared with other COVID-19 therapeutics. As a result, current use largely depends on availability, cost, estimates of effect from preregistration studies conducted earlier in the pandemic, and perceptions regarding potential drawbacks (ie, tolerability and viral rebound *vs* mutant creation).

Clinical outcomes depend on the clinical and immune status of the study population and the virulence of the virus. COVID-19 has become substantially milder, although severe infections still occur, particularly in groups at high risk. The increasing rarity of deterioration requiring hospitalisation and death mean that prohibitively large comparative studies are needed to detect clinically important differences. We therefore aimed to assess the in-vivo antiviral activities of molnupiravir and ritonavir-boosted nirmatrelvir in adults with early symptomatic COVID-19.

## Methods

### Study design

PLATCOV is an ongoing, open-label, multicentre, phase 2, randomised, controlled, adaptive pharma- cometric platform trial running in Thailand, Brazil, Pakistan, and Laos.^12,13^ The component of the trial reported here was conducted in the Hospital for Tropical Diseases, Faculty of Tropical Medicine, Mahidol University, Bangkok, Thailand. The trial provides a standardised quantitative comparative method for in-vivo assessment of potential antiviral treatments in adults at low risk with early symptomatic COVID-19. Potential antiviral treatments enter the randomised trial when they become available and leave when prespecified endpoints are reached.

All patients received standard symptomatic treatment. The initial drugs studied were ivermectin, favipiravir, remdesivir, and the casirivimab–imdevimab monoclonal antibody cocktail. All these groups have now stopped, having reached the prespecified endpoints for efficacy or lack of efficacy. Additional groups (molnupiravir, ritonavir-boosted nirmatrelvir, fluoxetine, and the tixagevimab–cilgavimab monoclonal antibody cocktail) were introduced subsequently. The analysis reported here includes only patients from Thailand concurrently allocated to molnupiravir, ritonavir-boosted nirmatrelvir, or no study drug because the test drugs were unavailable at the other study sites. During this period, patients were also randomly assigned to the casirivimab–imdevimab monoclonal antibody cocktail (until Oct 20, 2022),^14^ tixagevimab–cilgavimab (ongoing), favipiravir (until Oct 30, 2022),^15^ and fluoxetine (ongoing), the results for which will be reported elsewhere. The ivermectin and remdesivir treatment groups had already stopped (for lack of efficacy and for efficacy, respectively). The results are reported elsewhere.^12,14,16^

PLATCOV is coordinated and monitored by the Mahidol Oxford Tropical Medicine Research Unit (MORU) in Bangkok. The trial was overseen by a trial steering committee and was conducted according to Good Clinical Practice principles. In Thailand the trial was approved by the Faculty of Tropical Medicine Ethics Committee, Mahidol University, (reference TMEC 21-058) and by the Oxford University Tropical Research Ethics Committee (Oxford, UK; reference 24-21), and its results were reviewed regularly by a data and safety monitoring board. The protocol and statistical analysis plan are in the appendix (pp 35–152).

### Participants

Previously healthy adults aged 18–50 years were eligible for enrolment in the trial if they understood the procedures and requirements of the study and were able to give fully informed consent for full participation in the study; reported symptoms of COVID-19 for less than 4 days (<96 h); were SARS-CoV-2 positive, as defined either as a nasal lateral flow antigen test that became positive within 2 min (STANDARD Q COVID-19 Ag Test, SD Biosensor, Suwon-si, South Korea) or a positive PCR test with a cycle threshold value less than 25 (all viral gene targets) within the previous 24 h (both ensure the majority of recruited patients have high viral loads); had oxygen saturation 96% or higher measured by pulse oximetry at the time of screening; were unimpeded in activities of daily living; and agreed to adhere to all procedures, including availability and contact information for follow-up visits.

Exclusion criteria included taking any concomitant medications or drugs, chronic illness or condition requiring long-term treatment or other clinically significant comorbidity, laboratory abnormalities at screening (haemoglobin <8 g/dL, platelet count <50 000 per μL, abnormal liver function tests, and estimated glomerular filtration rate <70 mL/min per 1·73 m^2^), pregnancy (a urinary pregnancy test was performed in females), actively trying to become pregnant, lactation, contraindication or known hypersensitivity to any of the proposed therapeutics, currently participating in a COVID-19 therapeutic or vaccine trial, or evidence of pneumonia (although imaging was not required).

After a detailed explanation of study procedures and requirements all patients provided fully informed written consent. Sex data were reported as they were recorded on the participant’s identity documents.

### Randomisation and masking

Block randomisation was performed for each site via a centralised web app designed by MORU software engineers using RShiny, hosted on a MORU webserver. At enrolment, after obtaining fully informed consent and entering the patient details, the app provided the study drug allocation. The no study drug group comprised a minimum proportion of 20% of patients at all times, with uniform randomisation ratios applied across the active treatment groups (molnupiravir or ritonavir-boosted nirmatrelvir are reported here). Randomisation was done by the trial statistician. The laboratory team were masked to treatment allocation and the clinical investigators were masked to the virology results until the study group was terminated. Apart from the trial statistician (JAW), the clinical investigators were all masked to the quantitative PCR (qPCR) results.

For **RShiny** see https://shiny.posit.co/

### Procedures

All study drugs were stored under appropriate conditions. Molnupiravir (Lagevrio: Merck, Sharpe & Dohme, Rahaway, NJ, USA), and ritonavir-boosted nirmatrelvir (Paxlovid: Pfizer, New York, NY, USA) were given in standard doses (appendix p 9). Oral molnupiravir 800 mg was given twice a day for 5 days; oral nirmatrelvir 300 mg with oral ritonavir 100 mg was given twice a day for 5 days. Both medicines were provided through the Thailand Ministry of Public Health.

Prescreening occurred in the hospital’s acute respiratory infection unit. Potentially eligible participants were selected by the nurses on the unit to be contacted by the study team and were screened. Enrolled patients were admitted to the study ward or managed as outpatients, as per patient preference (none of the admissions were for clinical reasons, but for ease of adherence with the study procedures, or for self-isolation reasons). All treatments were directly observed. After randomisation and baseline procedures (appendix p 7) oropharyngeal swabs (two swabs from each tonsil) were taken as follows. A flocked swab (Thermo Fisher MicroTest [Thermo Fisher, Waltham, MA, USA] and later COPAN FLOQSwabs [COPAN Diagnostics, Murrieta, CA, USA]) was rotated against the tonsil through 360° four times and placed in Thermo Fisher M4RT (Thermo Fisher, Waltham, MA, USA) viral transport medium (3 mL). Swabs were transferred at 4–8°C, aliquoted, and then frozen at −80°C within 48 h. Separate swabs from each tonsil were taken once daily from day 0 to day 7, on day 10, and on day 14. Each swab was processed and tested separately. Vital signs were recorded three times daily by the patient (initial vital signs on the first day were recorded by the study team), and symptoms and any adverse effects were recorded daily.

The TaqCheck SARS-CoV-2 Fast PCR Assay (Applied Biosystems, Thermo Fisher Scientific, Waltham, MA, USA) quantitated viral loads (RNA copies per mL). This multiplexed real-time PCR method detects the SARS-CoV-2 N and S genes, and human RNase P gene in a single reaction. RNase P was used in the linear model to adjust for variation in sample human cell content (see appendix pp 22–24, 143–144). Viral loads were quantified against ATCC (Manassas, VA, USA) heat-inactivated SARS-CoV-2 (VR-1986HK strain 2019-nCoV/USA-WA1/2020) standards. Whole-genome sequencing was performed to identify viral variants and allocate genotypes (appendix pp 15–16).

### Outcomes

The primary outcome measure was the viral clearance rate between days 0 and 7.

All-cause admission to hospital for clinical deterioration (until day 28) was a secondary endpoint, as were determining optimal dosing and development of symptoms of the post-COVID-19 condition (also known as long-COVID; both will be reported elsewhere).

The prespecified endpoint of time to resolution of fever and time to resolution of symptoms were assessed using survival methods as the data were right-censored at the last visit. Patients were defined as febrile at baseline if at least one axillary temperature measurement within 24 h of randomisation was 37·5°C or higher. Resolution of fever was defined as an axillary temperature 37·0°C or lower for at least 24 h. Resolution of symptoms was defined as no reported symptoms.

Viral rebound was an exploratory outcome, and was defined as a mean daily oropharyngeal viral load which had declined to fewer than 100 genomes per mL for 2 or more consecutive days, followed by a viral load of more than 1000 genomes per mL at any timepoint thereafter.

Adverse events were graded according to the Common Terminology Criteria for Adverse Events version 5.0. Summaries were generated if the adverse event was grade 3 or worse and was new or had increased in intensity. Serious adverse events were recorded separately and reported to the data safety monitoring board.

### Statistical analysis

For each studied intervention the sample size was adaptive based on prespecified futility and success stopping rules (appendix pp 25–26).

The rate of viral clearance was expressed as a slope coefficient and estimated under a Bayesian hierarchical linear model (random effect terms for the individual patient slope and intercept).^12,13^ The model was fitted to the daily log_10_ oropharyngeal viral density measurements between days 0 and 7 (18 measurements per patient), using weakly informative priors and treating non-detectable viral densities (cycle threshold value 40) as left-censored (appendix pp 22–24).^12^ The treatment effect was defined as the multiplicative change (%) in the viral clearance rate, either relative to the no study drug group (when determining if an intervention had an antiviral effect), or relative to the positive control group (ritonavir- boosted nirmatrelvir).^13^ The viral clearance rate (ie, slope coefficient from the model fit) was also expressed as a clearance half-life (t_1/2_=log_10_ 0·5/slope). A 50% increase in clearance rate equals a 33% reduction in clearance half-life.

Comparison of times to resolution of fever and resolution of symptoms between different treatment groups used the log-rank test. For the analysis of viral rebound, comparison of proportions was done using the Fisher exact test.

Each studied intervention was compared only against its concurrent control, with interim analyses planned every additional ten patients recruited per group. In practice, the interim analyses were less frequent than planned as recruitment occurred very rapidly. Initially, all interim analyses compare the new intervention against the no study drug group. The intervention is dropped for futility when there is probability greater than 0·9 that the acceleration in viral clearance is less than 20% (this threshold was increased from 12·5% in January, 2023, statistical analysis plan version 3.0). If the new intervention reaches the success threshold (ie, probability >0·9 of acceleration in viral clearance >20% relative to no study drug), it is then compared with the positive control. This comparison terminates when the intervention is shown to be inferior, non-inferior, or superior to the positive control group using a 10% non-inferiority margin. If the intervention is superior, it then replaces the positive control group (appendix p 26). The non-inferiority component of the trial was added to the statistical analysis plan (version 3.0) in January, 2023. All stopping decisions are made using data from contemporaneously randomly assigned patients only. An individual patient data metaanalysis was performed using all data from unmasked interventions.

All efficacy analyses were done in a modified intention-to-treat population, comprising patients who had more than 2 days of follow-up data. Safety was analysed in all patients who received at least one dose of the study drug. A sensitivity analysis was performed using a non-linear model fitted to the serial viral densities, which allows for an initial increase followed by a log-linear decrease (exact specification is given in the appendix pp 22–24). All models included the virus variant (BA.2, BA.5, BA.4, BA.2.75, BQ.1, or XBB) as a covariate for the slope and intercept (appendix pp 27–28). Model fits were compared using approximate leave-one-out comparison as implemented in the package loo. All data analysis was done in R version 4.0.2. Posterior distributions were approximated using Hamiltonian Monte Carlo in Stan via the RStan interface.^17^ 4000 iterations were run over four independent chains with 2000 iterations for burn-in. Convergence was assessed visually from the trace plots (appendix p 32) and using the R-hat statistic (a value <1·1 was considered acceptable convergence).^18^ Goodness of fit was assessed by plotting the residuals over time and comparing the daily median model predictions with the observed values (appendix p 33). All point estimates are given with 95% credible intervals (CrIs), defined by the 2·5% and the 97·5% quantiles of the posterior distribution.

The ongoing platform trial is registered at ClinicalTrials.gov, NCT05041907.

### Role of the funding source

The funder of the study had no role in study design, data collection, data analysis, data interpretation, or writing of the report.

## Results

**Figure 1:**
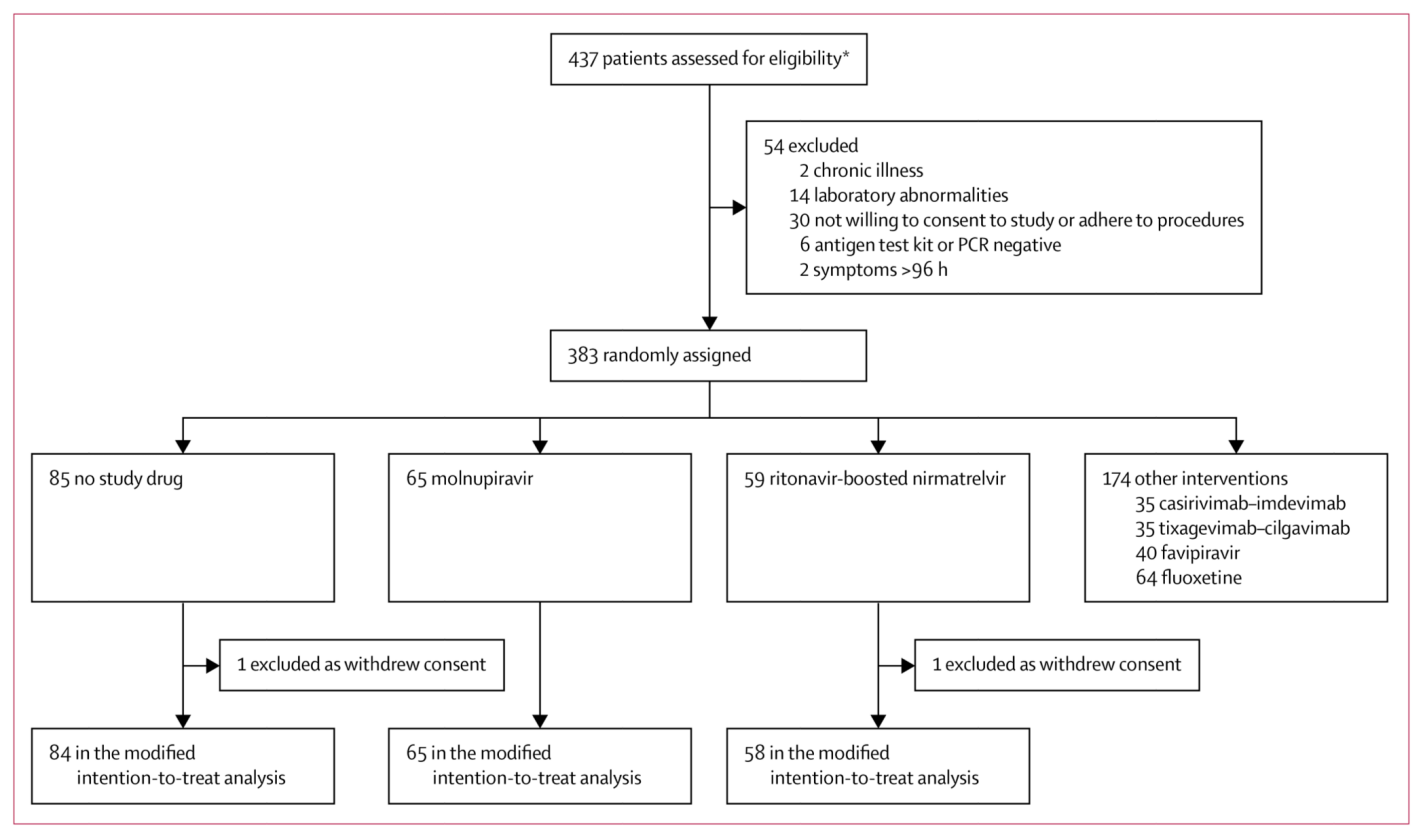
Trial profile *Prescreening occurred in the hospital’s acute respiratory infection unit. Potentially eligible participants were selected by the nurses to be contacted by the study team. As a result, a high proportion of those assessed for eligibility participated in the study. Both patients excluded from the modified intention-to-treat analysis did not receive trial medication.

The platform trial began recruitment on Sept 30, 2021. The molnupiravir and ritonavir-boosted nirmatrelvir groups started enrolment on June 6, 2022, when both drugs became available in Thailand. Initially, the prespecified interim analyses compared each drug individually with the concurrent no study drug group. By the fifth interim analysis (Aug 22, 2022, using data from 12 patients randomly assigned to ritonavir-boosted nirmatrelvir and 20 concurrent controls), ritonavir-boosted nirmatrelvir had met the stopping rule for success (probability >0·9 that viral clearance was increased by >12·5%). As a result, ritonavir-boosted nirmatrelvir remained in the platform trial but then became the positive control. Molnupiravir met the stopping rule for success at the sixth interim analysis (Oct 19, 2022; 28 patients had been randomly assigned to molnupiravir with 37 concurrent controls). Molnupiravir stayed in the trial, but then entered a non-inferiority comparison with ritonavir-boosted nirmatrelvir (appendix pp 26, 140). Molnupiravir was removed from the platform when the prespecified inferiority margin was met (Feb 23, 2023; seventh interim analysis) indicating that molnupiravir was inferior to ritonavir-boosted nirmatrelvir by at least 10%. By then, 59 patients had been randomly assigned to ritonavir-boosted nirmatrelvir, 65 to molnupiravir, and 85 to no study drug. Of the 209 patients randomly assigned, 80 (38%) were male and 129 (62%) were female. Two patients were excluded from the analyses because they withdrew from the study on day 0, resulting in a modified intention-to-treat population of 207 (figure 1).

**Figure 2:**
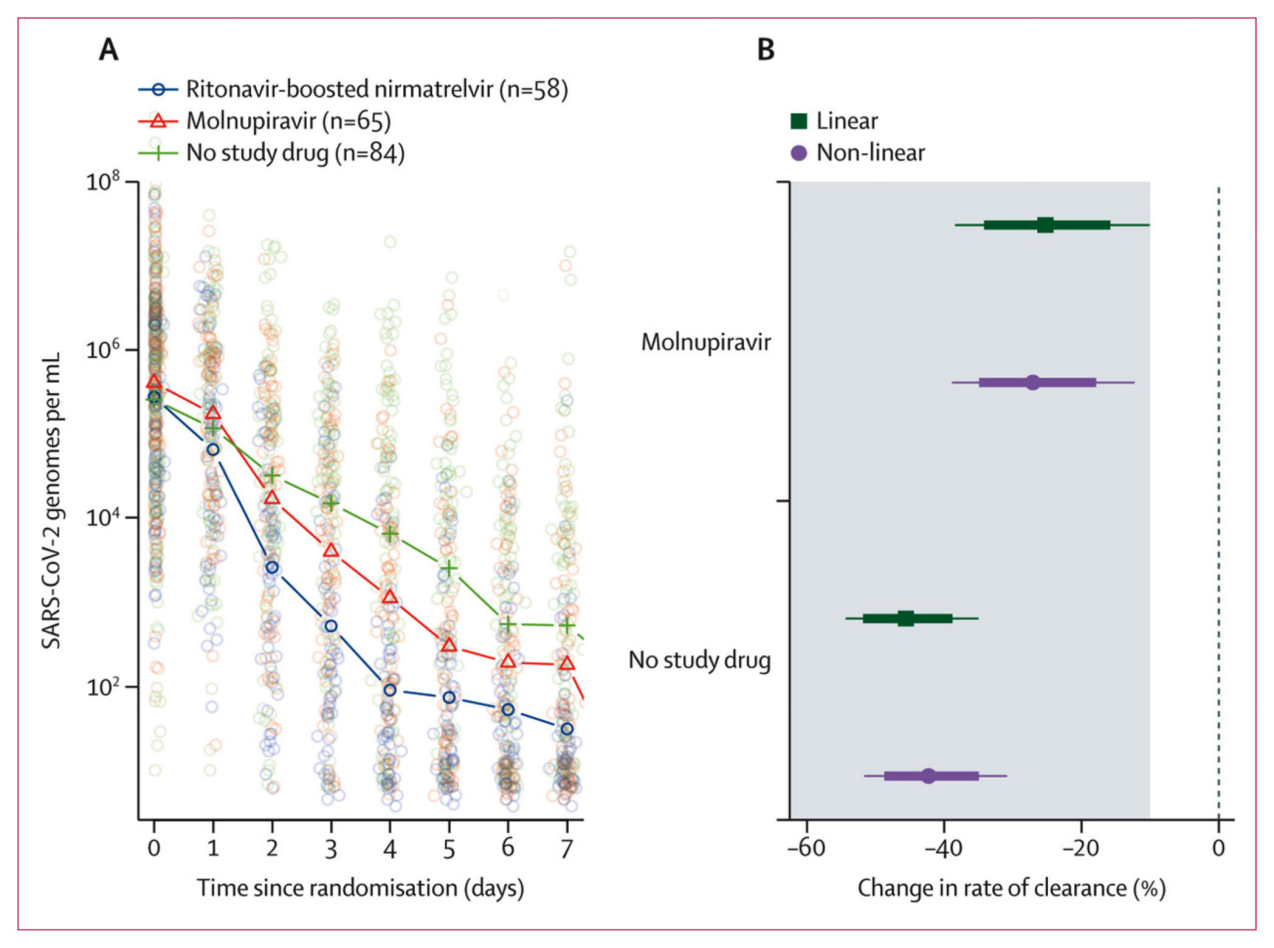
SARS-CoV-2 oropharyngeal viral clearance following ritonavir-boosted nirmatrelvir, molnupiravir, and no study drug (A) Median viral loads over time in the three contemporaneous treatment groups (individual data points shown as circles). (B) The estimated treatment effects relative to ritonavir-boosted nirmatrelvir under the linear and nonlinear models (the grey zone shows the inferiority zone relative to ritonavir-boosted nirmatrelvir).

In the modified intention-to-treat population there were 3704 qPCR viral density measurements (a median of 18 viral load estimates per patient [IQR 18–18] over 8 days) of which 2977 (80%) were above the lower limit of quantification. The median baseline oropharyngeal sample viral density was 10^5·7^ SARS-CoV-2 genomes per mL (IQR 10^4·8^ to 10^6·4^). Both drugs accelerated viral clearance compared with no treatment. Compared with patients receiving no study drug, by day 4 the median viral densities were 10-fold lower in the molnupiravir group and 100-fold lower in the ritonavir-boosted nirmatrelvir group (figure 2A). Under a linear model fitted to all viral load data up to day 7, relative to the no study drug group, the rates of viral clearance were 37% (95% CrI 16–65) faster with molnupiravir and 84% (54–119) faster with ritonavir-boosted nirmatrelvir. In the non-inferiority comparison, viral clearance was 25% (10–38) slower with molnupiravir than ritonavir-boosted nirmatrelvir. The viral clearance with molnupiravir was less than the non-inferiority margin of 10% relative to ritonavir-boosted nirmatrelvir with a probability of 0·98. The non-linear model gave near identical results (figure 2B).

**Figure 3:**
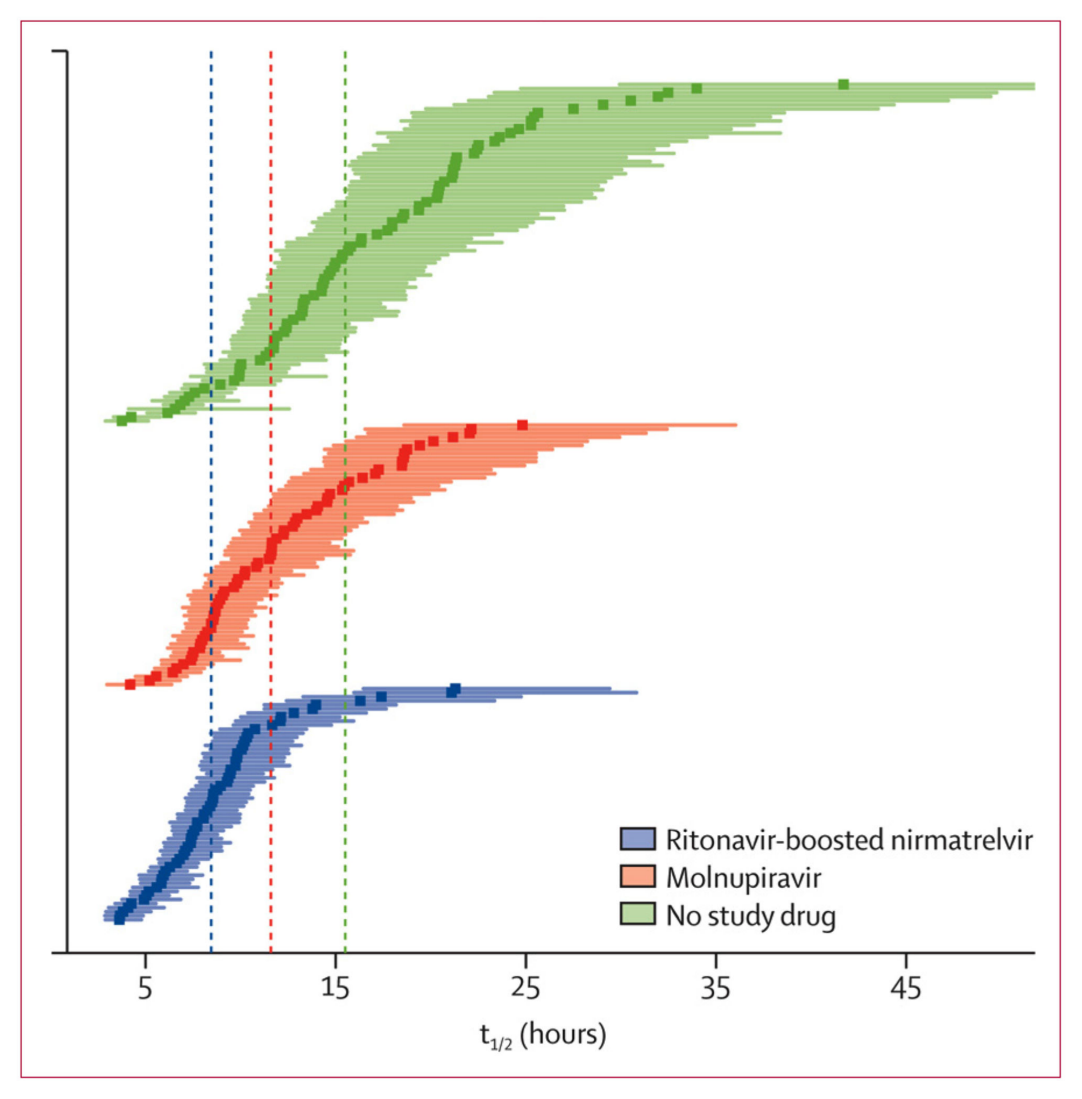
Individual patient estimated virus clearance half-lives by treatment group Point estimates and 80% credible intervals are shown for legibility. The vertical dashed lines show the median half-lives in each group.

Median estimated viral clearance half-lives under the linear model were 8·5 h (IQR 6·7–10·1) with ritonavir-boosted nirmatrelvir, 11·6 h (8·6–15·4) with molnupiravir, and 15·5 h (11·9–21·2) in the contemporaneous no study drug group. Median virus clearance half-life was nearly halved by ritonavir-boosted nirmatrelvir and reduced by one third by molnupiravir, compared with no study drug (figure 3).

No patients developed severe disease or were hospitalised. There were no significant differences in fever clearance across the three intervention groups (although power was low as only a third of patients had fever at baseline; appendix p 29). Time to symptom resolution was faster in the molnupiravir and ritonavir-boosted nirmatrelvir groups compared with the no study drug group (p=0·011; appendix p 30).

**Figure 4:**
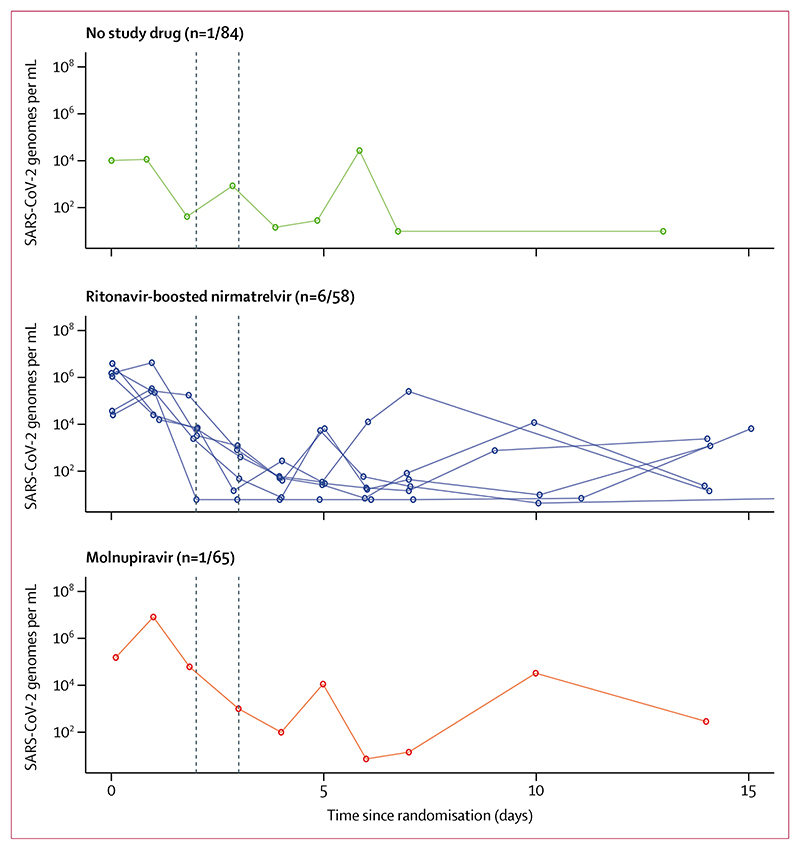
Exploratory analysis of viral rebound Individual oropharyngeal viral load profiles in patients who had a viral rebound under the prespecified definition (viral load <100 genomes per mL for ≥2 consecutive days, followed by a viral load >1000 genomes per mL at any later timepoint).

In an exploratory analysis, a higher proportion of patients had viral rebound in the ritonavir-boosted nirmatrelvir group (six [10%] of 58) compared with the molnupiravir group (one [2%] of 65; p=0·051) or the no study drug group (one [1%] of 84; p=0·018; figure 4). Of these eight patients, three reported evidence of symptom rebound, all in the ritonavir-boosted nirmatrelvir group.

The oropharyngeal swabbing procedure and all treatments were well tolerated. Patients receiving ritonavir-boosted nirmatrelvir commonly complained of dysgeusia but none discontinued treatment as a result. There were no treatment-related serious adverse events (table and appendix pp 11–13).

**Figure 5:**
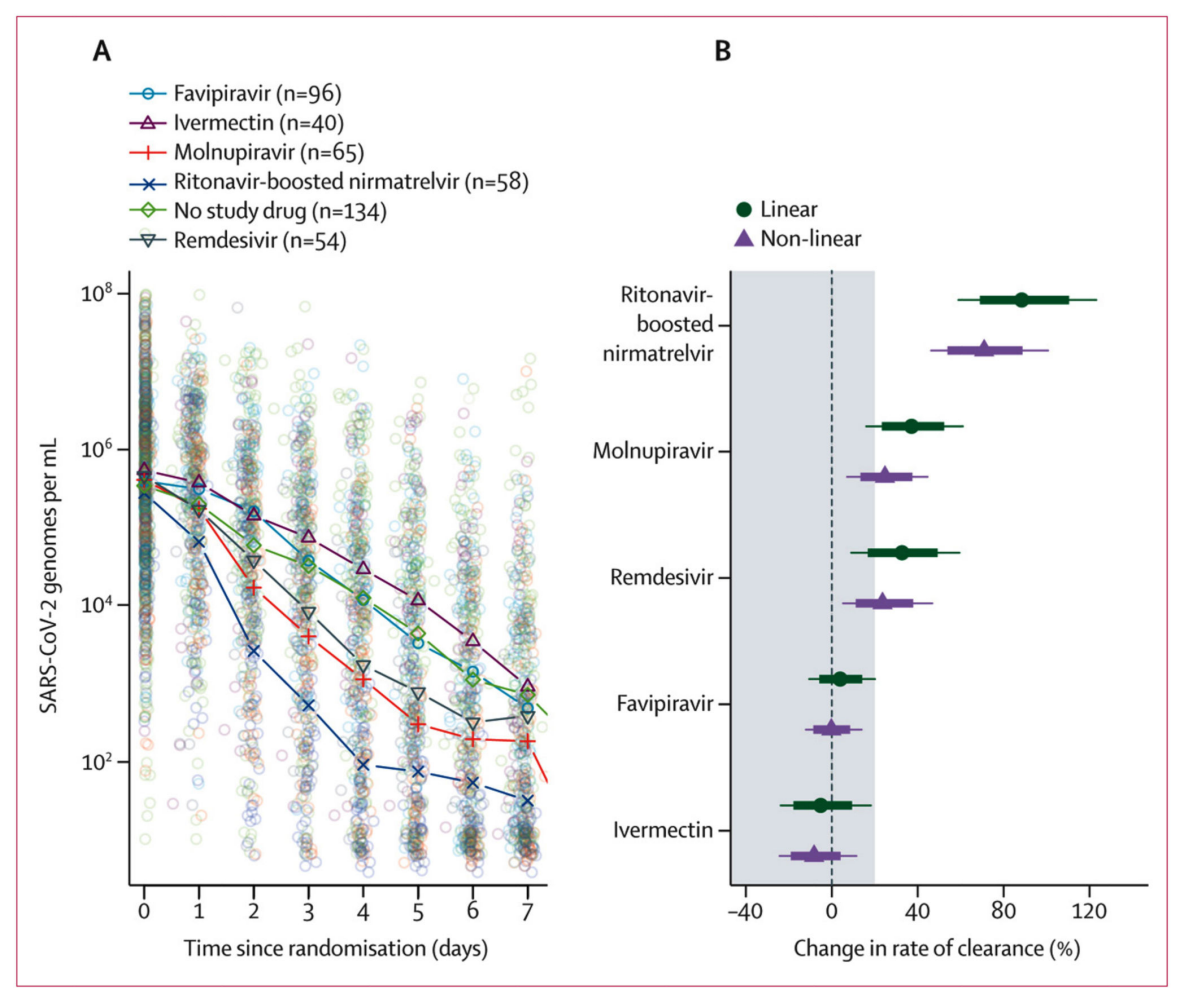
Meta-analysis of oropharyngeal viral clearance in 447 patients enrolled in the same site in Thailand (not all concurrently) (A) Daily median oropharyngeal viral loads by treatment group. (B) Estimated treatment effect on viral clearance rate relative to no study drug under a model adjusting for study epoch and virus variant.

To compare antiviral effects of all the unmasked small molecule drugs tested in the PLATCOV platform trial, we performed an individual patient data meta-analysis using patients recruited to the same centre in Thailand. This comprised recipients of ivermectin,^12^ remdesivir,^14^ favipiravir,^15^ molnupiravir, ritonavir-boosted nirmatrelvir, or no study drug (fluoxetine remained masked). The analysis population comprised 447 patients randomly assigned between Sept 30, 2021, and Feb 7, 2023, with a total of 8032 qPCR measurements (6944 [86%] above the lower limit of quantification). Because the interventions were not randomised concurrently, and thus temporal confounding is expected, the analysis adjusted both for calendar time (by adding a covariate on the slope and intercept) and for virus lineage. The no study drug group spanned the entire study period. Under the linear model, the two interventions reported previously to have no clinical antiviral effect, ivermectin and favipiravir,^12,15^ had very similar virus clearance rates to the no study drug group (figure 5). Remdesivir, previously reported to have a moderate effect on viral clearance,^14^ was estimated to increase viral clearance relative to no study drug by 33% (95% CrI 9–59). In comparison, molnupiravir was estimated to increase viral clearance by 37% (16–61), with a probability of superiority compared with remdesivir of 0·61 (ie, very similar treatment effects to molnupavir). In the overall comparison, ritonavir-boosted nirmatrelvir increased viral clearance relative to no study drug by 88% (59–123; probability of superiority compared with remdesivir=1). Symptom clearance is shown in the appendix (p 31).

61 samples (1–6 per individual) taken on day 7 or later from 31 individuals with persistent oropharyngeal swab cycle threshold values less than 35, were sequenced successfully. Three of these individuals received ritonavir-boosted nirmatrelvir, nine received molnupiravir, and 19 received no study drug. All baseline isolates were also sequenced. An increased number of single nucleotide polymorphisms (SNPs) relative to baseline was found in three of the nine molnupiravir isolates from day 7 or later and none in the ritonavir-boosted nirmatrelvir or no study drug samples (appendix p 19).

Among these three isolates, there were three branches containing ten or more SNPs. A previously identified mutational signature associated with molnupiravir is characterised by excess transition mutations which are mainly C to T and G to A mutations.^9^ All the SNPs identified on these branches were transitions, with C to T and G to A being the dominant mutations (appendix p 19). In one case the consensus genome at day 7 had 19 extra mutations compared with day 0. In another case, two separate consensus genomes with excess mutations were observed: at day 7 with 16 mutations and day 14 with 29 mutations (appendix p 19).

A search of publicly available genome databases did not find any evidence of onward transmission of these strains. Using public datasets on predicted fitness effects of amino acid mutations there were a small number of mutations with elevated predicted fitness, but all three strains scored lower than the parent day 0 strain (appendix pp 17–18).

## Discussion

To our knowledge, this is the first comparative pharmacodynamic assessment of COVID-19 antiviral treatments in patients. The study shows that both molnupiravir and ritonavir-boosted nirmatrelvir accelerate viral clearance in early COVID-19. This supports the results of previous clinical trials and studies with sparse viral sampling. The antiviral effect of ritonavir-boosted nirmatrelvir was substantially greater than that of molnupiravir. This corresponds approximately with the differences in clinical benefits reported in earlier randomised and observational studies.^3–5,19^ It suggests that inhibiting the main viral protease provides the most potent inhibition of viral replication in patients with COVID-19. Other main protease inhibitors, which do not require pharmacokinetic boosting by ritonavir, are in pharmaceutical development.^20,21^ The meta-analysis of this platform trial indicated that the molnupiravir effect was very similar to parenteral remdesivir, although this comparison is less robust as the two drugs were not compared contemporaneously.^14^

The main indication for oral antiviral treatment in patients with COVID-19 is prevention of disease progression.^1,4,5,22^ As with other potentially serious infections, the earlier in the course of disease that effective antiviral drugs are given, the greater the clinical benefit.^23^ Several different antiviral medicines have proved effective in patients with COVID-19, but there have been no direct comparisons between them. It has become increasingly difficult to conduct the large clinical trials with clinical endpoints in patients with COVID-19 that have been used to inform treatment policies. The low rates of hospitalisation and death, and the imprecision of clinical and laboratory measures of recovery, have resulted in the failure of large outpatientbased studies to reach their prespecified endpoints. Acceleration of SARS-CoV-2 clearance correlates with prevention of hospitalisation and death,^1,4,5,24^ and provides an efficient pharmacodynamic measure of comparative antiviral efficacy. Compared with trials with clinical endpoints, the pharmacometrics approach used here requires two orders of magnitude fewer enrolled patients to provide comparative assessments, and it delivers results rapidly in real time.

A mutational signature associated with molnupiravir-treated SARS-CoV-2 viruses has been identified previously in the AGILE CST-2 clinical trial,^25^ and sequence branches suggestive of this signature have been seen in global surveillance samples.^9^ We show that viral genomes with this signature can be isolated from patients treated with molnupiravir, and are present in patients at allele frequencies high enough to be detected by the consensus sequences seen in global surveillance. Despite the decreased viral load resulting from molnupiravir treatment, a few individuals did maintain oropharyngeal viral loads high enough to perform wholegenome sequencing at later timepoints. The highly mutated sequences were detectable for up to 9 days after stopping treatment. However, no evidence of onward transmission of any of these variants could be found in the publicly available databases, and the predicted fitness effects of these mutations were deleterious overall.

Despite characterising comparative antiviral efficacy satisfactorily with small patient numbers, this study has several limitations. It is a single-centre study. Population differences in immune status and pharmacokinetics might affect therapeutic responses. We intentionally evaluated the interventions in adults at low risk with high viral burdens in order to optimise the comparative assessment of the different drugs, and not in patients at high risk or older patients who are at greatest risk of disease progression. Protection against severe disease could not be assessed in this study as no-one was hospitalised. Although the rates of symptom resolution and viral clearance were correlated, only a third of patients were febrile initially, so correlates of fever clearance were not established reliably. Viral rebound was noted although the study was not designed to characterise this fully. The analysis population was described as a modified intention-to-treat population, which excluded randomly assigned patients who provided insufficient data for analysis of viral clearance (only two were excluded). This could in theory introduce bias by selecting on a post-randomisation event; however, the primary endpoint was virological (and masked to the investigators), so this is highly unlikely.

The considerable intra-individual variability in nasopharyngeal (or oropharyngeal) viral loads results in a low signal-to-noise ratio. Frequent sampling over 5–7 days is therefore required to measure clearance rates. Oropharyngeal sampling is well tolerated, whereas frequent nasopharyngeal sampling is not. This simple approach allows pharmacometric characterisation and comparison of antivirals with patient numbers usually of between 30 and 100 per group.^12,13^ It is readily conducted anywhere where accurate qPCR viral quantitation can be performed. Pharmacometric assessment can also be used to characterise dose–response relationships, thereby informing dosing and therapeutic practice in real time. Regulatory authority and treatment guideline decisions should be based primarily upon in-vivo evidence and should consider viral clearance as a surrogate for clinical antiviral activity.

For the **information stored on Zenodo** see https://doi.org/10.5281/zenodo.8381828

For the MORU Tropical Health Network site see https://www.tropmedres.ac/units/morubangkok/bioethics-engagement/data-sharing

## Supplementary Material

Appendix

## Figures and Tables

**Table:** Baseline patient characteristics in the modified intention-to-treat population, and safety information in those who received at least one dose of study medication

	Ritonavir-boosted nirmatrelvir group (n=58)	Molnupiravir group (n=65)	No study drug group (n=84)
Age, years	29 (26-35)	30 (26-36)	29 (24-36)
BMI, kg/m^2^	22·5 (21·0-24·5)	22·3 (20·3-25·8)	22·5 (20·0-25·4)
Oropharyngeal viral load, log_10_ copies per mL	5·4 (4·7-6·3)	5·8 (5·0-6·4)	5·6 (4·7-6·3)
Sex			
Female	35 (60%)	37 (57%)	57 (68%)
Male	23 (40%)	28 (43%)	27 (32%)
SARS-CoV-2 variant			
BA.2	1 (2%)	5 (8%)	10 (12%)
BA.5	25 (43%)	28 (43%)	37 (44%)
BA.4	3 (5%)	2 (3%)	2 (2%)
BA.2.75	27 (47%)	27 (42%)	33 (39%)
BQ.1	1 (2%)	0	1 (1%)
XBB	1 (2%)	3 (5%)	1 (1%)
Duration of symptoms, days	2 (2–2)	2 (2–2)	2 (1–3)
Any vaccine received	58 (100%)	64 (99%)	84 (100%)
Number of mRNA vaccine doses			
0	7 (12%)	9 (14%)	7 (8%)
1	25 (43%)	27 (42%)	26 (31%)
2	22 (38%)	23 (35%)	43 (51%)
3	4 (7%)	6 (9%)	8 (10%)
Any adverse event, grade ≥3	0	0	0
Serious adverse event reported	0	0	0

Data are median (IQR) or n (%).

## Data Availability

All code and de-identified participant data required for replication of the study’s endpoints are openly accessible via Zenodo, as well as the study protocol and statistical analysis plan, from publication date onwards. Individual patient data can be requested and may be shared according to the terms defined in the MORU data sharing policy with other researchers to use in the future from the date of publication. Further information on how to apply is on the MORU Tropical Health Network site.

## Research in context

### Evidence before this study

Molnupiravir and ritonavir-boosted nirmatrelvir are the two leading oral antiviral drugs for patients with COVID-19. We searched PubMed for studies published in English from Jan 1, 2020, to July 11, 2023, using the terms: “randomised” AND [“nirmatrelvir OR paxlovid”] AND “molnupiravir”. Both molnupiravir and nirmatrelvir have shown in-vivo antiviral activity and clinical benefit, but there have been no direct randomised head-to-head comparisons. A network metaanalysis suggested that nirmatrelvir is slightly more effective than molnupiravir. Comparisons between the preregistration studies are confounded by substantial differences in the study populations, and timing of the studies. Associations between the use of these drugs and the risk of viral mutations (molnupiravir) and viral rebound (nirmatrelvir) have both been reported, although their incidence and impact are uncertain.

### Added value of this study

For COVID-19, comparison of antiviral drugs using clinical endpoints such as hospitalisation or death now requires prohibitively large sample sizes because these outcomes have become increasingly rare. By contrast, the pharmacometric approach described here provides a quantitative measure of antiviral effects in patients with tractable sample sizes. To our knowledge, this randomised study provides the first direct comparison of the in-vivo antiviral effects of molnupiravir and ritonavir-boosted nirmatrelvir. It shows that both drugs accelerate SARS-CoV-2 viral clearance but that ritonavir-boosted nirmatrelvir results in faster clearance than molnupiravir; that viral rebound occurs relatively often in a healthy population following ritonavir-boosted nirmatrelvir; and that mutant viruses are generated frequently following molnupiravir treatment.

### Implications of all the available evidence

Ritonavir-boosted nirmatrelvir has superior in-vivo antiviral activity in patients with early COVID-19 compared with molnupiravir. Candidate antivirals and antiviral combinations for COVID-19 should be assessed and compared using this method, which might also be applicable to other acute respiratory infections.
