# Supplementary material for: Antiviral efficacy of molnupiravir versus ritonavir-boosted nirmatrelvir in patients with early symptomatic COVID-19 (PLATCOV): an open-label, phase 2, randomised, controlled, adaptive trial
Source: Lancet Infect Dis. Author manuscript; Available in PMC 2024 Jan 1. (PMC7615401; doi:10.1016/S1473-3099(23)00493-0)
Supplement: Appendix [file EMS190138-supplement-Appendix.pdf]

THE LANCET
Infectious Diseases

Supplementary appendix

This appendix formed part of the original submission and has been peer reviewed. We post it as supplied by the authors.

Supplement to: Schilling WHK, Jittamala P, Watson JA, et al. Antiviral efficacy of molnupiravir versus ritonavir-boosted nirmatrelvir in patients with early symptomatic COVID-19 (PLATCOV): an open-label, phase 2, randomised, controlled, adaptive trial. *Lancet Infect Dis* 2023; published online Sept 28. https://doi.org/10.1016/S1473-3099(23)00493-0.

Supplementary appendix

Antiviral efficacy of molnupiravir versus ritonavir-boosted nirmatrelvir in patients with early symptomatic COVID-19 (PLATCOV): an open-label, phase 2, randomised, controlled, adaptive trial

List of Sites and Investigators (PLATCOV Collaborative Group) 2
Ethics Approval 6
Baseline procedures 7
Vaccines received prior to study enrolment 8
Molnupiravir additional information 9
Ritonavir-boosted nirmatrelvir additional information 9
Symptoms included in symptom questionnaire performed during study visits 10
Adverse events (AE) for molnupiravir and ritonavir-boosted nirmatrelvir 11
Symptoms reported for patients with virological rebound 14
Virus variant determination 15
Predicted fitness changes in detectable virus isolates in those with additional mutations at later timepoints 17
Analysis of the molnupiravir mutation signature 19
Randomisation 20
Statistical Analysis 22
Stopping rules 25
Supplementary Results 27
References: 34
Master Protocol Version 4.0 / 17 August 2022 35
PLATCOV Statistical Analysis Plan Version 3.0 / 12 January 2023 133

List of Sites and Investigators (PLATCOV Collaborative Group)

Sites

1. Hospital for Tropical Diseases (HTD), Faculty of Tropical Medicine, Mahidol University, 420/6 Rajvithi Road, Bangkok, 10400, Thailand
2. Vajira Hospital (VJ), Navamindradhiraj University, 681 Samsen road, Dusit, Bangkok, 10300, Thailand
3. Bangplee Hospital (BP), 88/1 Moo 8 Tambon Bang Phli Yai, Amphoe Bangplee, Samut Prakan 10540, Thailand
4. Mahosot Hospital, Quai Fa Ngum, Vientiane, Laos

Investigators

Co-principal investigators:

Nicholas J White (nickw@tropmedres.ac)[1,2]

William Schilling (william@tropmedres.ac)[1,2]

Faculty of Tropical Medicine, Mahidol University:

Site and Country Principal investigator:

Weerapong Phumratanaprapin[4]

Accountable Investigator:

Viravarn Luvira[4]

Study co-Investigators:

James Callery[1,2]

Nicholas PJ Day[1,2]

Sasithon Pukrittayakamee[1,4]

Simon Boyd[1,2]

Cintia Cruz[1,2]

Arjen M Dondorp[1,2]

Walter RJ Taylor[1,2]

James A Watson[1,2]

Watcharapong Piyaphanee[4]

Kittiyod Poovorawan[1,4]

Thundon Ngamprasertchai[4]

Tanaya Siripoon[4]

Borimas Hanboonkunupakarn[1,4]

Kesinee Chotivanich[1,2]

Podjanee Jittamala[1,3]

Mallika Imwong[1,5]

Janjira Thaipadungpanit[1,4]

Maneerat Ekkapongpisit[1]

Varaporn Kruabkontho[1]

Thatsanun Ngernseng[1]

Jaruwan Tubprasert[1]

Mohammad Yazid Abdad[1,2]

Elizabeth M Batty[1,2]

Shivani Singh [1,2]

Vajira Hospital:

Site Principal investigator:

Vasin Chotivanich[7]

Co-investigators:

Wiroj Ruksakul[7]

Chunlanee Sangketchon[8]

Bangplee Hospital:

Site Principal investigator:

Pongtorn Hanboonkunupakarn[6]

Co-investigator:

Sakol Sookprome[6]

Brazil Site (Universidade Federal de Minas Gerais):

Site and Country Principal investigator:

Mauro M Teixeira[9]

Co-Investigators:

Pedro J Almeida[9]

Renato S Aguiar[10]

Franciele M Santos[10]

Laos Site – Mahosot Hospital

Elizabeth Ashley[2,11]

Manivanh Vongsouvath[11]

Koukeo Phommasone[11]

Audrey Dubot-Pérès[11]

Sisouphanh Vidhamaly[12]

Ammala Chingsanoon[12]

Sixiong Bisayher[12]

Danoy Chommanam[11]

Terry John Evans[11]

Vayouly Vidhamaly[11]

Latsaniphone Boutthasavong[11]

Mayfong Mayxay[11]

Ministry of Public Health, Thailand

Manus Potaporn[13]

Attasit Srisubat[13]

Bootsakorn Loharjun[13]

1. Mahidol Oxford Tropical Medicine Research Unit, Faculty of Tropical Medicine, Mahidol University, Bangkok, Thailand

2. Centre for Tropical Medicine and Global Health, Nuffield Department of Medicine, Oxford University, Oxford, UK

3. Department of Tropical Hygiene, Faculty of Tropical Medicine, Mahidol University, Bangkok, Thailand

4. Department of Clinical Tropical Medicine, Faculty of Tropical Medicine, Mahidol University, Bangkok, Thailand

5. Department of Molecular Tropical Medicine and Genetics, Faculty of Tropical Medicine, Mahidol University, Bangkok, Thailand

6. Bangplee Hospital, Ministry of Public Health, Samut Prakarn province, Thailand

7. Faculty of Medicine, Navamindradhiraj University, Bangkok, Thailand

8. Faculty of Science and Health Technology, Navamindradhiraj University, Bangkok,Thailand

9. Clinical Research Unit, Center for Advanced and Innovative Therapies, Universidade Federal de Minas Gerais, Minas Gerais, Brazil

10. Department of Genetics, Ecology and Evolution, Institute of Biological Sciences, Universidade Federal de Minas Gerais, Minas Gerais, Brazil

11. Lao-Oxford-Mahosot Hospital-Wellcome Trust Research Unit, Mahosot Hospital, Vientiane, Lao PDR

12. Mahosot Hospital, Quai Fa Ngum, Vientiane, Lao PDR

13. Department of Medical Services, Ministry of Public Health, Nonthaburi, Thailand

Ethics Approval

The trial was approved by local and national research ethics boards in Thailand (Faculty of Tropical Medicine Ethics Committee, Mahidol University, FTMEC Ref: TMEC 21-058) and the Central Research Ethics Committee (CREC, Bangkok, Thailand, CREC Ref: CREC048/64BP-MED34), in Laos by the National Ethics Committee for Health Research (NECHR, Lao People's Democratic Republic, Submission ID 2022.48) and the Federal Drug Administration (FDA, Lao People's Democratic Republic, 13066/FDD_12Dec2022) and by the Oxford University Tropical Research Ethics Committee (OxTREC, Oxford, UK, OxTREC Ref: 24-21).

Baseline procedures

Baseline investigations included a full clinical examination, rapid SARS-CoV-2 antibody test (BIOSYNEX COVID-19 BSS IgM/IgG®, Illkirch-Graffenstaden, France, done in Thailand only), blood sampling for haematology and biochemistry, an electrocardiogram and a chest radiograph (following local guidance in Thailand, but not a study requirement).

Vaccines received prior to study enrolment

Vaccine combination	Number of patients
Astrazeneca only	8
Astrazeneca/Moderna	17
Astrazeneca/Moderna/Pfizer	6
Astrazeneca/Moderna/Pfizer/Sinovac	4
Astrazeneca/Moderna/Sinopharm	1
Astrazeneca/Moderna/Sinovac	8
Astrazeneca/Pfizer	49
Astrazeneca/Pfizer/Sinopharm	2
Astrazeneca/Pfizer/Sinovac	32
Astrazeneca/Sinovac	7
Moderna only	3
Moderna/Pfizer	2
Moderna/Pfizer/Sinopharm	3
Moderna/Pfizer/Sinovac	6
Moderna/Sinopharm	6
Moderna/Sinovac	3
Pfizer only	5
Pfizer/Sinopharm	8
Pfizer/Sinovac	29
Sinopharm only	6
Sinovac only	1

Molnupiravir additional information

Molnupiravir- Lagevrio™ (Merck & Co. Inc. New Jersey, USA) was given orally at a dose of 800mg twice a day for a total of five days.

Ritonavir-boosted nirmatrelvir additional information

Nirmatrelvir/ritonavir- Paxlovid™ (Pfizer, New York, USA) was given orally at a dose of 300mg of nirmatrelvir with one 100mg tablet of ritonavir twice a day for a total of five days.

Symptoms included in symptom questionnaire performed during study visits

- Fever
- Headache
- Dizziness
- Blurred vision
- Fatigue
- Cough
- Difficulty breathing
- Chest pain
- Runny nose
- Loss of smell or taste
- Abdominal pain
- Loss of appetite
- Nausea
- Vomiting
- Diarrhoea
- Arthralgia
- Myalgia
- Itching
- Skin rash
- Sore throat
- Other

Adverse events (AE) for molnupiravir and ritonavir-boosted nirmatrelvir

	All grades			Grade 3-4		
	**Molnupiravir (N=65)**	**Nirmatrelvir/ ritonavir (n=59)**	**No study drug (n=85)**	**Molnupiravir (N=65)**	**Nirmatrelvir/ ritonavir (n=59)**	**No study drug (n=85)**
Any adverse event (Grade ≥ 3)	0	0	0			
Serious adverse event reported	0	0	0			
Symptoms						
Fever				0	0	0
Headache				0	0	0
Dizziness				0	0	0
Blurred vision				0	0	0
Fatigue				0	0	0
Cough				0	0	0
Difficulty breathing				0	0	0
Chest pain				0	0	0
Runny nose				0	0	0
Loss of smell or taste				0	0	0
Abdominal pain				0	0	0
Loss of appetite				0	0	0

Nausea				0	0	0
Vomiting				0	0	0
Diarrhoea				0	0	0
Arthralgia				0	0	0
Myalgia				0	0	0
Itching				0	0	0
Skin rash				0	0	0
Laboratory abnormalities						
Creatinine				0	0	0
BUN				0	0	0
Sodium				0	0	0
eGFR				0	0	0
Potassium				0	0	0
ALT/SGPT				0	0	0
AST/SGOT				0	0	0
Total bilirubin				0	0	0
Direct bilirubin				0	0	0
Alkaline Phosphatase				0	0	0
LDH				0	0	0

Creatinine phosphokinase (CPK)				0	0	0
Anaemia				0	0	0
Leukocytopaenia				0	0	0
Neutropaenia				0	0	0
Thrombocytopaenia				0	0	0

Table S1: Summary of adverse events (grade 3 and above) for molnupiravir and ritonavir-boosted nirmatrelvir in the PLATCOV study. All AEs solicited were reported between days 0-7, day 14 and day 28.

Symptoms reported for patients with virological rebound

Three of the eight patients who had virological rebound (all in the nirmatrelvir arm) reported a return of symptoms, after these had initially resolved on treatment. In the first, the patient had a cough on days 1-3 then again at day 14. The second experienced cough on days 1,2,3 then again at 7 and 14. The third patient reported a return of URTI symptoms (cough, sore throat, runny nose) on day 14, and additional post-exertional fatigue on day 28).

Virus variant determination

SARS-CoV-2 whole-genome sequencing

The sequencing method follows the "PCR tiling of SARS-CoV-2 virus with rapid barcoding and Midnight RT PCR Expansion" provided by Oxford Nanopore Technology (Oxford, UK) developed based on a protocol by the ARTIC network group.[1] Library preparation process started with reverse transcription, which consists of mixing the purified viral RNA with LunaScript RT SuperMix and incubating the mixtures in a thermal cycler. DNA fragments to be used in the assembly process were amplified by PCR using Midnight primer set (V3) and attached with barcodes from Rapid Barcode Plate (RB96). The mixtures from each sample were pooled together, cleaned with AMPure XP Beads (AXP) and attached with Rapid Adapter F (RAP F). The prepared DNA fragments were then loaded into a primed flow cell (FLO-MIN106) and sequenced on GridION MK1 system.

Viral genome assembly and classification

The output sequencing data (.fast5) from MinKNOW software was base-called with Guppy software using the High Accuracy (HAC) model to generate nucleotide sequence data for each fragment (reads) in the fastq format. These base-called data were then processed through the established workflow wf-artic on EPI2ME software to be assembled into consensus sequences. Only reads with average Phred Quality (Q) score above 9 and minimum and maximum length of 250 and 1500 bps were used in the assembly process. The consensus sequences were then classified using the Pangolin tool (4.1.1) and Pangolin dataset (v1.14).

We sequenced all samples taken at day 7 or later timepoints in individuals with viral rebound where the Ct of the sample was below 35; a total of 123 samples from 38 patients. 66

sequences passing QC were obtained from 31 patients. For each patient we aligned the consensus sequences using MAFFT and identified the number of SNPs between the day 0 sequence and any later timepoints using snp-dists.[2] Nextclade was used to identify nucleotide and amino acid changes and mutations were visualised using Snipit.[3,4] The Newick tree generated by Nextclade was used to visualise the phylogeny. LoFreq was used to call SNVs and to determine minor allele frequencies in the patient with mixed genotypes.[5] Fitness effects of amino acid mutations were taken from the estimates of Neher and Bloom.[6,7] To look for potential transmission of molnupiravir associated genotypes, we took the pre-processed mutation annotated tree of all public genomes on March 9th, 2023.[8] We selected all samples from GISAID which were collected after the collection date of our samples and were either collected in Thailand or belonged to the same PANGO lineage as the molnupiravir associated genomes, and added them to this tree using UShER to identify any samples which may have been transmitted from our genomes.[9]

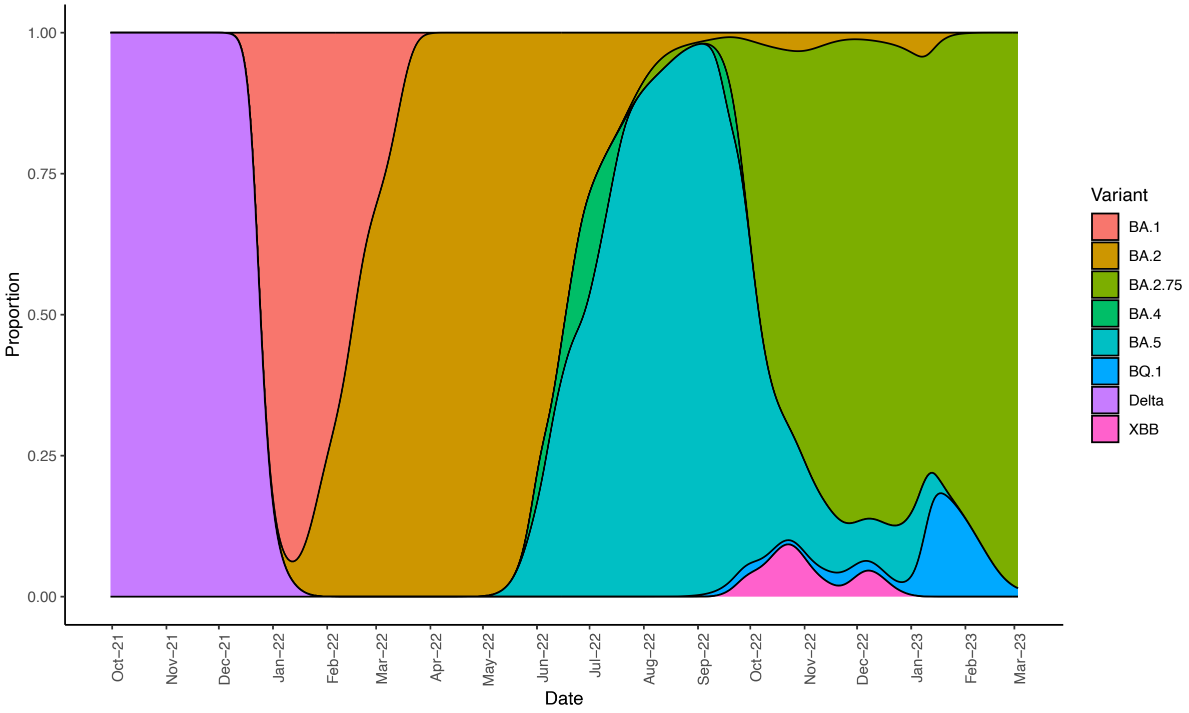

Figure S1 Proportion of SARS CoV2 variants identified in the enrolled patients over time (664 individuals from Thailand site only). Individuals with failed sequencing results are excluded from the data.

Predicted fitness changes in detectable virus isolates in those with additional mutations at later timepoints

Individual	Timepoint	AA change	AA change by gene	Fitness Score
490	Day 7	ORF1a:T170I	nsp1:T170I	-0.86305
490	Day 7	S:V503I	S:V503I	-0.94138
490	Day 7	ORF1a:L122F	nsp1:L122F	-1.0168
490	Day 7	ORF1a:A2249T	nsp3:A1431T	-1.1441
490	Day 7	ORF1a:V830M	nsp3:V12M	-1.2383
490	Day 7	ORF1a:M2719I	nsp3:M1901I	-2.1336
612	Day 14	ORF1b:A2132V	nsp15:A81V	0.86749
612	Day 14	ORF1a:T727I	nsp2:T547I	0.73993
612	Day 14	ORF1b:M1839I	nsp14:M315I	0.055615
612	Day 14	ORF1b:F68S	nsp12:F77S	N/A
612	Day 14	ORF1a:G7D	nsp1:G7D	-0.11527
612	Day 14	ORF1a:V1866I	nsp3:V1048I	-0.14793
612	Day 14	ORF1a:G572E	nsp2:G392E	-0.15626
612	Day 14	ORF1a:R2336K	nsp3:R1518K	-0.16467
612	Day 14	ORF3a:P42S	ORF3a:P42S	-0.37172
612	Day 14	S:E180K	S:E180K	-0.52678
612	Day 14	S:R847K	S:R847K	-0.65599
612	Day 14	ORF1a:T2611I	nsp3:T1793I	-0.71384
612	Day 14	ORF1a:C1235R	nsp3:C417R	-0.83465
612	Day 14	S:G1246D	S:G1246D	-0.9232
612	Day 14	ORF8:H28Y	ORF8:H28Y	-1.6654
612	Day 7	ORF1b:A2132V	nsp15:A81V	0.86749
612	Day 7	ORF1a:T224I	nsp1:T44I	N/A
612	Day 7	ORF1b:V467I	nsp12:V476I	N/A
612	Day 7	ORF3a:H93Y	ORF3a:H93Y	-0.091452
612	Day 7	ORF1a:V3048I	nsp4:V285I	-0.50283

612	Day 7	ORF1a:V543I	nsp2:V363I	-0.51473
612	Day 7	S:A701T	S:A701T	-0.77291
612	Day 7	ORF1a:T2181I	nsp3:T1363I	-0.84483
612	Day 7	S:G1246D	S:G1246D	-0.9232

Table: S2 fitness changes for each amino acid (AA) in the molnupiravir branches

Analysis of the molnupiravir mutation signature

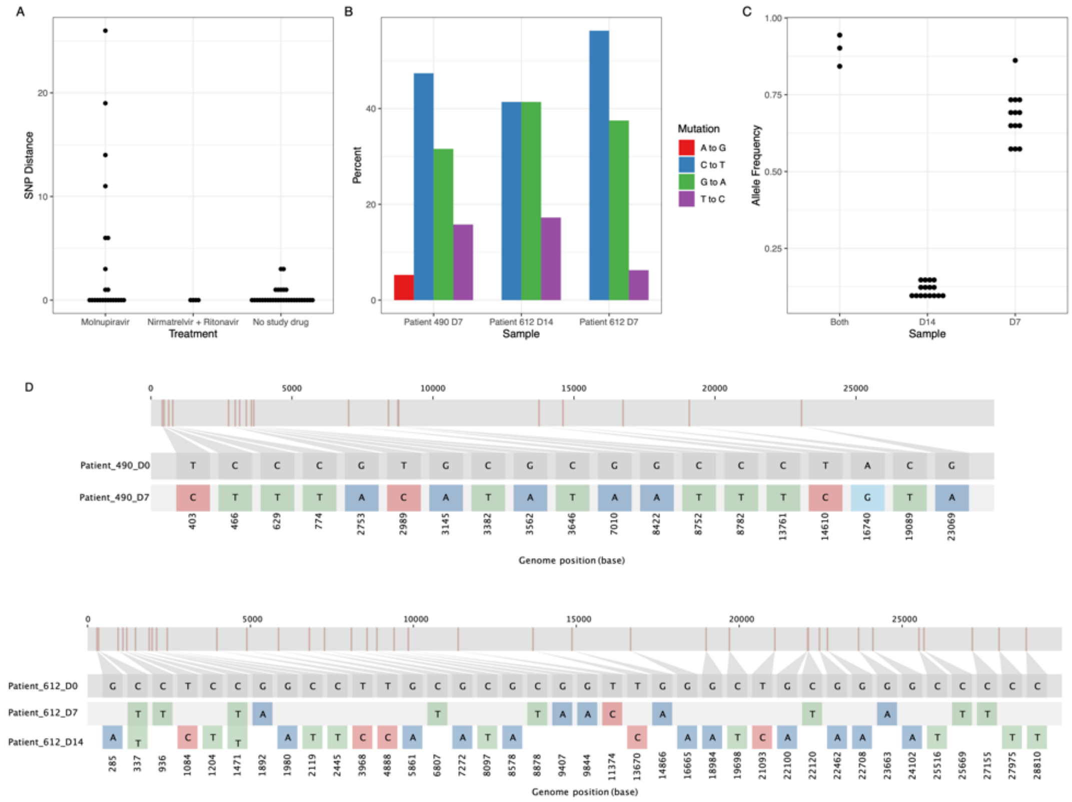

Figure S2: Analysis of the molnupiravir mutation signature. A) SNP distance between genomes sampled at day 0 and later timepoints stratified by treatment. B) Mutational spectrum of mutations gained between day 0 and later timepoints for three long branch samples. C) Mutations seen in the day 7, 10, or 14 (or more than one of these timepoints), plotted as allele frequency in the day 10 sample. D) Base and position of mutations see in the three long branch samples.

Randomisation

The randomisation sheets were generated by the trial statistician (James Watson).

All new randomisation sheets and all updates of existing randomisation sheets were done using a pre-written R script which was stored on the randomisation Dropbox® folder (owner is MORU, under custodianship of the head of MORU IT); this file is a full 'Professional' version with history recorded and only the trial statistician and head of IT had access. The file took the following inputs:

- Site codes (e.g. "th001") for which to generate randomisation sheets;
- The set of arms available for randomisation in that site;
- The number of arm repeats per block (this is set to the minimum integer such that in each block there is an integer number for each arm);
- The randomisation data file from each site (which has the patient numbers for subjects already randomised) named data-XXX.csv (where XXX is the site code), if this does not yet exist a blank .csv (headers only) is generated.

This R script is run every time a new site becomes active and every time the set of available arms changes. The output is a .csv file named rand-XXX.csv (where XXX is the site code). This overwrites the pre-existing file (which can be retrieved from the Dropbox ® version history). Each time the randomisation script is run, this is recorded on a log file.

The randomisation is done according to the following constraints:

- Blocks of 2*number of available arms;

- Additional ‘fuzziness’ by swapping one patient allocation per block at random (this can be swapped for any of the available arms) – this avoids knowing which arm the last patient per block will receive.

Each time an authorised member of the study team logs onto the web-app this is logged (timestamp and username).

Each time a new patient is randomised this is logged on to the file data-XXX.csv (where XXX is the site code) with the following information:

- Subject number
- Screening number
- Age
- Sex
- Member of study team username
- Timestamp

Statistical Analysis

The primary analysis consists of fitting Bayesian hierarchical (mixed effects) linear models to the serial log_{10} viral load data up until day 7 (i.e. viral loads on days 10 and 14 data were not used). All models encode residual error as a *t*-distribution with degrees of freedom estimated from the data. The *t*-distribution was chosen for robustness as the residual error is clearly non-Gaussian.[10,11] The *t*-distribution error model also makes the inferences robust against model mis-specification (particularly for the linear models).[12] All models include correlated individual random effect terms for both the intercept (baseline viral load) and the slope. All changes to the slope are defined as multiplicative changes on the log scale (i.e. a value of 0 equals no change).

The treatment effect is defined as the proportional change (expressed as a multiplicative term) in the population slope of the daily change in log_{10} oropharyngeal swab sample viral load. The data are modelled on the log_{10} copies per mL scale, after conversion from Ct values using the standard curve generated from the 12 control concentrations (synthetic samples with known viral densities) from each 96 well plate. The standard curve transformation is done by fitting a linear mixed effects model (random slope and random intercept for each plate) to the control data: regressing the Ct values on the known log viral densities. This borrows information across plates and adjusts for batch effects.

For all models, we adjusted the intercept and slope for the variant called (BA.5 is the reference: BA.4, BA.2.75, BQ.1 and XBB are the alternatives). A subset of models also adjusted the slopes and intercepts for:

- Age
- Days since symptom onset

- Sex

All models adjust for the number of copies of the gene encoding human RNase P (a proxy for the number of human cells in the sample). This is added as an independent linear predictor for each viral load measurement. We fitted the models using two sets of prior distributions: weakly informative priors (WIP) and non-informative priors (NIP).

The weakly informative priors were as follows:

$\alpha_0 \sim$ Normal(6, 2) (population intercept)
$\beta_0 \sim$ Normal(−0.5, 1) (population slope)
$\sigma \sim$ Normal(1.5, 3) (standard deviation of measurement error)
$\gamma \sim$ Normal(0, 1) (human RNaseP adjustment)
$\beta_{T(i)} \sim$ Normal(0, 0.5) log treatment effect
$\beta_{cov} \sim$ Normal(0, 1) (covariate effect on slope)
$\alpha_{cov} \sim$ Normal(0, 1) (covariate effect on intercept)

Random effects and hyperparameters
$\Omega \sim$ Cholesky(2) (correlation matrix for individual random effects)
$\Sigma \sim$ Exponential(1) (standard deviation for individual random effects)
$\lambda \sim$ Exponential(1) (t-distribution degrees of freedom)

For the non-informative priors we multiplied each standard deviation term by 5.
Models fitted

For each analysis we fitted 8 separate models:

1. Model 1 is linear with RNase P adjustment; adjustment for site & variant; WIP. **This is the main model used to report treatment effects.**
2. Model 2 is non-linear; RNase P adjustment; adjustment for site & variant; WIP
3. Model 3 is linear with RNase P adjustment; adjustment for site, variant, age, days since symptom onset; WIP
4. Model 4 is non-linear with RNase P adjustment; adjustment for site, variant, age, days since symptom onset; WIP

Models 5 to 8 are the same as models 1-4 but with non-informative priors (NIP).

Model 1 was used for all stopping decisions. All models have RNase P adjustment and are all combinations of linear & non-linear models, with or without full covariate adjustment; and with either weakly informative priors or non-informative priors. We compared model fits using the *loo* (approximate leave-one-out cross validation) package. The statistical analysis plan provides a detailed overview of the model structures. The non-linear model is defined as:

$$y = \log\left\{\frac{\beta_1 + \beta_2}{\beta_1 e^{-\beta_1(t-t_{max})} + \beta_2 e^{\beta_2(t-t_{max})}}\right\}$$

Where t is the time since randomisation; t_{max} is the time of peak viral density; β_1 is the slope coefficient for the increasing phase; and β_2 the slope coefficient for the decreasing phase.

Analyses

Platform trials can suffer from temporal confounding when interventions and controls are not all randomised concurrently. The primary three-way comparison between molnupiravir, ritonavir-boosted nirmatrelvir, and no study drug only used concurrently randomised patients from one single centre. The meta-analysis of all unblinded small molecule drugs did not use concurrently randomised patients only. To correct for possible temporal confounding we added the time from start of the study (scaled to have a mean of zero and standard deviation 1) for the slope and intercept terms.

Code and analysis plan

All data, models and analytical output are on the linked GitHub repository: https://github.com/jwatowatson/PLATCOV-Molnupiravir

This includes all data used in the analysis for full reproducibility of the results. The main markdown file (Molnupiravir_Analysis.qmd) runs the analysis for the primary three-way comparison. The markdown file Meta_analysis_IPD.qmd runs the meta-analysis.

Stopping rules

The stopping rules were determined using a simulation approach, based on previously modelled serial viral load data (3), such that approximately 50 patients are needed to demonstrate increases in the rate of viral clearance of ~50%, with control of both type 1 and type 2 errors at 10%. The prespecified decision criteria for stopping a treatment arm were either a model-based probability of <0.1 that the intervention did not accelerate viral clearance by >12.5% relative to no study drug (futility), or >0.9 that it did (success). In January 2023 this was increased to a 20% futility/success threshold. For the non-inferiority comparison of molnupiravir relative to ritonavir-boosted nirmatrelvir, simulations were done using a model fitted to the data from the first 500 patients recruited to the study. A 10% non-inferiority threshold was then chosen (see Statistical Analysis Plan for more details on the simulations). The updated diagram for how interventions can progress through the study is shown in Figure S3.

Figure S4 shows the estimated covariate effects on slope (left panel) and intercept (right panel) under model 3 (linear with WIP). Figure S5 shows the estimated treatment effects for all 8 models. Note a systematic change in treatment effect for the no study drug arm relative to ritonavir-boosted nirmatrelvir. This appears to be a result of model misspecification for the linear model when applied to the very rapidly clearing profiles.

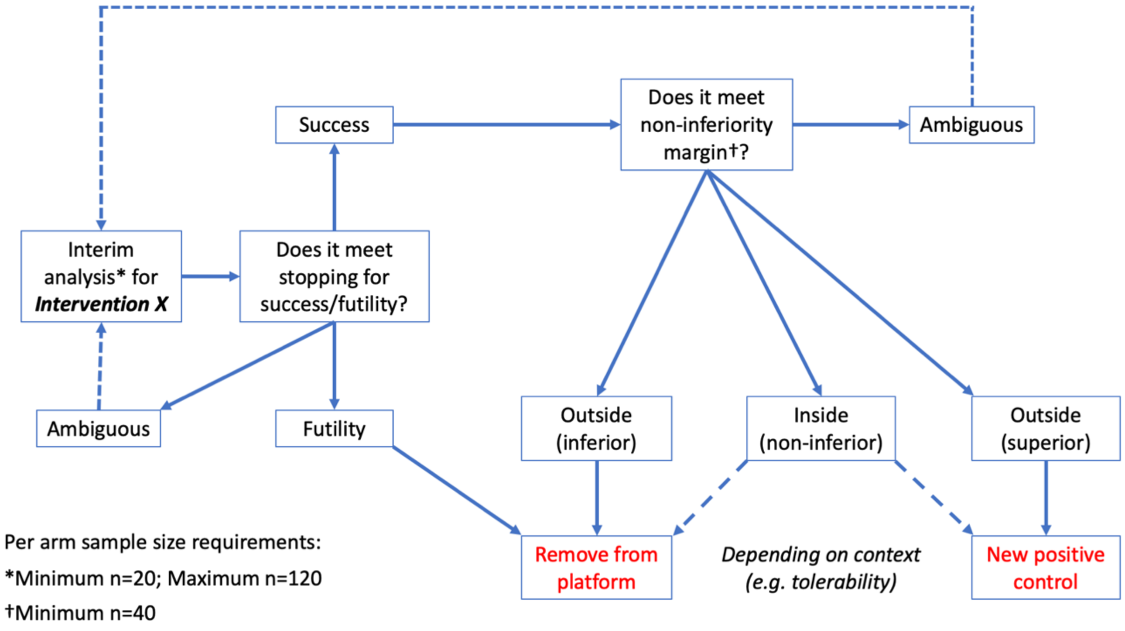

Figure S3: Schematic representation of the stopping rules as of January 2023.

Supplementary Results

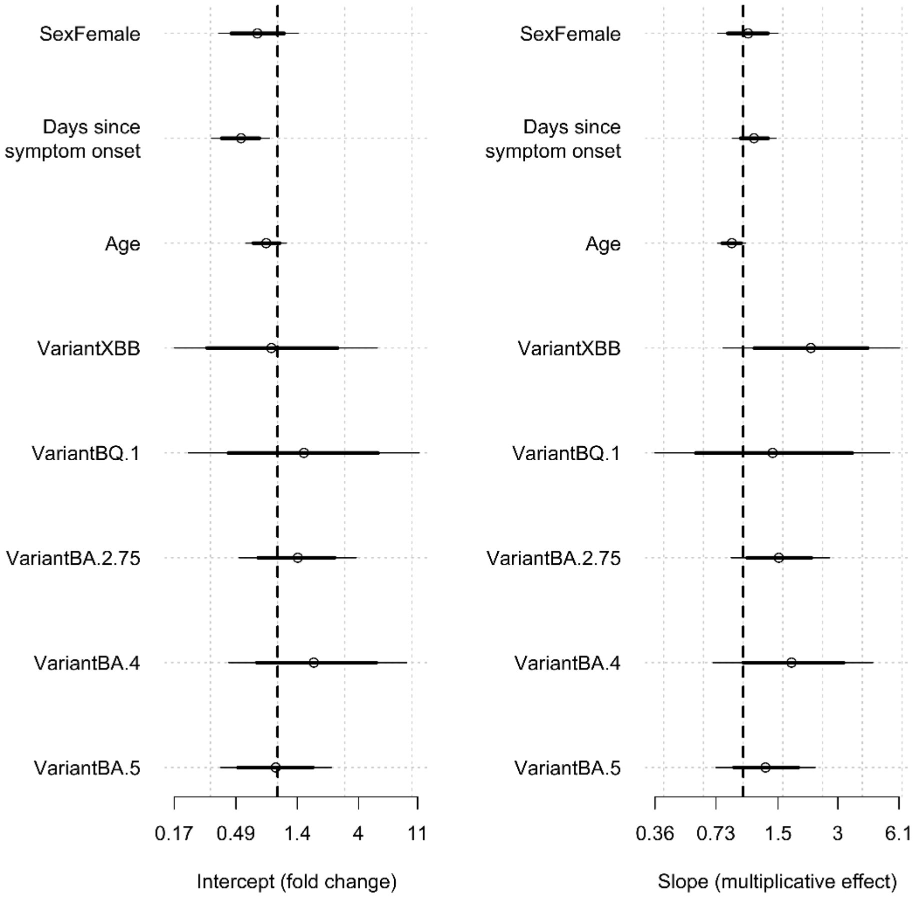

Figure S4: Covariate effects (mean estimate with 80% and 95% credible intervals shown by the thick and thin lines respectively) estimated for the main analytical linear model with full covariate adjustment (model 3, see Statistical Analysis). For the variants the reference is the BA.2 lineage. Dashed vertical line shows value of 1 (no change). Patient ages were scaled to have mean zero and standard deviation 1 so the effect size shown is for one standard deviation change in age.

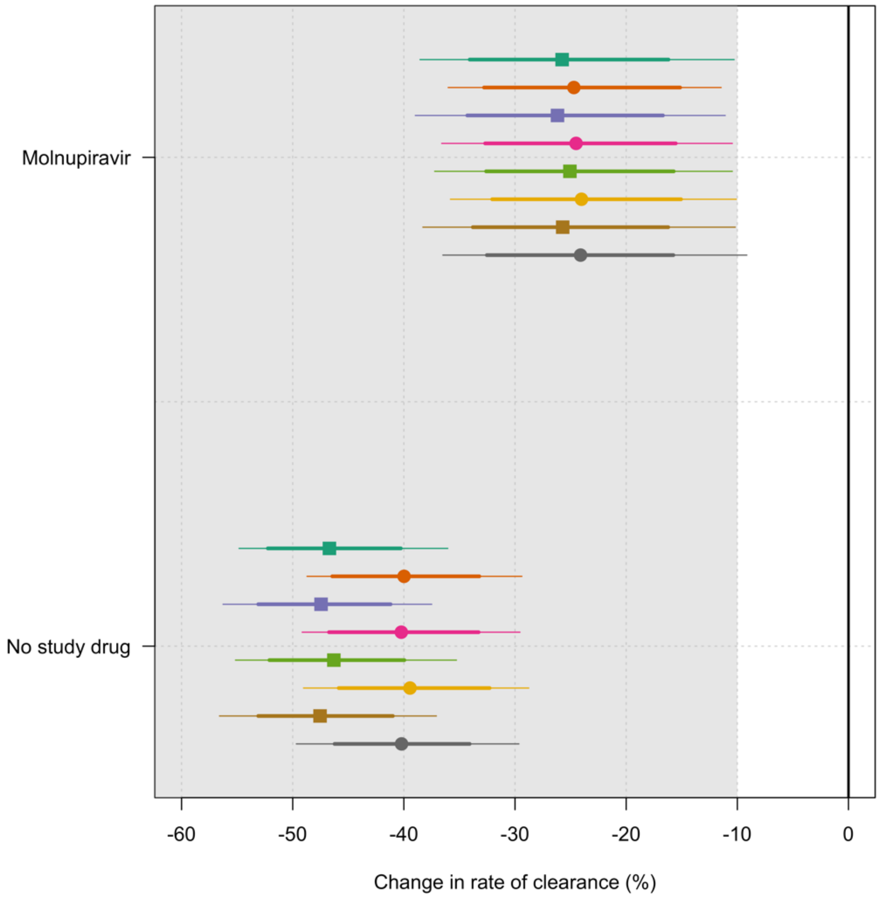

Figure S5 Nirmatrelvir-ritonavir was the most active drug tested and became the comparator in the non-inferiority comparison. Treatment effects (mean estimate with 80% and 95% credible intervals shown by the thick and thin lines respectively) for no study drug and molnupiravir relative to nirmatrelvir under the 8 analytical models (see Statistical

Analysis). Squares: linear models; circles: non-linear models. Median (IQR) follow-up duration in all treatment arms was 14 days (14-14).

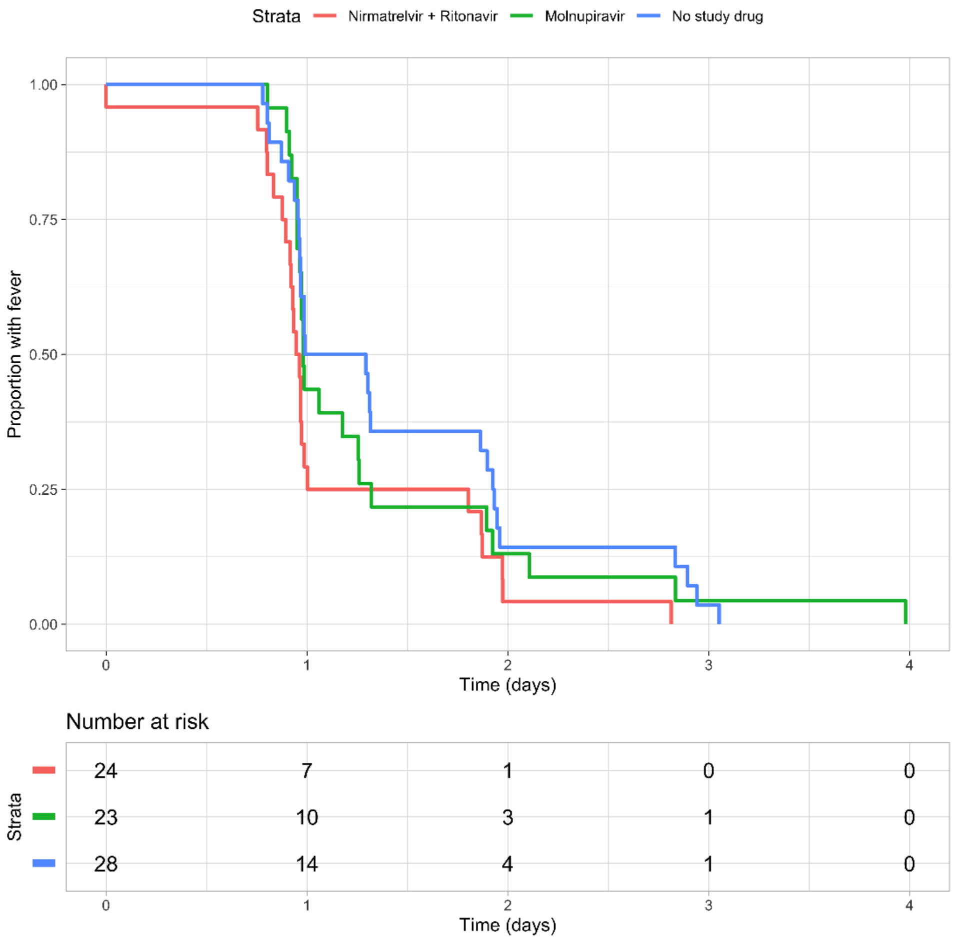

Figure S6 Fever clearance in patients who had a fever at baseline (≥37.5C). All temperatures are axillary. Fever clearance is defined as time when temperature goes ≤ 37C for at least 24 hours. There was no significant difference in survival curves between the three arms (p=0.10). Median (IQR) follow-up duration for fever in all treatment arms was 14 days (14-14). No patients were right censored (all had observed time to clearance).

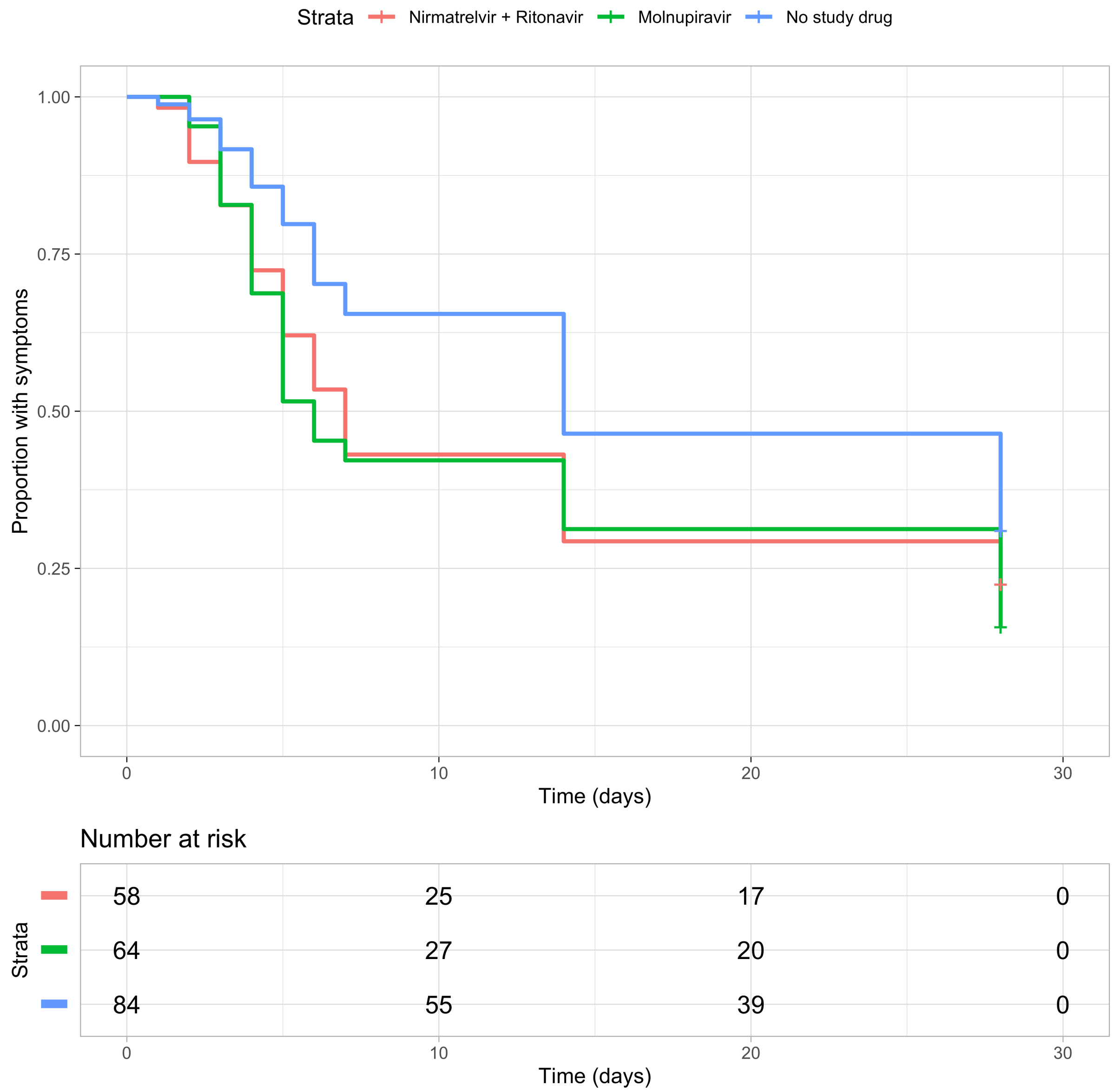

Figure S7 Symptom clearance in each arm in molnupiravir, nirmatrelvir and no study drug analysis. Symptom resolution is defined as no reported symptoms (see list of symptoms in section above). Median (IQR) follow-up duration for symptoms in all treatment arms was 28 days (28-28). 49 (23%) patients were right censored at day 28, the last scheduled visit in the study.

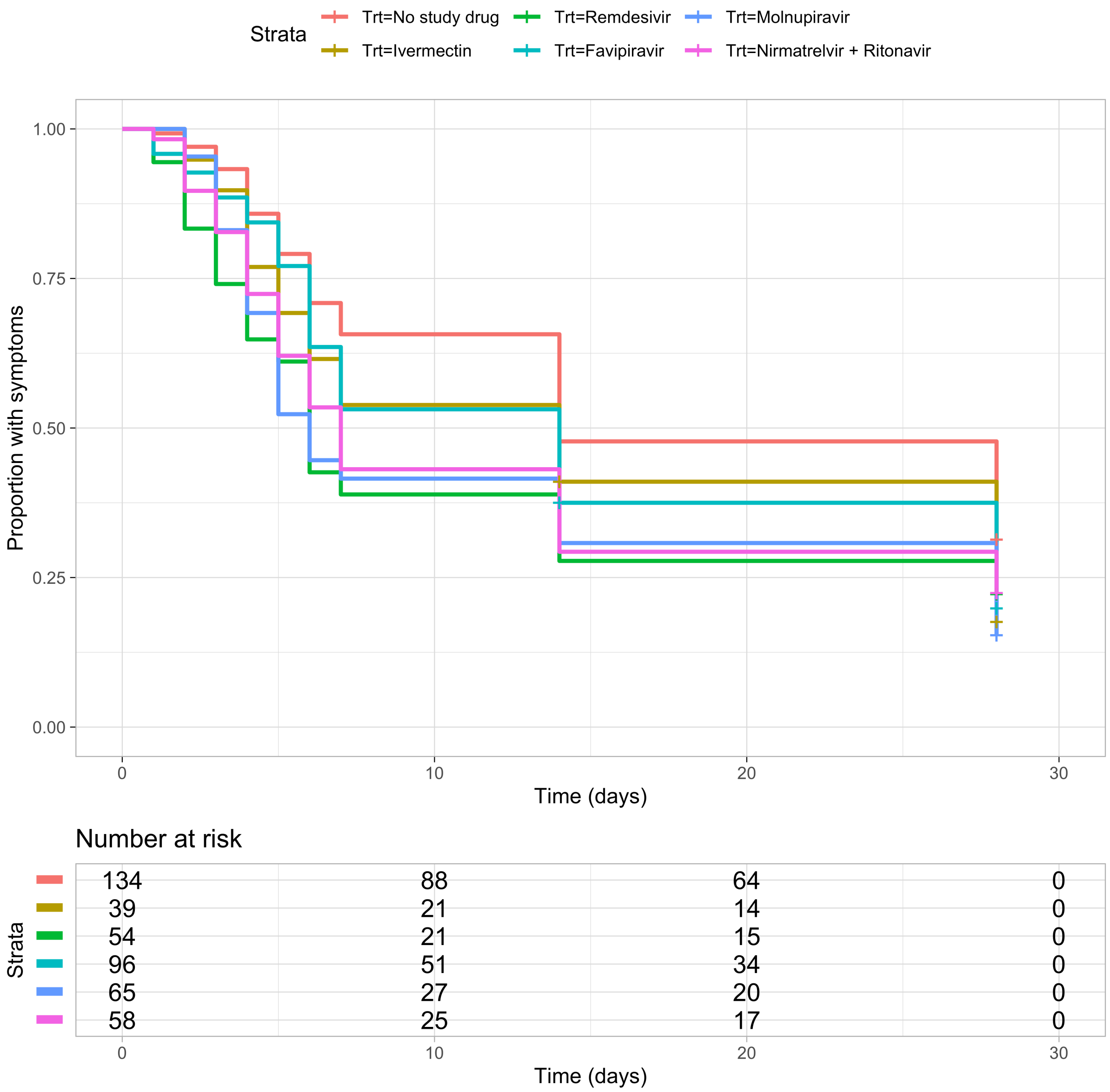

Figure S8 Symptom clearance in each arm in the individual patient data meta-analysis from this platform study. Symptoms were recorded once daily. Symptom resolution is defined as no reported symptoms (see list of symptoms in section above). Median (IQR) follow-up duration in all treatment arms was 14 days (14-14). 4 patients (1%) were right censored at 14 days (missed their day 28 visit) and 101 (23%) patients were censored at day 28 (last visit of the study).

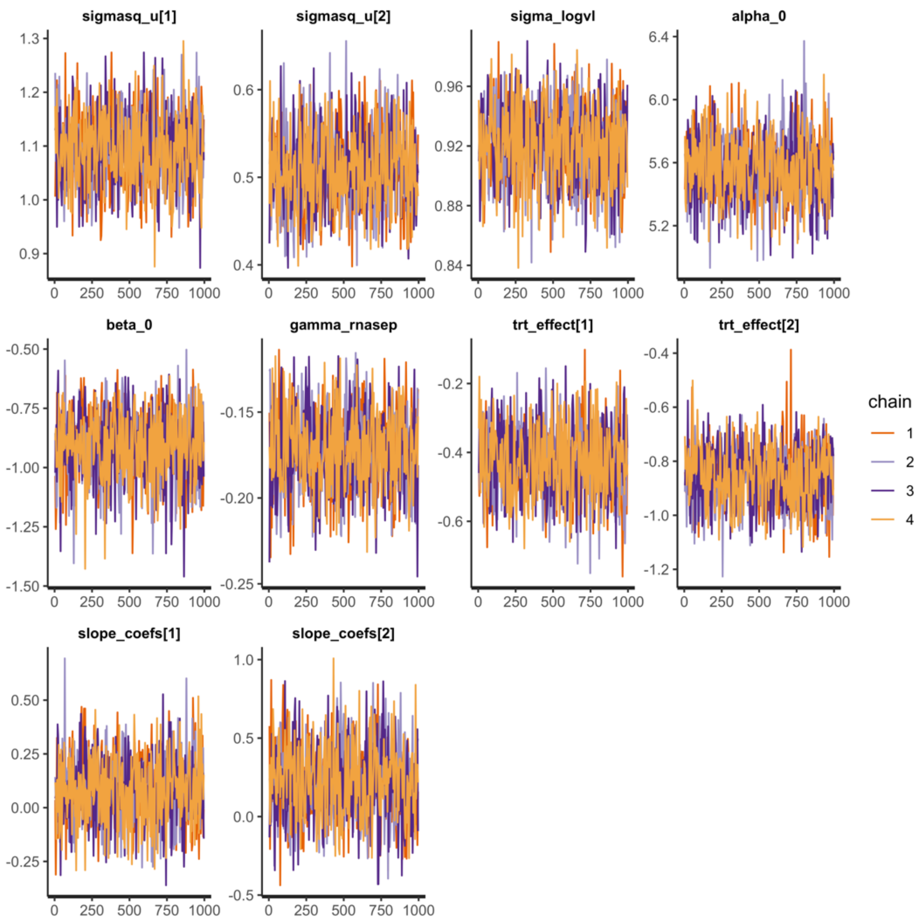

Figure S9 Traceplots for the linear model used in the primary analysis. 4 chains were run in parallel. Convergence was assessed using the Gelman-Rubin statistic.

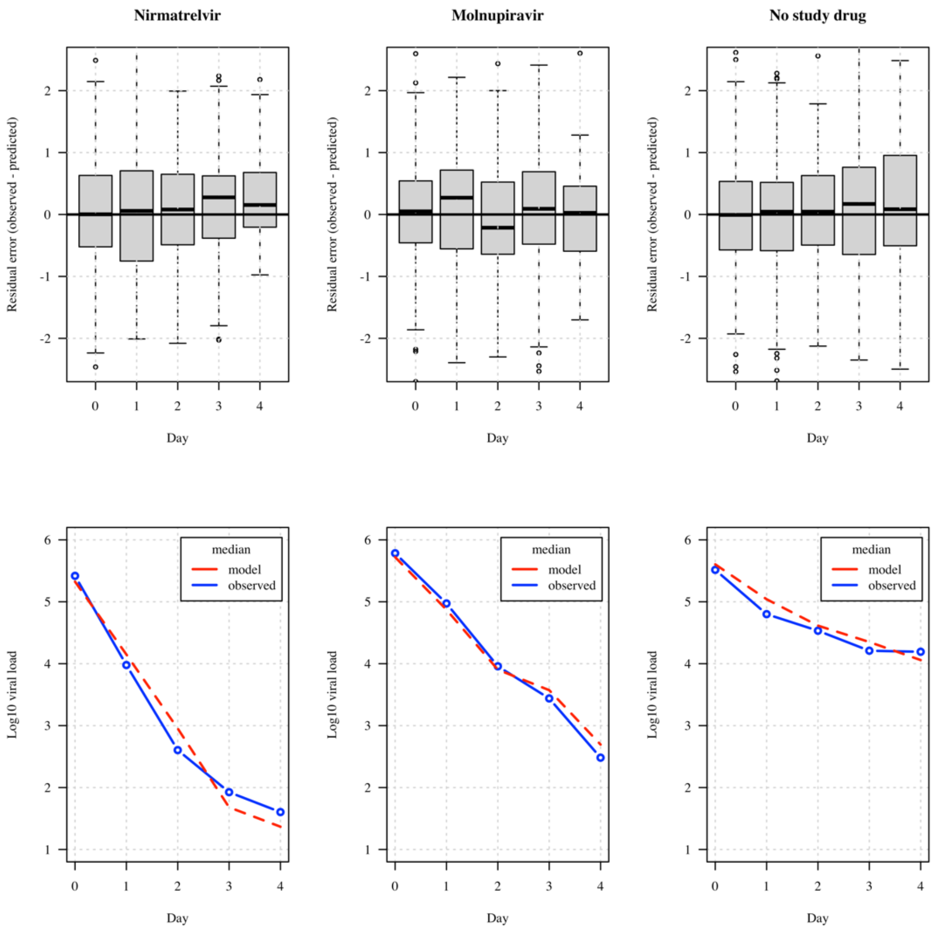

Figure S10 Analysis of the model residuals (main linear model used for the primary analysis) as a function of time from randomisation in days. The top panels show boxplots of the residuals by treatment allocation; the bottom panels show the daily median model predictions (red) against the observed daily median values (blue).

References:

1. J Q. nCoV-2019 sequencing protocol v3 (LoCost) V.3. 2020.
2. Katoh K, Misawa K, Kuma K, Miyata T. MAFFT: a novel method for rapid multiple sequence alignment based on fast Fourier transform. *Nucleic Acids Res* 2002; **30**(14): 3059-66.
3. Nextclade: clade assignment, mutation calling and quality control for viral genomes. 2021. https://www.researchgate.net/journal/The-Journal-of-Open-Source-Software-2475-9066.
4. snipit GitHub. https://github.com/aineniamh/snipit.
5. Wilm A, Aw PP, Bertrand D, et al. LoFreq: a sequence-quality aware, ultra-sensitive variant caller for uncovering cell-population heterogeneity from high-throughput sequencing datasets. *Nucleic Acids Res* 2012; **40**(22): 11189-201.
6. Bloom JD, Neher RA. Fitness effects of mutations to SARS-CoV-2 proteins. *bioRxiv* 2023: 2023.01.30.526314.
7. Interactive plots of estimated fitness effects of SARS-CoV-2 amino-acid mutations. https://jbloomlab.github.io/SARS2-mut-fitness/.
8. Index of /goldenPath/wuhCor1/UShER_SARS-CoV-2/2023/03/09. http://hgdownload.soe.ucsc.edu/goldenPath/wuhCor1/UShER_SARS-CoV-2/2023/03/09/.
9. Turakhia Y, Thornlow B, Hinrichs AS, et al. Ultrafast Sample placement on Existing tRees (UShER) enables real-time phylogenetics for the SARS-CoV-2 pandemic. *Nat Genet* 2021; **53**(6): 809-16.
10. Watson JA, Kissler SM, Day NPJ, Grad YH, White NJ. Characterizing SARS-CoV-2 Viral Clearance Kinetics to Improve the Design of Antiviral Pharmacometric Studies. *Antimicrob Agents Chemother* 2022; **66**(7): e0019222.
11. Schilling WHK, Jittamala P, Watson JA, et al. Pharmacometrics of high-dose ivermectin in early COVID-19 from an open label, randomized, controlled adaptive platform trial (PLATCOV). *Elife* 2023; **12**.
12. Lange KL LR, Taylor JMG. . Robust Statistical Modeling Using the t Distribution. *J Am Stat Assoc* 1989; **989. 84(408), 881–896**.

Master protocol:

Finding treatments for COVID-19: A phase 2 multi-centre adaptive platform trial to assess antiviral pharmacodynamics in early symptomatic COVID-19 (PLATCOV)

Short title: Finding treatments for COVID-19: A phase 2 platform trial of antiviral pharmacodynamics in early symptomatic COVID-19 (PLATCOV)

Acronym: PLATCOV

OxTREC Ref: 24-21

Trial Registration Number: NCT05041907

Protocol number: VIR21001
Date and Version No: Version 4.0 / 17 August 2022

**Co-Principal Investigators:**	**Dr William Schilling** (william@tropmedres.ac)<br>Research Physician & Infectious Diseases/ Microbiology Registrar<br>Mahidol Oxford Tropical Medicine Research Unit, Faculty of Tropical Medicine, Mahidol University, Bangkok, Thailand
	**Professor Sir Nicholas J White** (nickw@tropmedres.ac)<br>Chairman Wellcome Trust Southeast Asian Tropical Medicine Programmes & Consultant Physician<br>Mahidol Oxford Tropical Medicine Research Unit, Faculty of Tropical Medicine, Mahidol University, Bangkok, Thailand
**Co-Investigators:**	**Dr. Simon Boyd**<br>Research Physician<br>Mahidol Oxford Tropical Medicine Research Unit, Faculty of Tropical Medicine, Mahidol University, Bangkok, Thailand
	**Dr Cintia Cruz**<br>Paediatrician and Clinical Pharmacologist<br>Mahidol Oxford Tropical Medicine Research Unit, Faculty of Tropical Medicine, Mahidol University, Bangkok, Thailand
	**Dr James A Watson**<br>Statistician<br>Mahidol Oxford Tropical Medicine Research Unit, Faculty of Tropical Medicine, Mahidol University, Bangkok,Thailand
	**Dr James Callery**<br>Research Physician<br>Mahidol Oxford Tropical Medicine Research Unit, Faculty of Tropical Medicine, Mahidol University, Bangkok,Thailand

Professor Nicholas PJ Day
Director & Consultant Physician
Mahidol Oxford Tropical Medicine Research Unit, Faculty of Tropical Medicine, Mahidol University, Bangkok,Thailand

Dr Walter RJ Taylor
Senior Clinical Researcher and Infectious Diseases Physician
Mahidol Oxford Tropical Medicine Research Unit, Faculty of Tropical Medicine, Mahidol University, Bangkok,Thailand

Professor Arjen M Dondorp
Deputy Director and Head of Malaria & Critical Illness
Mahidol Oxford Tropical Medicine Research Unit, Faculty of Tropical Medicine, Mahidol University, Bangkok,Thailand

Professor Mauro Teixeira
Professor of Immunology
Unidade de Pesquisa Clínica,
Centro de Terapias Avançadas e Inovadoras
Universidade Federal de Minas Gerais,
Belo Horizonte, MG, Brasil

Assoc. Prof. Weerapong Phumratanaprapin
Dean of Faculty of Tropical Medicine, Mahidol University
Department of Clinical Tropical Medicine
Faculty of Tropical Medicine, Mahidol University, Bangkok, Thailand

Sponsor: The University of Oxford
Research Governance, Ethics and Assurance, Boundary Brook House, Churchill Drive, Oxford OX3 7GB United Kingdom

Funder: Wellcome Trust Grant ref: 223195/Z/21/Z through the COVID-19 Therapeutics Accelerator

Investigators' contributions:
NJW conceived of the study and initiated the study design with other investigators. WS, JW, CC, JC, NPJD, WRJT, and AMD supported development of protocol and implementation. JW provided statistical expertise in clinical study design.

Confidentiality Statement

This document contains confidential information that must not be disclosed to anyone other than the Sponsor, the Investigator Team, host organisation, and members of the Research Ethics Committee and Regulatory Authorities unless authorised to do so.

Investigator Agreement and Declaration of Interests

"The undersigned has read and understood the trial protocol detailed above and:

- agrees to conduct the trial in compliance with the protocol.
- agrees to comply with the principles of the International Conference on Harmonisation Tripartite Guideline on Good Clinical Practice.

and declare no conflict of interest, according to the current version of the Declaration of Helsinki"

Dr William Schilling		…17/08/2022………
Co-Principal Investigator	Signature	Date
Professor Sir Nicholas J White		…17/08/2022…………
Co-Principal Investigator	Signature	Date

Table of Contents

1. ……………… SYNOPSIS ……………… 41

2. ……………… ABBREVIATION ……………… 44

3. ……………… BACKGROUND AND RATIONALE ……………… 45

   3.1. Proposal 47

   3.2. Pharmacodynamic assessment 48

4. ……………… OBJECTIVES AND ENDPOINTS ……………… 48

5. ....STUDY DESIGN
.... 49
5.1. Interventions 50
6. ....PATIENT IDENTIFICATION AND RECRUITMENT
.... 51
6.1. Study Patients 51
6.2. Inclusion Criteria 51
6.3. Exclusion Criteria 52
7. ....STUDY SET-UP AND PROCEDURES
.... 52
7.1. Virological sampling 53
7.2. Recruitment 53
7.3. Screening and Eligibility Assessment 54
7.4. Informed Consent 54
7.5. Baseline Assessments 54
7.6. Days of study 55
7.7. Intensive pharmacokinetic sampling 56
7.8. Management of patients who become ill 56
7.9. Sample Handling and Retention 57
8. ....DISCONTINUATION/WITHDRAWAL OF PATIENTS FROM STUDY
.... 57
8.1. Definition of End of Study 58
9. .... SAFETY REPORTING
.... 58
9.1. Definitions 58
9.2. Causality 59
9.3. Procedures for Recording Adverse Events 60
9.4. Grading of laboratory abnormalities 60
9.5. Reporting Procedures for Serious Adverse Events 60
9.6. Data Safety Monitoring Board (DSMB) 61
10. ....STATISTICS AND ANALYSIS
.... 62
10.1. Overview of adaptive study design and overall approach 62
10.2. Results of the first interim analysis 63
10.3. Bayesian hierarchical model of viral clearance 63
10.4. Time varying effects 64

10.5. Sample size estimation, randomisation and statistical considerations for the Bayesian group sequential design 64
10.6. Sample size for the intensive PK-PD sub-study 65
10.7. Final analysis of primary outcome 65
11. DATA MANAGEMENT 66
11.1. Access to Data 66
11.2. Data Handling and Record Keeping 66
12. QUALITY CONTROL AND QUALITY ASSURANCE PROCEDURES 66
13. ETHICAL AND REGULATORY CONSIDERATIONS 67
13.1. Declaration of Helsinki 67
13.2. Guidelines for Good Clinical Practice 67
13.3. Approvals 67
13.4. Patient Confidentiality 67
13.5. Expenses 67
13.6. Risk 67
13.7. Benefits 68
13.8. Reporting 68
13.9. Finance and insurance 68
13.10. Data ownership 68
13.11. Publication policy 68
14. REFERENCES 70
15. Appendix 1: Schedule of activities 73
16. Appendix 2: Study drugs 75
16.1. Hydroxychloroquine 75
16.2. Remdesivir 78
16.2 Lopinavir/ritonavir 82
16.3. Miglustat 86
16.4. Ivermectin 89
16.5. Nitazoxanide 93
16.6. REGN-COV2 97
16.8 Nebulised Unfractionated Heparin (UFH) 99

16.9 Favipiravir 101
16.10 Molnupiravir 103
16.11 Nirmatrelvir/ritonavir (e.g. PAXLOVID™) 105
16.12 Sotrovimab 107
16.13 Fluoxetine 109
16.14 Fluvoxamine 112
16.15 AZD7442 (Evusheld) 114
16.16 Ensitrelvir 116
17 .... Appendix 3 – Sample size simulations 121
18 .... Appendix 4 – Exclusion criteria 124
Hydroxychloroquine 125
Remdesivir 126
Ivermectin 126
Nitazoxanide 126
Miglustat 126
Lopinavir/ritonavir 126
REGN-COV2 126
Nebulised Unfactionated Heparin (UFH) 126
Favipiravir 126
Molnupiravir 126
Nirmatrelvir/ritonavir 126
Sotrovimab 127
Fluoxetine 127
Fluvoxamine 127
AZD7442(Evusheld) 127
Ensitrelvir 127
Molnupiravir and Nirmatrelvir/Ritonavir combination 127
References for exclusion criteria 127
20. .... Appendix 5 – Amendment History 128

1. SYNOPSIS

**Study Title**	Finding treatments for COVID-19: A phase 2 multi-centre adaptive platform trial to assess antiviral pharmacodynamics in early symptomatic COVID-19 (PLATCOV)
**Protocol no.**	VIR21001
**Rationale**	Quantitative evidence of antiviral activity in patients with COVID-19 is required to justify phase 3 clinical trials of putative antivirals
**Study Design**	Randomised, open label, group sequential adaptive platform trial
**Inclusion and Exclusion Criteria**	**Inclusion criteria:**<br>• Patient understands the procedures and requirements and is willing and able to give informed consent for full participation in the study<br>• Previously healthy adults, male or female, aged 18 to 50 years at time of consent with early symptomatic COVID-19<br>• SARS-CoV-2 positive by lateral flow antigen test OR a positive PCR test for SARS-CoV-2 within the last 24hrs with a Ct value of less than 25 (all viral targets)<br>• Reported symptoms of COVID-19 (including fever, or history of fever) for less than 4 days (96 hours)<br>• Oxygen saturation ≥96% measured by pulse oximetry at time of screening<br>• Able to walk unaided and unimpeded in ADLs<br>• Agrees and is able to adhere to all study procedures, including availability and contact information for follow-up visits<br><br>**Exclusion criteria:**<br>• Taking any concomitant medications or drugs (see appendix 4)†<br>• Presence of any chronic illness/ condition requiring long term treatment, or other significant comorbidity (see appendix 4 for full list)<br>• Laboratory abnormalities discovered at screening (see appendix 4)<br>• For females: pregnancy, actively trying to become pregnant, or lactation<br>• Contraindication to taking, or known hypersensitivity reaction to any of the proposed therapeutics (see appendix 4)<br>• Currently participating in another COVID-19 therapeutic or vaccine trial<br>• Evidence of pneumonia (although imaging is NOT required)<br><br>† healthy women on the oral contraceptive pill are eligible to join the study
**Planned Sample Size**	Continuously running group sequential adaptive platform trial. There is no fixed sample size (see section 10.1 for further discussion).

	This study is expected to enroll approximately 1500 total participants from up to six countries (Thailand, Brazil, Pakistan, Laos and yet unconfirmed site/ sites).
**Planned Study Period**	3 years for total duration of the trial and 28 days for individual patients' involvement
**Interventions**	The platform trial will assess drugs with potential SARS-CoV-2 antiviral activity of three general types:<br>A. Newly available and repurposed potential antiviral drugs (see appendix 2) initially from: hydroxychloroquine, ivermectin, lopinavir/ ritonavir, fluoxetine, fluvoxamine, miglustat, remdesivir, nitazoxanide, nebulised unfractionated heparin (UFH), favipiravir, molnupiravir, nirmatrelvir/ritonavir), ensitrelvir and a combination of molnupiravir and nirmatrelvir/ritonavir.<br>B. Positive control: monoclonal antibodies initially but subsequently any therapeutic that is shown to accelerate the rate of viral clearance<br>C. Novel small molecule drugs that have gone through phase 1 testing
**Control**	No antiviral treatment (although local hospital supportive treatment will remain the same e.g. antipyretics, anti-tussives, antihistamines, vitamins etc as per the treating Physician's judgement)
**Rationale**	Each intervention type has a different objective:<br>A. Newly available and repurposed drugs are already used and recommended in some countries. Showing that they do not have significant antiviral activity is as important as showing that they do. For the newly approved antivirals, comparing antiviral activities in-vivo will inform health authorities' recommendations.<br>B. Positive control: There is good evidence from phase 2 studies that monoclonal antibodies reduce viral load in COVID-19 with some evidence that they reduce hospitalisation in high-risk individuals. However, monoclonal antibodies are vulnerable to viral escape mutations. Tracking their performance over time is important to characterise the impact and inform the therapeutics of mutant SARS-CoV-2 strains. This will also be important for other antivirals. Monoclonal antibodies are expensive and cannot be produced at large scale currently, but this may change in the near future. Not all these therapeutics may be available early in the study, and will be included if there is local availability and regulatory approval.<br>C. New small molecule drugs in development that pass phase 1 testing will have a stronger rationale (i.e. *a priori* are more likely to be effective) compared to repurposed drugs. Rapidly

	identifying the most effective candidate and the optimal dosing for phase 3 trials is the ultimate goal.	
	**Objectives**	**Endpoint**
**Primary**	A. For newly available and repurposed drugs<br>To evaluate COVID-19 antiviral efficacy in-vivo<br>B. For positive controls (e.g. monoclonal antibodies)<br>To characterise in-vivo performance over time relative to the no antiviral treatment control<br>C. For novel small molecule drugs<br>To evaluate COVID-19 antiviral efficacy in-vivo (accelerated viral clearance)	Rate of viral clearance-estimated from the $\log_{10}$ viral density derived from qPCR of standardised duplicate oropharyngeal swabs/ saliva taken daily from baseline (day 0) to day 7 for each therapeutic arm compared with the no antiviral treatment control i.e. those not receiving study drug
**Secondary**	To characterise the determinants of viral kinetics in early COVID-19 disease	Rate of viral clearance (as for primary endpoint)
	To determine optimal dosing regimens through pharmacometric assessment in antiviral drugs that are shown to be effective i.e. a positive signal (>90% probability of >12.5% acceleration in viral clearance) or evidence of antiviral effects from other studies (e.g. Molnupiravir, Nirmatrelvir/ritonavir)	Rate of viral clearance (as for primary endpoint)
	To compare viral clearance of any discovered active antiviral drugs against the positive control (e.g. REGN-COV-2 a monoclonal antibody cocktail) or other licensed and available therapeutics with evidence of accelerated viral clearance	Rate of viral clearance (as for primary endpoint)
**Tertiary**	Characterise the relationship between viral clearance and hospitalisation (hospitalisation for clinical reasons)	Hospitalisation for clinical reasons up to day 28

2. ABBREVIATION

ACT	Access to COVID-19 Tools
ADLs	Activities of daily living
AE	Adverse event
AR	Adverse reaction
ARI	Acute Respiratory Infection
COVAX	**COVID-19 Vaccines Global Access**
COVID-19	Coronavirus disease of 2019
CRF	Case Report Form
CTCAE	Common Terminology Criteria for Adverse Events
CTSG	Clinical Trials Support Group
DSMB	Data Safety Monitoring Board
EC	Ethic Committee
EDTA	Ethylenediaminetetraacetic acid
FBC	Full blood count
FDA	Food and Drug Administration
GCP	Good Clinical Practice
ICF	Informed Consent Form
ICH	International Conference on Harmonisation
IL	**Interleukin**
IM	Intramuscular
IMP	Investigational Medicinal Product
IV	Intravenous
LDH	Lactate dehydrogenase
LFT	Liver function test
MORU	Mahidol Oxford Tropical Medicine Research Unit
NGS	Next Generation Sequencing
OxTREC	Oxford Tropical Research Ethics Committee
PD	Pharmacodynamic
PI	Prinicipal investigator
PK	Pharmacokinetics
POC	Point-of-care
PPE	Personal protective equipment
RDT	Rapid diagnostic test

REGN	Regeneron
RT-qPCR	Real-time quantitative polymerase chain reaction
SAE	Serious Adverse Event
SAP	Statistical Analysis Plan
SAR	Serious Adverse Reaction
SARS-CoV2	Severe acute respiratory syndrome coronavirus 2
SC	Subcutaneous
SOP	Standard Operating Procedure
SUSAR	Suspected Unexpected Serious Adverse Reactions
TNF	Tumor necrosis factor
TMG	Trial Management Group
U&E	Urea and Electrolytes
VTM	Viral transport medium
WHO	World Health Organization

3. BACKGROUND AND RATIONALE

There are many potential therapeutics for COVID-19 and a much larger number of vaccines are in development. Vaccines are being given to individuals with the primary aim of reducing morbidity and mortality and the secondary objective of generating herd immunity in the hope of bringing the pandemic to an end. However, there is widespread vaccine inequality. As of 13 January 2022, of the 194 Member States of the WHO, 36 have vaccinated less than 10% of their population, and 88 less than 40% (1) This demonstrates how many will remain vulnerable to infection for the foreseeable future. Vaccines are the solution but there are also concerns over incomplete protection, vaccine hesitancy, waning protective effects over time, and the rapid evolution of more transmissible and potentially vaccine resistant mutant strains (highlighted by the Omicron variant (B.1.1.529), and future variants (which may not be as comparatively mild). We know that vaccine and disease-induced immunity is imperfect and short-lived so identifying active antiviral drugs is extremely important. Many people over the next 2-3 years will get COVID-19 with substantial morbidity and hundreds of thousands of deaths. There will always be people, due to their age, comorbidities or being immunocompromised that will need early antiviral therapy. For all these reasons effective therapeutics are needed urgently.

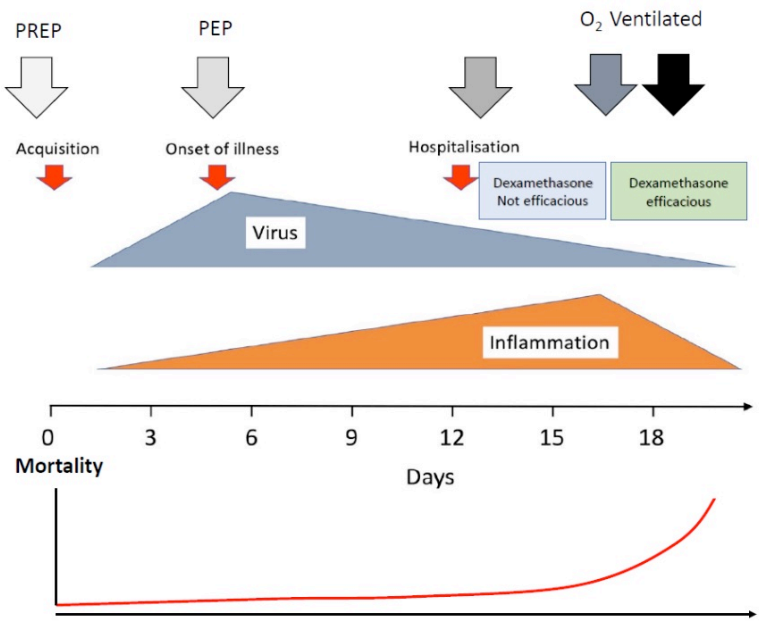

Figure 2 Simplified paradigm of COVID-19 infection and disease. There are two main stages of disease and antiviral therapies should be most effective early on.

Symptomatic COVID-19 infection has two overlapping phases (Figure 1). In the first phase, there is rapid viral replication with peak viral loads in the pharynx occurring approximately at the time of symptom onset. Thereafter viral burdens decline. In this second phase, the viral load decreases (the decline is first order, and the rate of decline is slower in sicker patients) (2-6). During the second phase, a small minority of individuals (particularly the elderly and those with co-morbidities) progress to severe illness (severe pneumonia) and some die. Hospital admission is usually about one week after the onset of illness (7). In those patients requiring respiratory support, low dose dexamethasone reduces mortality substantially (8). At this late stage of the illness inflammatory processes predominate, and antivirals are less likely to be of benefit (9). Antiviral treatments are most likely to be of benefit when given early in the illness. Accelerating viral clearance should reduce the subsequent inflammatory pathology, a hypothesis which is supported by studies of monoclonal antibody therapies and data from hospitalised patients (10, 11). At the time of writing the early protocol, there were promising monoclonal antibody therapies with evidence of viral clearance acceleration. Hospitalisation rates in RCTs were lower in the patients receiving these antibodies. However, monoclonal antibodies are currently expensive and difficult to deploy at scale (although this could change rapidly in the near future) and the continued efficacy of monoclonal antibodies is threatened by the evolution and spread of spike protein mutants (12, 13). This has been seen recently with the spread of the Omicron variant which has led, on the basis of in-vitro studies demonstrating decreased neutralization, to curtailment of their use. Two small molecule antivirals (molnupiravir, nirmatrelvir boosted by ritonavir) have completed phase 3 testing, and shown in-vivo benefits in COVID-19. They are already licensed for clinical use in some countries. However, further studies are needed, particularly comparative data on viral clearance between these two drugs, and better characterization of the phenomenon of 'viral rebound', where there is a recrudescence of active infection which has implications for transmission. In-vivo synergy testing of combinations of antivirals will also be important to assess the effects on viral rebound, to prevent development of mutations during treatment (i.e. combination therapies given in HIV), for the severely immunocompromised and if more

severe variants appear. More antivirals will likely follow, and comparisons of their antiviral effects will help healthcare systems decide on their purchase and use.

There is no optimised or validated approach to assess rapidly potential antiviral therapeutics in COVID-19. Drugs are currently being selected for clinical study on the basis of activity in cell culture systems (in-vitro) and animal models in-vivo. Unfortunately the animal models are not sufficiently good to be included in the drug development critical pathway. Some manufacturers (e.g. Pfizer) have decided not to try and assess antiviral activity of candidate drugs in these animal models. Thus drugs currently under consideration are justified only on the basis of antiviral activity in cell cultures. In order to identify effective antivirals and optimise their dosing, and therefore ensure that phase 3 studies are designed appropriately, and progress is as rapid as possible, in-vivo antiviral effects must be characterized adequately. This can be achieved in natural COVID-19 infections at an early stage of the disease using the following design. The proposed trial will develop and validate a platform for quantitative assessment of antiviral effects (5) in low-risk patients with high viral burdens and uncomplicated COVID-19. As the assessment is of the antiviral effect on the virus, as distinct from the outcome on the clinical course of the infection, the results are applicable to all stages of disease where viral replication occurs (including prophylaxis).

3.1. Proposal

In this randomised open label, controlled, group sequential adaptive platform trial, we will assess the performance of three distinct types of intervention relative to control (no treatment):

A: Newly available and repurposed antiviral drugs; and if available;

B: "Positive control" (e.g. monoclonal antibodies); and later;

C: Small molecule drugs that pass phase 1 testing.

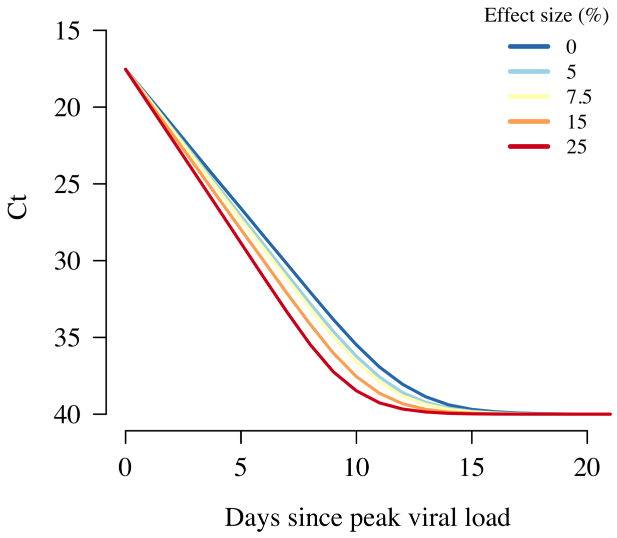

Figure 3. Acceleration of viral clearance rates by different amounts

The trial initially used repurposed drugs as they were available at the time, and some are used widely without good evidence of benefit. Subsequently newly developed small molecule antiviral drugs have become available (e.g. Molnupiravir and Nirmatrelvir/ritonavir). For these newly available and repurposed drugs under consideration (to be defined as type A interventions), the platform trial is designed based on the premise that showing clear evidence of an antiviral effect is as important for medicine and public health as proving there is no significant effect (i.e. Type A adaptive design best suits the trial aims here). Antivirals shown to be beneficial in the study may subsequently be chosen to be Type B interventions (e.g. "Positive controls"). Proving that a drug does not accelerate viral clearance in-vivo should provide evidence to stop clinical recommendations based on claims of putative antiviral activity in ex-vivo cell based assays. Monoclonal antibodies (to be defined as type B interventions) were initially included as a "positive control". This is beneficial to demonstrate that the method does identify active compounds. It will be important to calibrate and

compare the effects of these validated effective therapies relative to the newly identified small molecule antiviral drugs. In addition, for monoclonal antibodies it is also important to characterise waning efficacy over time resulting from novel spike protein mutants (12,13)
We aim to identify the best candidates rapidly of the novel small molecule drugs. The distinct objectives for each of these intervention types requires different stopping rules and design features. The rapid turn-round in quantitative PCR measurements is key to the success of this project. Analysis of these results will allow for frequent interim analyses with the aim of halting study arms early for futility, or triggering an intensive nested PK-PD study if there is evidence of clinically significant antiviral activity, although early interim analyses are not likely to yield efficacy assessments, and are aimed primarily at validation of the methodology. This will speed up the identification of antivirals to be assessed in phase III studies. The key metric is the rate of viral clearance (5) (Figure 2).

3.2. Pharmacodynamic assessment

We have used prospectively collected serial viral load data from 46 individuals infected with SARS-CoV-2 and an accompanying pharmacodynamic model to simulate different trial designs for putative antiviral drugs using either time-to-clearance or rate-of-clearance as the primary trial endpoint (15). This model was then tested on data from a small pilot study of 24 patients with early symptomatic COVID-19 who were randomised to either ivermectin or placebo (16). **Rate-of-clearance was shown to be a uniformly better endpoint in terms of type 2 error (i.e. results in increased power) than the widely used and reported time-to-clearance (4).** Rate of decline in oropharyngeal qPCR estimates of viral density measured daily also allows shorter and more efficient evaluation of viral clearance (Figure 3). Our simulations (Appendix 3) show that the proposed trial design can identify an effective therapy rapidly in the setting of a platform trial and quickly discard ineffective therapies (both of which are the primary objectives of the trial).

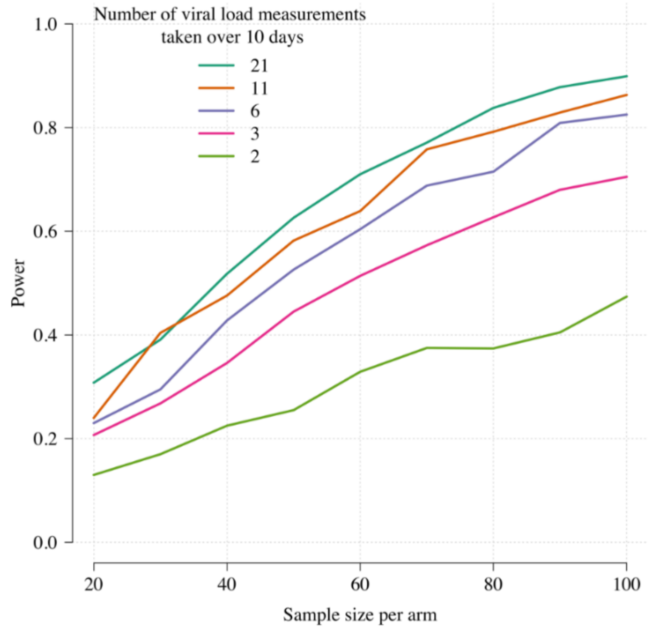

Figure 3. The relationship between the number of qPCR viral load measurements taken over 10 days and the power (1-type 2 error) to detect an effect size of 10% increase in viral clearance rate relative to control. This assumes that patients are recruited at peak viral load (4).

4. OBJECTIVES AND ENDPOINTS

	**Objectives**	**Endpoint**	**Timepoint(s) of evaluation of this endpoint (if applicable)**

**Primary**	A. **<u>For newly available and repurposed drugs</u>**<br>**To evaluate COVID-19 antiviral efficacy in-vivo**<br>B. <u>For positive controls (e.g. monoclonal antibodies)</u><br>To characterise in-vivo performance (over time) relative to the no antiviral treatment control<br>C. <u>For novel small molecule drugs</u><br>To evaluate COVID-19 antiviral efficacy in-vivo (accelerated viral clearance)	Rate of viral clearance (estimated from the log_{10} viral density derived from qPCR of standardised duplicate oropharyngeal swabs/ saliva taken daily from baseline (day 0) to day 7 for each therapeutic arm compared with the no antiviral treatment control i.e. patients not receiving study drug)	Days 0-7
**Secondary**	To characterise the determinants of viral kinetics in early COVID-19 disease.	Rate of viral clearance	Days 0-7
	To determine optimal dosing regimens through pharmacometric assessment in antiviral drugs that are shown to be effective i.e. show a positive signal (>90% probability of >12.5% acceleration in viral clearance) or evidence of antiviral effects from other studies (e.g. Molnupiravir, Nirmatrelvir/ritonavir)	Rate of viral clearance	Days 0-7
	To compare viral clearance of any discovered active antiviral drugs against the positive control (e.g. REGN-COV2 (monoclonal antibody cocktail) or other licensed and available therapeutics with evidence of accelerated viral clearance	Rate of viral clearance	Days 0-7
**Tertiary**	Characterise the relationship between viral clearance and hospitalisation (hospitalisation for clinical reasons)	Hospitalisation for clinical reasons up to day 28	Days 0-28

5. STUDY DESIGN

The study is a randomised, open label, controlled adaptive platform trial that will be conducted in low-risk patients with COVID-19, recruited from outpatient COVID-19/ Acute Respiratory Infection (ARI) clinics or through other approved facilities, or from inpatient isolation facilities, or by patient self-referral to the study site, that is previously healthy patients 18 to 50 years old with early symptomatic COVID-19 and without co-morbidities (see appendix 4). The study has started in Thailand and Brazil.

After obtaining fully informed consent, we will recruit adult patients with early symptomatic COVID-19 (less than 4 days (96 hours) since the reported onset of symptoms), who are

positive by a SARS-CoV-2 lateral flow antigen test (identifying those with higher starting viral densities) OR a positive PCR test for SARS-CoV-2 within the last 24hrs with a Ct value of less than 25 (all viral targets). A SARS-CoV-2 antibody RDT will be done and the result recorded (antibodies strongly correlate with higher viral loads (11)). Patients will be included who can be followed up reliably for 28 days (17-19). It is expected that vaccination coverage will increase during the study period as well as the cumulative number of natural infections.

The primary pharmacodynamic measure in this study is the rate of viral clearance (expressed as the slope of a fitted regression to the linear segment of the serial standardized oropharyngeal sample qPCR densities from day 0 to day 7) following treatment (5). Each site will include a no antiviral treatment control arm consisting of patients not receiving any antiviral treatment, although local hospital supportive treatment will remain unchanged for the patients including antipyretics, anti-tussives, antihistamines, vitamins etc and other required medications, in the clinical judgement of the treating Physician.

5.1. Interventions

Intervention arms will be of three types:

A. Newly available and repurposed antiviral drugs. The initial list of intervention drugs is Hydroxychloroquine, Ivermectin, Lopinavir/Ritonavir, Fluoxetine, Fluvoxamine, Miglustat, Remdesivir, Nitazoxanide, Nebulised Unfractionated Heparin (UFH), Favipiravir, Molnupiravir and Nirmatrelvir/Ritonavir, Ensitrelvir and a combination of Molnupiravir and Nirmatrelvir/Ritonavir. Additional repurposed drugs can be added to the list. The interventions will be chosen in order of priority, as well as local feasibility at sites (availability of drugs, local EC and regulatory approvals).
B. Positive control: we will initially include the REGN-COV2 (monoclonal antibody cocktail); and later other antibodies when available, including Evusheld. Antivirals shown to be beneficial in the study may subsequently be chosen to be Type B interventions (e.g. “Positive controls”)
C. Novel small molecule drugs: there are no candidates initially, but these can be added to the platform at any time after demonstrating success in phase 1 clinical trials.

At any given time in the study, it is possible that not all intervention arms are available. Randomisation ratios will be uniform across all available treatment arms within type A, and type B and will be at least 20% in the control arm in each site. If interventions of type C (novel drugs) are added to the platform, response adaptive randomisation will apply.
Randomisation will be continuously monitored by MORU. A positive SARS-CoV-2 lateral flow antigen test will be used as the main inclusion criteria. A positive lateral flow test result implies a relatively high viral load at the start of the study intervention (17-19). In order to assess viral clearance dynamics accurately over time initial high viral densities are required. In non-vaccinated patients, seronegativity implies there will be limited host control of the infection initially and was previously strongly correlated with higher viral loads (15), although this also depends on the variant.

We aim to identify interventions that accelerate viral clearance by a minimum of 12.5% (corresponding approximately to a reduction of 2 days in the mean time to viral clearance). Any intervention drug of type A or C that shows an acceleration of viral clearance, defined as

a greater than 90% probability of accelerating viral clearance by more than 12.5%, or if there is evidence of accelerated viral clearance from other studies (e.g. Molnupiravir, Nirmatrelvir/ritonavir), will be selected for inclusion in a nested pharmacokinetic-pharmacodynamic study. In this nested study, frequent blood sampling will allow assessment of the relationships between plasma drug concentrations and viral clearance. The design of the pharmacokinetic study depends on the known pharmacokinetic properties of each drug. In each case no more than 10 blood samples (20mL) will be taken. Any intervention arm of type A, B or C that meets the futility endpoint (less than 10% probability of accelerating viral clearance by more than 12.5%) will be dropped from the study. New arms can be added at any time during the trial. Decisions concerning success and futility will be made sequentially at the pre-specified interim analysis to be conducted after 50 patients are enrolled, and thereafter after every 25 patients to ensure control of type 1 and type 2 error (Figure 4). The nested intensive PK-PD substudy will be triggered if the probability that viral clearance is accelerated by more than 12.5% goes above 90%, or if there is evidence of accelerated viral clearance from other studies (e.g. Molnupiravir, Nirmatrelvir/ritonavir).

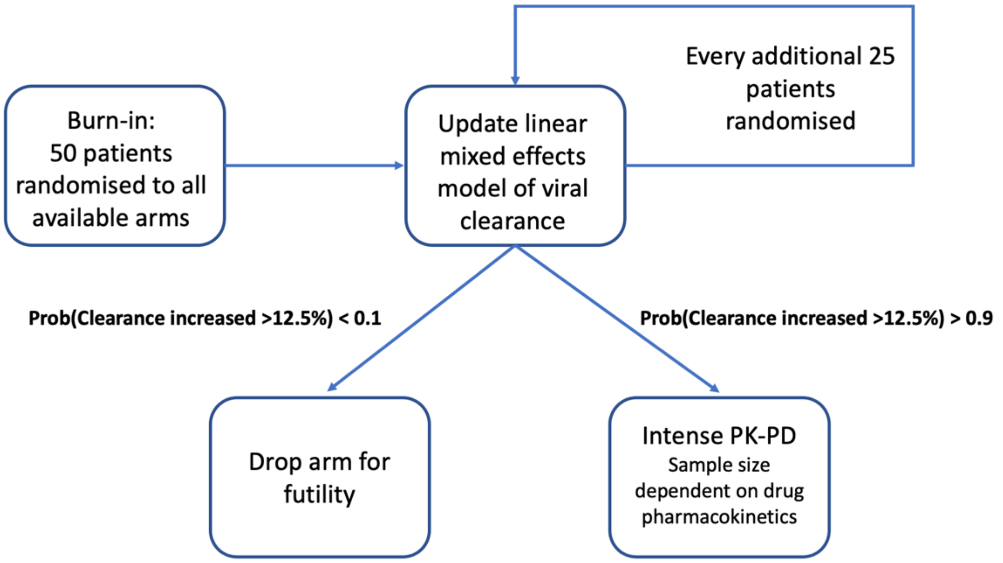

Figure 4. Flow diagram showing how each intervention arm can progress through the study. An interim analysis was conducted after qPCR data from the first 50 patients recruited into the study were available (burn-in). Ongoing internal monitoring will occur for every additional batch of 25 studied patients. Following the first interim analysis, the stopping rule threshold effect size was changed from 5% to 12.5 based on the observed effect of Regeneron and on sample predictions using observed uncertainty intervals. Futility occurs if the probability of success (increase in viral clearance > 12.5% relative to control) is less than 10%. Success occurs if the probability of success is greater than 90%, triggering an intensive pharmacokinetic-pharmacodynamic substudy for that arm.

6. PATIENT IDENTIFICATION AND RECRUITMENT

6.1. Study Patients

Previously healthy adult patients with early symptomatic COVID-19.

6.2. Inclusion Criteria

- Patient understands the procedures and requirements and is willing and able to give informed consent for full participation in the study.

- Previously healthy adults, male or female, aged 18 to 50 years at time of consent with early symptomatic COVID-19
- SARS-CoV-2 positive by lateral flow antigen test OR a positive PCR test for SARS-CoV-2 within the last 24hrs with a Ct value of less than 25 (all viral targets)
- Symptoms of COVID-19 (including fever, or history of fever) for less than 4 days (96 hours).
- Oxygen saturation ≥96% measured by pulse-oximetry at time of screening.
- Able to walk unaided and unimpeded in ADLs
- Agrees and is able to adhere to all study procedures, including availability and contact information for follow-up visits

6.3. Exclusion Criteria

The patient may not enter the study if ANY of the following apply:

- Taking any concomitant medications or drugs (see appendix 4)†
- Presence of any chronic illness/ condition requiring long term treatment, or other significant comorbidity (see appendix 4 for the full list)
- Laboratory abnormalities discovered at screening (see appendix 4)
- For females: pregnancy, actively trying to become pregnant, or lactation
- Contraindication to taking, or known hypersensitivity reaction to any of the proposed therapeutics (see appendix 4)
- Currently participating in another COVID-19 therapeutic or vaccine trial
- Evidence of pneumonia (although imaging is NOT required)

† healthy women on the oral contraceptive pill are eligible to join the study

7. STUDY SET-UP AND PROCEDURES

Recruitment of patients will be from outpatient COVID-19/ ARI clinics or through other approved facilities, or from inpatient isolation facilities, or by patient self-referral to the study site. Patients with a positive SARS-CoV-2 rapid lateral flow antigen test OR a positive PCR test for SARS-CoV-2 within the last 24hrs with a Ct value of less than 25 (all targets) will be enrolled in the study after providing fully informed written consent. In each study site there will be a designated research ward for study procedures. In accordance with local and national infection control guidelines the initial assessments will be conducted in a designated research ward set-up to manage COVID-19 patients. Patients will receive care in either an inpatient or outpatient setting, in accordance with local and national guidelines. All recruited patients will be visited at their home or place of residence, or be seen in the designated clinical trials unit or hospital ward. All staff will wear appropriate personal protective equipment (PPE). For home visits a trained member of staff will perform the swabbing (and may ask the patient to give a saliva sample).

The rapid lateral flow antigen test will be performed by trained study personnel. Patients will be eligible for the trial if the test is positive within a specified period of time (detailed in a separate SOP based upon manufacturer's guidelines). A positive band appearing rapidly is correlated with a higher viral load in the swab sample (personal communication, Prof. Tim Peto). A digital photograph of the test result may be taken to allow for future analysis. The exact details of this will be detailed in the separate work instruction.

Participants will be asked if they have been in the study before and whether any relatives have been in the study. After enrolment into the study, the patient will be randomised to one of the study arms. Further details of the randomisation procedures are given below. An

oropharyngeal swab will be obtained in duplicate by the study team using a standard operating procedure (SOP) after a full explanation is provided to the patient. Swabs will be taken in duplicate again prior to receiving the study drug. To minimise patient discomfort, all SARS-CoV-2 qPCRs will be performed on eluates from standard oropharyngeal swabs, taken in duplicate (20-23). In addition, some patients may be asked to give a sample of saliva (see section 7.1). The patient will undergo a physical examination and blood sampling for routine haematology and biochemistry and other baseline investigations (see Section 7.5). For oral study drugs, the first dose will be administered and observed at the research ward/ hospital. Subsequent doses will be administered by the ward staff in hospital if the patient is an inpatient, at the patient's home, or current residence, supervised by study personnel (if multiple doses, then one of the doses per day will be observed). In case a study drug requires parenteral administration, this will be provided under supervision by the study personnel in the research ward, or hospital, or the patient's home.

7.1. Virological sampling

Serial oropharyngeal swabs will be taken in duplicate (one swab each tonsil) (24) according to the sample schedule. The swabs will be taken from the back of the throat (oropharynx; tonsillar fossa) by trained study personnel according to the SOP abiding by strict PPE measures. Each swab will be placed in 3mL of viral transport medium (VTM) (Thermofisher M4RT®), snapped off and sealed. In addition, some patients may be asked to give a sample of saliva, either by spitting in a tube, or by sucking on a swab tip until it is saturated. The volume of saliva will be standardized. The initial purpose of this is to assess and validate a method of determining the viral density in the oropharynx in a less invasive manner or supplement the results from the oropharyngeal swabs to improve the characterization of the viral clearance. If these results are informative, they may subsequently be included in the SAP for endpoint analysis. Daily sampling will be performed by the study personnel who will place the swabs in the transport medium, and document the time. The samples will be kept cool, and subsequently stored at -80°C as detailed in the SOP. Transport times will be recorded, as well as the time at which the swab is frozen at -80°C. Viral genomes from the throat swabs will be quantitated by RT-qPCR, according to published methods (25). Each observed value will be recorded along with the RNAseP $\log_{10}$ density (representing human cell numbers). The exact details of the analysis are given in the SAP (26,27). The measurements for the patient over time will be used to estimate the rate of viral clearance under a Bayesian hierarchical linear mixed effects model. The concentration of urea in the VTM will also be measured and compared with the serum urea concentration in the baseline biochemistry sample. This comparison enables determination of the extracellular fluid content in the eluate volume of the oropharyngeal samples. The urea concentration in throat fluid is the same as in serum, so that the dilution factor of the throat sample in the VTM fluid can be easily calculated (28). Blood will also be taken for quantitative virology and other baseline assessments (see Section 7.5). All samples positive for SARS-CoV-2 on qPCR will be sequenced using Next Generation Sequencing (NGS), or canonical variant mutation analysis will be used to determine the virus genotype, in order to determine the effect of viral genetics on viral load and response to treatment.

7.2. Recruitment

Potential patients with a positive SARS-CoV-2 test obtained during routine screening, or those with symptoms in keeping with COVID-19, even if they do not have the results of their SARS-CoV-2 test, or have not been tested yet, may be contacted by the research group.

Alternatively they may contact the research group for participation in the study. Each will be provided with a written patient information sheet.

7.3. Screening and Eligibility Assessment

Eligibility assessment will occur at the point of screening. If, based on the inclusion and exclusion criteria, the patient is eligible and willing to complete the full study, they will be included provided informed consent is obtained.

7.4. Informed Consent

Written and verbal versions of the Patient Information and Informed Consent will be presented to the patients detailing the exact nature of the study; what it will involve for the patient; the implications and constraints of the protocol; and the known side effects of the medicines under evaluation and any risks involved in taking part. It will be stated clearly that the patient is free to withdraw from the study at any time for any reason without prejudice to future care, and with no obligation to give the reason for withdrawal.

The patient will be allowed as much time as required to consider the information as long as they remain eligible and within the time-frame for recruitment into the study, and the opportunity to question the Investigator or other independent parties to decide whether they will participate. Written Informed Consent will then be documented by a patient dated signature and dated signature of the person who presented and obtained the Informed Consent. The person who obtained the consent must be suitably qualified and experienced and have been authorised to do so by the site PI. A copy of the signed Informed Consent will be given to the patient. The original signed form will be retained at the study site.

7.5. Baseline Assessments

A physical exam, vital signs, symptom review and basic demographics will be recorded. Oropharyngeal viral swabs taken by the study personnel in duplicate (swabs will be taken at two timepoints prior to study drug being administered) (and possibly a saliva sample see section 7.1). A total of 20 ml of venous blood for study testing will be taken at this point. Administration of the therapeutic may be delayed until the results of the FBC and Biochemistry will be available (although these will ideally be available within 1 hour of sample collection i.e. before randomization), to determine if there is any contraindication. If these bloods are not available in this timeframe, then the patient can be safely enrolled, randomized, and given their first dose, and can be excluded prior to their next dose if laboratory abnormalities resulting in a contraindication are present. If an FBC and Biochemistry has been taken in the previous 24hrs, the results of these can be used to determine whether there is a contraindication.

Laboratory tests at baseline

- Blood sample for
  - Full blood count (FBC) and differential count
  - Biochemistry (including U&Es, creatinine, LFTs and LDH)
  - Fibrinogen, Ferritin, TNF/IL-6/IL-1
  - Serology (antibody responses)
  - Stored sample
  - Drug level
  - Host genotyping to assess determinants of the therapeutic response

- Oropharyngeal swab in duplicate (and possibly a saliva sample see section 7.1)
- ECG

7.6. Days of study

7.6.1 Randomisation D0

After enrolment, the patient will be randomized to one of the study arms using an online randomization application. The number of arms at a site can be limited by the approval and availability of the study drug at that site. Randomization to the no antiviral treatment control arm (no intervention) will be fixed at a minimum of 20% throughout the study. The randomization ratios will be uniform for all available interventions. The drug allocations are not blinded for the patient for practical reasons. For patients randomized to an active drug, the first treatment dose (hour 0) will be supervised by study team after a viral swab (and possibly a saliva sample see section 7.1) is taken again for qPCR (taken in duplicate) (see appendix 1 for schedule of activities). The patient will then be observed for a minimum of 1 hour in the ward.

For the therapeutic arms for which intensive PK has been triggered, allocated patients will stay for extra blood tests for drug monitoring levels, according to the schedule in the drug-specific appendix. For further details of the intensive PK schedules for the individual therapeutics please see the appendix 2.

7.6.2 Day 1- day 7

The following variables will be assessed daily:

- Eligibility check, and assessment of treatment (taking of medications taken will be observed by the study personnel and the time recorded. In participants who are outpatients, for medications taken more than once daily, the patient will record the time non-observed doses are taken).
- Oropharyngeal swab for qPCR taken in duplicate (and possibly a saliva sample see section 7.1) by the study personnel (time taken recorded by the study personnel)
- Temperature recorded twice daily by study staff or participant (information collected in a diary and reviewed by the study personnel daily)
- Study personnel completes a brief symptom questionnaire each day
- Two blood samples will be taken (ideally day 3 and 7) for antibody levels and for later pharmacokinetic analyses (pre- or post-dose depending on the therapeutic in question) and FBC and biochemistry, to assess potential drug adverse effects.

7.6.3 Day 10

All patients will have oropharyngeal swabs taken in duplicate (and possibly a saliva sample see section 7.1) either at home (if feasible) or at the research ward. These extra swabs are to better capture viral rebound should this occur, as has been described with certain antivirals.

7.6.4 Day 14

All patients will have oropharyngeal swabs taken in duplicate and a blood test taken on day 14 (and possibly a saliva sample see section 7.1) either at home (if feasible) or at the research ward to follow-up blood abnormalities, if identified in the earlier tests, and to determine antibody responses (serology).

7.6.5 Day 28 assessment

The day 28 follow-up assessment will be through a telephone call or a visit to check whether the patient recovered uneventfully for whether there was progression to more severe illness or need for hospitalization for clinical reasons. This final assessment will have an allowed window -2/ +7 days based upon calculation from Day 0.

7.7. Intensive pharmacokinetic sampling

For those study drugs that are shown to be effective antivirals in other studies (e.g. Molnupiravir, Nirmatrelvir/ritonavir) or meet the success criteria of the study (defined as a greater than 90% probability of accelerating viral clearance by more than 12.5%), we will then conduct intensive pharmacokinetic (PK) sampling for further patients who are enrolled into that arm. The exact sampling schedule will depend on the study drug characteristics (see appendix 2), but in general the patient will stay at the ward for the remainder of the day to have further blood tests performed. A cannula will be inserted so that blood can be drawn without the need for repeated venepuncture. A further blood test will be performed the next day, either at the ward or at their home if feasible. The volume of blood from this will not exceed 20 mls.

7.8. Management of patients who become ill

During the study, although this is unlikely, the patient may deteriorate clinically from their COVID-19 or may develop a new intercurrent illness or potentially a side-effect related to the study medication.

All patients who develop difficulty with activities of daily living or complain of shortness of breath will be assessed initially by the study personnel and their oxygen saturation measured and, if necessary, brought to the clinic research physician for further assessment. Based on this assessment, they may be referred to hospital, re-examined the next day, or asked to update the research team by mobile phone frequently on their well-being. The Physician may decide to repeat study oropharyngeal swabs in duplicate if symptoms of COVID-19 recur, for the duration of the illness, to characterize symptomatic viral rebound.

If a patient is referred to hospital, a clinical assessment will be made by the hospital physician and a diagnosis made. The decision to stop study drug will depend on that diagnosis and be made between the hospital team and the study PI.

The hospital physicians will have the responsibility for patient care but the research team will continue to follow the progress of the patients in hospital. Treatment for COVID-19 severe enough to warrant hospitalization will follow national guidelines. For those patients where treatment is changed (i.e. a new treatment is started for clinical reasons) an extra set of swabs in duplicate (and possibly a saliva sample see section 7.1) should be collected prior to the initiation of the new treatment (i.e. 2 sets of swabs will be taken in duplicate). This will only occur if the patient is within the first 7 days of the study.

The D0, D3, D7 and D14 blood samples will be tested for FBC and biochemistry (e.g. U&Es, LFTs) to assess for drug-related adverse effects or abnormalities which require a potential dose adjustment or stopping the therapeutic or clinical intervention. The responsibility for evaluating and acting upon the results of blood tests will belong to the study team at the local site. They will use their clinical judgement in the assessment of the patient, further tests and onwards referral. At any point of the study, extra laboratory tests may be conducted on the patient if felt to be clinically indicated by the research team, based on symptoms or previous laboratory abnormalities.

7.9. Sample Handling and Retention

Samples will be transferred to designated testing facilities, where they will undergo testing in accordance with best practice laboratory measures and safety procedures.

Oropharyngeal swabs (and possibly a saliva sample see section 7.1) will be processed using validated quantitative Real-Time quantitative Polymerase Chain Reaction (qPCR) to detect SARS-CoV-2, according to the study SOP. A urea measurement will be measured on each and compared to the baseline blood concentration. Swabs will be tested in duplicate.

The samples will be retained as per University of Oxford and local site regulations. Consenting patients may rescind their consent up until the completion of the study. Unless requested the samples collected prior to date of withdrawal will be retained for study analysis.

Samples, including those for PK, may be transferred to MORU or other designated testing facilities outside the site country, with appropriate material transfer agreements (MTA) and associated approvals prior to shipment.

8. DISCONTINUATION/WITHDRAWAL OF PATIENTS FROM STUDY

Patients may choose to stop treatment and/or study assessments but may remain on study follow-up. Patients may also withdraw their consent, meaning that they wish to withdraw from the study completely. In the case of withdrawal from both treatment and active follow up the following options for a tiered withdrawal from the study would apply a) Patients withdraw from the study but permit data and samples obtained up until the point of withdrawal to be retained for use in the study analysis. No further data or samples would be collected after withdrawal, b) Patients withdraw from active follow-up and further communication but allow the trial team to continue to access their medical records and any relevant hospital data that is recorded as part of routine standard of care; i.e., CT-Scans, blood results and disease progression data etc.

In addition, the Investigator may discontinue a patient from the trial treatment at any time if the Investigator considers it necessary for any reason including, but not limited to:

- Pregnancy
- Ineligibility (either arising during the study or retrospectively having been overlooked at screening)
- Significant protocol deviation
- Significant non-compliance with treatment regimen or trial requirements
- An adverse event which requires discontinuation of the trial medication or results in inability to continue to comply with trial procedures
- Disease progression which requires discontinuation of the trial medication or results in inability to continue to comply with trial procedures

The reason for discontinuation and/or withdrawal will be recorded in the Case Report Form. qPCR data from patients withdrawn from the study will still be analysed if at least three

distinct timepoints are available for the estimation of a clearance slope. The sample size is adaptive so there is no need to replace withdrawn patients.

Consenting patients may rescind their consent up until the completion of the study. Unless requested, data and samples collected prior to the date of withdrawal will be retained in the study database and analysis.

8.1. Definition of End of Study

The end of study is the date of the 28 day follow up visit of the last enrolled patient.

9. SAFETY REPORTING

9.1. Definitions

Adverse Event (AE)	Any untoward medical occurrence in a patient to whom a medicinal product has been administered, including occurrences which are not necessarily caused by or related to that product.
Adverse Reaction (AR)	An untoward and unintended response in a patient to an investigational medicinal product which is related to any dose administered to that patient.<br>The phrase "response to an investigational medicinal product" means that a causal relationship between a trial medication and an AE is at least a reasonable possibility, i.e. the relationship cannot be ruled out.<br>All cases judged by either the reporting medically qualified professional or the Sponsor as having a reasonable suspected causal relationship to the trial medication qualify as adverse reactions.
Serious Adverse Event (SAE)	A serious adverse event is any untoward medical occurrence that:<br>• results in death<br>• is life-threatening<br>• requires inpatient hospitalisation or prolongation of existing hospitalisation[§]<br>• results in persistent or significant disability/incapacity<br>• consists of a congenital anomaly or birth defect.<br>Other "important medical events" may also be considered serious if they jeopardise the patient or require an intervention to prevent one of the above consequences.<br>NOTE: The term "life-threatening" in the definition of "serious" refers to an event in which the patient was at risk of death at the time of the event; it does not refer to an event which hypothetically might have caused death if it were more severe.

	Hospitalisation is defined as an unplanned, formal inpatient admission, even if the hospitalisation is a precautionary measure for continued observation. The patient must be admitted overnight, a short stay of several hours to receive treatment is not considered hospitalisation. If a patient is admitted overnight or longer for social/economic or isolation reasons and is otherwise medically stable, this does not constitute a SAE. Other examples of visits to a hospital facility that are not considered hospitalisation are: Emergency room visits, outpatient surgery, pre-planned or elective procedures for a pre-existing condition (as long as that condition has not deteriorated while on trial treatment or brought forward because of worsening symptoms) and for the purpose of this study, being hospitalised for COVID-19 isolation.
Serious Adverse Reaction (SAR)	An adverse event that is both serious and, in the opinion of the reporting Investigator, believed with reasonable probability to be due to one of the trial treatments, based on the information provided.

NB: to avoid confusion or misunderstanding of the difference between the terms "serious" and "severe", the following note of clarification is provided: "Severe" is often used to describe intensity of a specific event, which may be of relatively minor medical significance. "Seriousness" is the regulatory definition supplied above.

Any pregnancy occurring during the clinical trial and the outcome of the pregnancy should be recorded and followed up for congenital abnormality or birth defect, at which point it would fall within the definition of "serious".

9.2. Causality

The relationship of each adverse event to the trial medication must be determined by a medically qualified individual according to the following definitions:

Definitely related: There is clear evidence to suggest a causal relationship and other possible contributing factors can be ruled out.

Probably related: There is evidence to suggest a causal relationship and the influence of other factors is unlikely.

Possibly related: There is some evidence to suggest a causal relationship (e.g. because the event occurs within a reasonable time after administration of the trial medication). However, the influence of other factors may have contributed to the event (e.g. the patient's clinical condition, other concomitant treatments).

Unlikely to be related: There is little evidence to suggest there is a causal relationship (e.g. the event did not occur within a reasonable time after administration of the trial medication), or there is another reasonable explanation for the event (e.g. the patient's clinical condition, other concomitant treatment).

Not related: There is no evidence of any causal relationship.

9.3. Procedures for Recording Adverse Events

A symptom questionnaire will be performed daily until D7 with a final follow-up assessment on D28 to aid in the identification of adverse events. The severity of adverse events will be assessed following the Common Terminology Criteria for Adverse Events (CTCAE) v5.0:

1 = mild, 2 = moderate, 3 = severe, 4 = life-threatening, 5 = fatal.

AEs occurring in patients from enrolment and during trial participation that are observed by the Investigator or reported by the patient with severity **grade of 3 (severe) or higher** will be recorded on the CRF, whether or not attributed to trial medication.

When drug combinations are assessed, the team will separately record all clinical AEs (**grade of 1 or higher**) for those given the combination (e.g. molnupiravir **and** nirmatrelvir-ritonavir), as well as those being given the same drugs individually (e.g. molnupiravir **or** nirmatrelvir-ritonavir). This will be important to monitor the safety and tolerability of combinations in direct comparison to the individual drugs.

The following information will be recorded: description, date of onset and end date, severity, assessment of relatedness to trial medication, other suspect drug or device and action taken. Follow-up information should be provided as necessary.

AEs considered related to the trial medication as judged by a medically qualified investigator or the Sponsor will be followed either until resolution, or the event is considered stable.

It will be left to the Investigator's clinical judgment to decide whether or not an AE is of sufficient severity to require the patient's removal from treatment. A patient may also voluntarily withdraw from treatment due to what he or she perceives as an intolerable AE. If either of these occurs, the patient must undergo an end of trial assessment and be given appropriate care under medical supervision until symptoms cease, or the condition becomes stable.

9.4. Grading of laboratory abnormalities

Abnormal laboratory findings detected during the study or are present at baseline and worsen following the taking of study medication will be reported as AEs or SAEs. If found to be abnormal the values will be graded according to the CTCAE v5.0. AEs and SAEs of severity grade 3 or higher will be recorded in the CRF and will be followed up until grade 2 or below, returned to baseline, or deemed to be permanent. For laboratory results that are not available in the CTCAE v5.0, the site investigator should make a determination as to whether or not the laboratory abnormality is clinically significant. If the site investigator believes a laboratory abnormality is clinically significant, it should be reported as an adverse event and a grade should be identified to the best of their ability.

9.5. Reporting Procedures for Serious Adverse Events

All SAEs detected by the site investigator must be reported to the PLATCOV safety team within 24 hours of site awareness. The Serious Adverse Event CRF documenting the SAE should be emailed to PLATCOVsafetyteam@tropmedres.ac. The PLATCOV safety team will inform the DSMB within 10 days of initial notification of the SAE and keep the DSMB updated as needed.

Further reports should be submitted, if required, until the SAE is resolved, is deemed stable/ permanent or results in death. A final status should be determined for any SAEs ongoing at the study end date.

The site PI must also report the SAEs to the local ethics committee and the regulatory authority in accordance with local requirements.

9.5.1 Expectedness

Expectedness will be determined according to the Investigators' Brochure/Summary of Product Characteristics.

9.5.2 SUSAR Reporting

All SUSARs will be reported by the site PI to the relevant Competent Authority and to the local Ethics Committee and other parties as applicable. For fatal and life-threatening SUSARS, this will be done no later than 7 calendar days after MORU is first aware of the reaction. Any additional relevant information will be reported within 8 calendar days of the initial report. All other SUSARs will be reported within 15 calendar days.

Site PIs will be informed of all SUSARs for the relevant IMP that occur at any of the PLATCOV study sites.

9.6. Data Safety Monitoring Board (DSMB)

An independent Data Safety and Monitoring Board (DSMB) will be set up consisting of qualified volunteers

with the necessary knowledge of clinical trials. The DSMB will receive summary reports from MORU as defined per charter or per ad-hoc request, prior to each meeting. All data reviewed by the DSMB will be in the strictest confidence. A DSMB charter will outline its responsibilities and how it will operate.

An interim report will be prepared by the Trial Statistician for the pre-specified interim analysis. In case of safety concerns, additional information or formal interim analyses can be requested by the DSMB.

The DSMB will meet formally at the following timepoints:

- before the study starts
- after the first 50 patients have been accrued into the study (10 per arm)
- At additional time-points as indicated by the DSMB after their review, if deemed necessary

All DSMB recommendations will be communicated to site PIs. The site PI will be responsible for submitting the written DSMB summary reports with recommendations as applicable to local/ national ethics committees and other applicable groups.

10. STATISTICS AND ANALYSIS

10.1. Overview of adaptive study design and overall approach

This is a platform trial that will evaluate multiple antiviral treatments for COVID-19. The sample size is adaptive with multiple planned interim analyses. By specifiying an adaptive sample size, we can more rapidly identify treatments that work and possibly remove treatments that do not work. Clinical trials with adaptive designs are increasingly being used in research, including in such successful and prominent COVID-19 studies as the RECOVERY study. In addition the FDA and EMA supply guidance on studies with these designs[1]. They do not have fixed sample sizes as conventional RCTs. The number of patients recruited depends on the results (i.e. they are adaptive).The benefits of an adaptive sample size are increased efficiency and ethical considerations (i.e. they can stop ineffective arms earlier, identify effective arms earlier, and limit numbers in ineffective arms compared to fixed sample number studies). Because of these advantages this type of study is likely to become more prevalent, but it is important that the study is well-designed in advance, that the adaptive design is appropriate for the overall study design, and has mechanisms in place to control type I error.

The main pharmacodynamic endpoint is the rate of viral clearance, estimated from serial qPCR measurements in each patient (4). There is considerable inter-individual variability in clearance rates which are first order and predominantly monoexponential. The measured end point is the slope of the the initial log linear decline in qPCR estimated viral densities (Figure 5). The clearance rate is correlated with both disease progression and the presence of symptoms. As this is a multi-centre trial, populations may differ between sites with respect to key covariates. Therefore, the main analytic approach will be to fit a hierarchical Bayesian model to the serial qPCR measurements (using default weakly informative Bayesian priors to aid model fitting), whereby the baseline viral loads (intercept) and the first-order clearance rates (slope of initial decline) can vary between sites and between individuals. The viral clearance slope is also dependent on the randomised study arm. Stopping an intervention arm (of type A, B or C) for futility or triggering an intensive PK-PD nested sub-study (type A or C) will be decided using the posterior probabilities that the arm results in an increased slope (increase set as 12.5%, following the results of the first interim analysis). The PK-PD nested sub-study will be also triggered if there is evidence of accelerated viral clearance from other studies (e.g. Molnupiravir, Nirmatrelvir/ritonavir).

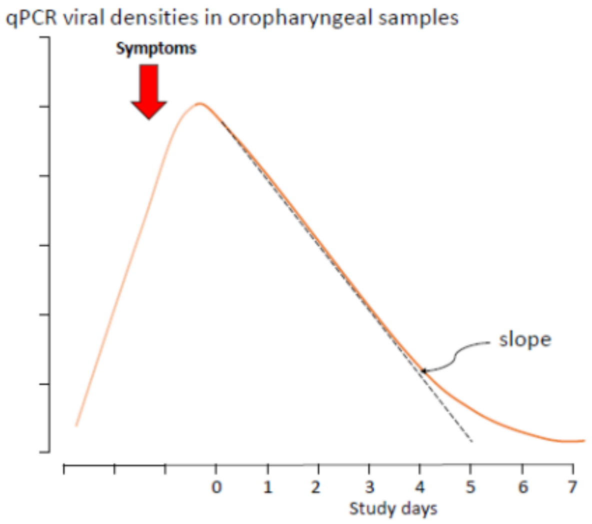

Figure 5 qPCR viral densities in oropharyngeal samples over time, showing time of likely symptom onset relative to viral densities and study days

[1] https://www.fda.gov/media/78495/download
https://www.ema.europa.eu/en/documents/scientific-guideline/reflection-paper-methodological-issues-confirmatory-clinical-trials-planned-adaptive-design_en.pdf

10.2. Results of the first interim analysis

As pre-specified in the SAP, the first interim analysis, triggered once data from the first 50 patients were available, was presented to the Trial Steering Committee and the DSMB. This analysis showed that the positive control arm (REGN-CoV-2) had a substantially larger effect on viral clearance than anticipated, with over 50% increase in viral clearance relative to the negative control arm (Figure 6). In addition, the intra-individual variation in viral load estimation was greater than anticipated, resulting in larger uncertainty around effect sizes. For these reasons, we changed the threshold effect size defining futility and success from 5% increase in viral clearance to 12.5% increase in the viral clearance. Under this threshold, we would need an average of 50 patients randomised to an intervention that has no effect on viral clearance to demonstrate futility.

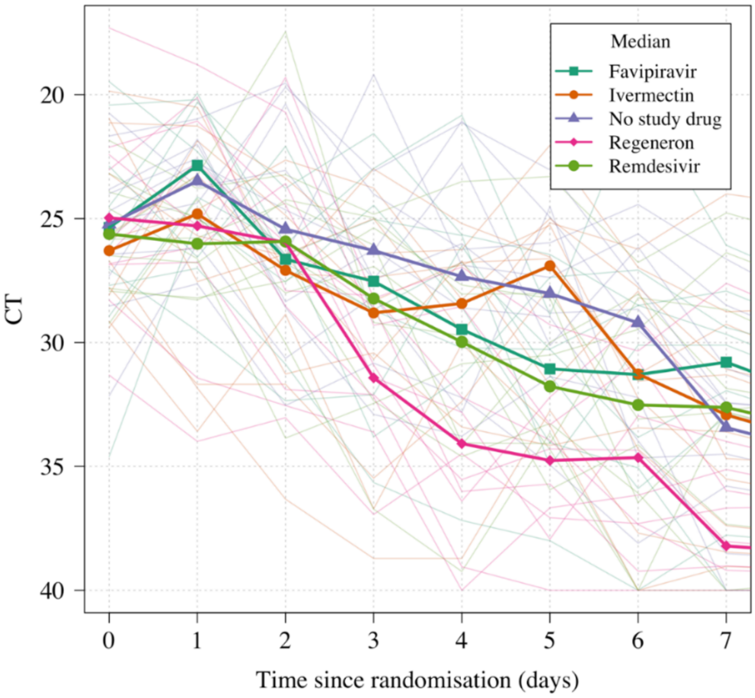

Figure 6 Data from the first interim analysis (first 50 patients recruited into the study).

10.3. Bayesian hierarchical model of viral clearance

At each interim analysis we will fit a series of Bayesian hierarchical (mixed effects) model to the serial viral load measurements (modelled directly on the Ct scale). The main linear model will take the following form:

$$y_{k,i,t} \sim \text{Student}(\alpha_0 + \alpha_k + \alpha_i + (\beta_0 + \beta_i + \beta_k + \beta_{T(i)})t \text{ , sigma}^2\text{, dof})$$

where $y_{k,i,t}$ is the observed $\log_{10}$ viral load for individual i at time t from site k (two values per timepoint), and T(i) is the randomised arm assigned to individual i. Sigma^2 is the measurement error (residual error). The error model is a t-distribution with degrees of freedom inferred from the data (dof). The slope of viral clearance decomposes into 4 terms: the population mean slope β_0; the site random effect $\beta_{k;}$ the individual random effect β_i; and the treatment effect (fixed across sites and individuals) $\beta_{T(i)}$. The intercept term (baseline viral load) decomposes into 3 terms: the population intercept α_0; the site random effect α_k; and the individual random effect α_i. All comparisons are made relative to the no antiviral treatment control arm, so we set $\beta_{control} = 0$. Adjustment will be made for human RNaseP concentration.

The model will treat viral load corresponding to CT values of 40 as left censored. An equivalent non-linear model (allowing for an initial increase in viral load, followed by a linear decrease) will also be fitted to the data as a sensitivity analysis.
The SAP has further details of the analysis.

10.4. Time varying effects

Characterising and identifying time varying effects not specified in the main model is important for two reasons:

- Monoclonal antibodies (type B- see study design section) - where the efficacy can be reduced in infections with spike protein mutant SARS-CoV-2 strains. Therefore, it is possible that a waning effect is observed over time justifying early stopping.
- New interventions (type C- see Study design section) which are added later on during the trial cannot be compared directly to all data from the control arm without accounting for possible differences in population over time (as an epidemic evolves, included populations may differ in a single site over time).

We will use descriptive approaches to detect time-varying effects (residual plots over time, mean slopes in control individuals over time). If substantial time-varying effects are observed, we will add a smooth time varying spline component to the linear model. The base model will not include this for simplicity.

10.5. Sample size estimation, randomisation and statistical considerations for the Bayesian group sequential design

The platform study has two main objectives:

- Identify interventions of type A or B that are unlikely to be of clinical benefit (i.e. demonstrate futility)
- Identify interventions of type A or C that could have clinical benefit and determine optimal dosing (from the nested PK-PD study) for phase 3 evaluations. For type C, these will have stronger *a priori* justification and choosing the best candidate is desired (rather than choosing all candidates with reasonable chance of success)

The required sample size depends on how stringent the thresholds are that define futility or success. Before the trial started we used simulation to determine the probability thresholds that result in control of the type 1 error at 10% and control of the type 2 error at 20% using as a minimum effect size a 5% increase in viral clearance. Following the first interim analysis at 50 patients, the minimum threshold was changed to 12.5%
For the following interim analyses, the decisions made for a treatment arm *T* are as follows:

- Stopping for futility: if Probability($\beta_T > 0.125 * \beta_0$) < 0.1
- Triggering intensive PK-PD nested sub-study: if Probability($\beta_T > 0.125 * \beta_0$) > 0.9

The first interim analysis was carried out after 50 patients were enrolled. We will then carry out interim analyses every 25 patients. The initial thresholds for futility and success were chosen after simulating 6000 trials with all combinations of futility thresholds in the set {0.05, 0.1, 0.2} and success thresholds in the set {0.8, 0.9, 0.95, 0.99} (12 combinations in total, 500 simulations per combination). The maximum sample size was set at 400 patients in total with 6 arms (5 intervention arms and 1 control arm). Out of the 5 interventions, 1 was effective with an effect size of 10% and the other 4 were ineffective (no increase in slope). Type 1 error

for a particular combination of futility and success thresholds was calculated as the average number of times any of the ineffective arms were declared effective divided by the number of decisions (note this the per decision type 1 error not the family wide error rate which is dependent on the number of arms). The type 2 error was calculated as the number of times the effective arm was declared ineffective or no decision was reached by 400 patients. Appendix 3 shows how type 1 and type 2 error varies across choices of thresholds. The thresholds we have chosen allow us to control type 1 errors at 10% and type 2 errors at 20%. For these futility and success thresholds, the simulations demonstrate the number of patients randomised per arm in order to reach a decision is:

- 50 patients (average; median is 30) for each efficacious intervention (effect size of 10% increase in viral clearance) means approximately 60 patients for an effect size of 12.5% increase in viral clearance, as per the current futility/ success threshold 40 patients (average; median is 25) for each inactive intervention (effect size of 0)

Therefore, supposing there were 5 interventions of type A in the platform, and assuming that only 1 of the 5 were in fact effective, we would need on average a total of 50 + 4*40 = 210 patients randomised to interventions of type A (approximately 66% of the total sample size: a minimum of 20% of samples are randomised to the negative control and thus ~13% of patients are randomised to each intervention arm and the positive control arm). This implies a total of ~320 patients for the first set of interventions identified (this does not include any intensive PK sub-studies).

This study is open label and so a centralized server-based randomization system will be used. Only designated study staff will be authorized to access the webapp for patient allocation. The webapp will be password protected. All randomisation activities will be traced within the webapp and attributed to a timestamp along with the anonymised patient study code, age and sex.

10.6. Sample size for the intensive PK-PD sub-study

Any drugs of type A or C that with evidence of accelerated viral clearance from other studies (e.g. Molnupiravir, Nirmatrelvir/ritonavir) or which meet the success endpoint (>90% probability that clearance is accelerated by more than 12.5% relative to control) will be included in an intensive PK-PD study. The sample size calculation for each sub-study will be drug dependent and will use the information regarding the variability in clearance in the intervention arm versus the variability in clearance in the control arm (i.e. it will be determined by the results). Larger observed variability in the intervention arm would be expected if an active intervention had variation in exposures that corresponded to variation around the near maximal effect concentration (eg EC_{95}).

We will use a PK simulation-based approach where we will simulate predicted drug AUC values for patients given the chacteristics of the patients in each site (weight and sex) and use the observed variability in viral clearance rates to estimate the necessary sample size to infer a dose-response relationship whereby a 10% increase in AUC resulted in a 10% increase in the slope of viral clearance with 80% power and 5% type 1 error.

10.7. Final analysis of primary outcome

The final analysis will fit a series Bayesian hierarchical linear and non-linear models to the serial $\log_{10}$ viral load measurements (copies per mL). The primary estimates of interest are the relative changes in the rate of viral clearance for the intervention arms (including the positive control arm) compared to the no study drug arm. These will be reported as their mean posterior estimates along with credible intervals. All analyses will be done using *R* using

the package *rstan* along with bespoke software. All analysis code is openly available via a GitHub repository: https://github.com/jwatowatson/PLATCOV-SAP. Anonymised viral load data and key meta data (e.g. site, strata, randomised allocation) will be made available at publication of results.

11. DATA MANAGEMENT

11.1. Access to Data

Direct access will be granted to authorised representatives from the University of Oxford and any host institution for monitoring and/or audit of the study to ensure compliance with regulations. Outcome data and treatment assignment data will be made available for analysis in real time.

11.2. Data Handling and Record Keeping

Clinical study data will be recorded on CRFs and entered on to a password-protected database by the local study PI, research nurse or designee. The study database will be built in MACRO EDC, a clinical data management system that is compliant with ICH GCP and FDA 21 CFR Part 11 and will be hosted in a secure, access-restricted server in MORU. The study database will include internal quality checks to identify data that appear inconsistent, incomplete, or inaccurate.
Measures will be taken to ensure non-disclosure of information that is potentially harmful to patients. Paper records (for example, patient identifiable information for the purposes of follow-up, the screening logs and signed ICFs) will be kept in locked cabinets; electronic data will only be accessible to staff with user accounts and passwords. The database contains an audit trail that keeps record of changes to data and user activity within the database. All electronic data will be stored on secure servers that are backed up daily, with weekly off-site storage.

Patient records at site will, taking into account the ability of the sites, be stored in binders in the secured access-limited room or scanned and stored electronically. The records will be retained for at least five years following completion of the study, or according to local site regulation. The study database will be retained indefinitely.
With patient's consent, clinical data and results from blood analyses stored in the database may be shared according to the terms defined in the MORU data sharing policy with other researchers to use in the future.
Data generated from this study will adhere to the 2016 "Statement on data sharing in public health emergencies"(https://wellcome.ac.uk/press-release/statement-data-sharing-public-health-emergencies).

12. QUALITY CONTROL AND QUALITY ASSURANCE PROCEDURES

The study will be conducted in accordance with relevant regulations and standard operating procedures.
The study will be conducted in compliance with this protocol, International Conference on Harmonization (ICH) Guidelines for Good Clinical Practice (GCP) and any applicable regulatory requirement(s). Monitoring will be overseen by the MORU Clinical Trials Support Group (CTSG) according to a prespecified risk-based monitoring plan to ensure compliance to the study protocol and applicable guidelines and regulations. Biological specimens will be processed, stored and shipped in accordance with MORU SOPs.

Data validation will be performed to identify errors or discrepancies and thus ensure completeness, validity and accuracy of data.

13. ETHICAL AND REGULATORY CONSIDERATIONS

This study will be conducted in patients who would be unlikely to progress to severe illness. The investigated medications may benefit the patient; i.e. shorten the duration of symptoms or decrease their severity, but are unlikely to have a significant adverse effect on the patient’s illness and subsequent health. No drug intervention has been shown to be unequivocally beneficial at this point in the illness, in this population, and clinical equipoise exists between the interventions and the no antiviral treatment (although local supportive treatment will be fully provided including antipyretics (e.g. paracetamol), anti-tussives, antihistamines, vitamins etc and any treatments clinically indicated by the Physician looking after the patient. Funds will be set aside to cover hospital costs in the unlikely event of a drug adverse reaction. Women who are pregnant, actively trying to become pregnant, or breast feeding will be excluded from this study as it is not known if any of the treatments being tested will have additional benefits that would outweigh any risks associated with pregnancy/breast feeding.

13.1. Declaration of Helsinki

The Investigator will ensure that this study is conducted in accordance with the current revision of the Declaration of Helsinki.

13.2. Guidelines for Good Clinical Practice

The Investigator will ensure that this study is conducted in accordance with relevant regulations and with Good Clinical Practice.

13.3. Approvals

The protocol, informed consent form and participant information sheet will be submitted to OxTREC and local ethics committees for written approval.

The Investigator will submit and, where necessary, obtain approval from the above parties for all amendments to the original approved documents.

13.4. Patient Confidentiality

The study staff will ensure that the patients’ anonymity is maintained. The patients will be identified only by a patient ID number on all study documents and any electronic database, with the exception of the CRF, where patient initials may be added. The name and any other identifying detail will NOT be included in any study data electronic file. All documents will be stored securely and only accessible by study staff and authorised personnel. The study will comply with the Data Protection Act 2018, which requires that personal data must not be kept as identifiable data for longer than necessary for the purposes concerned.

13.5. Expenses

Patients will be financially compensated for their time in the study, in accordance with local EC guidance and approval.

13.6. Risk

All of the treatments being tested *initially (see Appendix 2 for study drugs)* are generally well tolerated with the main adverse effects related to gastrointestinal symptoms (abdominal pain, diarrhoea, nausea and vomiting). In the case of intravenously/subcutaneously

administed treatments there may be discomfort, bleeding or bruising of the skin at the site of needle puncture. Further information regarding specific side effects for each of the drugs used in the study can be found in Appendix 2. These are usually mild and settle without the need for medical intervention.

The risks associated with blood withdrawal during the study include discomfort, occasional bleeding or bruising at the site of needle puncture, and very rarely infection. The risks associated with nose and throat swabbing are limited to some slight discomfort.

13.7. Benefits

It is not yet known if any of the treatments being tested will have additional benefits for the patient in the management of COVID-19. Although an individual patient may not personally benefit, this study should help future COVID-19 patients by discovering treatments that work in early disease and ruling out those do not. Remuneration will be provided to the patients for the period they are enrolled in the study. Patients will be reimbursed for costs associated with traveling to the study site and loss of work time. The amount will be determined per local allowed guidelines and ethics committee policies .

13.8. Reporting

The PI shall submit an Annual Progress Report to OxTREC on the anniversary of the date of approval of the study. In addition, the PI shall submit an End of Study Report to OxTREC within 12 months of completion of the study.

13.9. Finance and insurance

13.9.1 Funding: ACT- Accelerator

This trial is funded by the COVID-19 Therapeutics Accelerator (CTA), managed with MORU through the Wellcome Trust. CTA is a philanthropic collaboration supporting efforts to research, develop and bring effective treatments against COVID-19 to market quickly and accessibly. CTA and Wellcome Trust have had no role in the design of this study and will not have any role during its execution, analyses, interpretation of the data, or decision to submit the results.

13.9.2 Insurance:

The University has a specialist insurance policy in place which would operate in the event of any patient suffering harm as a result of their involvement in the research (Newline Underwriting Management Ltd, at Lloyd's of London).

13.10. Data ownership

The data generated in this study belongs to the study group as a whole. The final database will be shared amongst the site PI and key members of the research team.

The database may be shared with researchers not directly involved in this study but only after the main paper has been published and in accordance with MORU guidelines on data sharing. The database will only be shared if future publications are not compromised. The criteria for authorship will be consistent with the international guidelines (http://www.icmje.org/#author).

13.11. Publication policy

The Investigators will be involved in reviewing drafts of the manuscripts, abstracts, press releases and any other publications arising from the study. Authors will acknowledge that

the study was funded by <*insert name*>. Authorship will be determined in accordance with the International Committee of Medical Journal Editors (ICMJE) guidelines and other contributors will be acknowledged.

The results of the study will be summarised in lay language, in both English and the language(s) commonly spoken at the study sites, and disseminated to key stakeholders, user communities and patients.

14. REFERENCES

1. Organisation WH. COVAX delivers its 1 billionth COVID-19 vaccine dose 2022 [Available from: COVAX delivers its 1 billionth COVID-19 vaccine dose (who.int).
2. He X, Lau EHY, Wu P, Deng X, Wang J, Hao X, et al. Temporal dynamics in viral shedding and transmissibility of COVID-19. Nat Med. 2020;26(5):672-5.
3. Wölfel R, Corman VM, Guggemos W, Seilmaier M, Zange S, Müller MA, Niemeyer D, Jones TC, Vollmar P, Rothe C, Hoelscher M, Bleicker T, Brünink S, Schneider J, Ehmann R, Zwirglmaier K, Drosten C, Wendtner C. Virological assessment of hospitalized patients with COVID-2019. Nature. 2020; 588 (7839):E35. May;581(7809):465-469.
4. Hillen T, Contreras C, Newby JM. Personalized Virus Load Curves of SARS-CoV-2 Infection medRxiv preprint doi: https://doi.org/10.1101/2021.01.21.21250268
5. Watson JA, Kissler SM , Day NPJ, Grad Y, White NJ. Optimal design for phase 2 studies of SARS-CoV-2 antiviral drugs medRxiv preprint doi: https://www.medrxiv.org/content/10.1101/2021.01.06.21249368v1.full.pdf
6. Gastine S, Pang J, Boshier FAT 2, Carter SJ, Lonsdale DO, Cortina-Borja M, Hung IFN, Breuer J, Kloprogge F, Standing JF. Systematic review and patient-level meta-analysis of SARS-CoV-2 viral dynamics to model response to antiviral therapies. medRxiv preprint doi: https://doi.org/10.1101/2020.08.20.20178699
7. Wang D, Hu B, Hu C, Zhu F, Liu X, Zhang J, et al. Clinical Characteristics of 138 Hospitalized Patients With 2019 Novel Coronavirus-Infected Pneumonia in Wuhan, China. JAMA. 2020. 323(11):1061-1069.
8. RECOVERY Collaborative Group, Horby P, Lim WS, Emberson JR, Mafham M, Bell JL, Linsell L, Staplin N, Brightling C, Ustianowski A, Elmahi E, Prudon B, Green C, Felton T, Chadwick D, Rege K, Fegan C, Chappell LC, Faust SN, Jaki T, Jeffery K, Montgomery A, Rowan K, Juszczak E, Baillie JK, Haynes R, Landray MJ. Dexamethasone in Hospitalized Patients with Covid-19 - Preliminary Report. N Engl J Med. 2020 NEJMoa2021436. doi: 10.1056/NEJMoa2021436.
9. Abani O, Abbas A, Abbas F, Abbas M, Abbasi S, Abbass H, et al. Casirivimab and imdevimab in patients admitted to hospital with COVID-19 (RECOVERY): a randomised, controlled, open-label, platform trial. The Lancet. 2022;399(10325):665-76.
10. Weinreich DM, Sivapalasingam S, Norton T, Ali S, Gao H, Bhore R, et al. REGN-COV2, a Neutralizing Antibody Cocktail, in Outpatients with Covid-19. New England Journal of Medicine. 2020;384(3):238-51.
11. Néant N, Lingas G, Le Hingrat Q, Ghosn J, Engelmann I, Lepiller Q, Gaymard A, Ferré V, Hartard C, Plantier JC, Thibault V, Marlet J, Montes B, Bouiller K, Lescure FX, Timsit JF, Faure E, Poissy J, Chidiac C, Raffi F, Kimmoun A, Etienne M, Richard JC, Tattevin P, Garot D, Le Moing V, Bachelet D, Tardivon C, Duval X, Yazdanpanah Y, Mentré F, Laouénan C, Visseaux B, Guedj J; French COVID Cohort Investigators and French Cohort Study groups. Modeling SARS-CoV-2 viral kinetics and association with mortality in hospitalized patients from the French COVID cohort. Proc Natl Acad Sci U S A. 2021 Feb 23;118(8):e2017962118. doi:10.1073/pnas.2017962118. PMID: 33536313.
12. Boschi C, Colson P, Bancod A, Moal V, La Scola B. Omicron Variant Escapes Therapeutic Monoclonal Antibodies (mAbs) Including Recently Released Evusheld®, Contrary to 8 Prior Main Variant of Concern (VOC). Clinical Infectious Diseases. 2022.
13. Zhou H, Tada T, Dcosta BM, Landau NR. Neutralization of SARS-CoV-2 Omicron BA. 2 by therapeutic monoclonal antibodies. Biorxiv. 2022.

14. Feld JJ, Kandel C, Biondi MJ, Kozak RA, Zahoor MA, Lemieux C, Borgia SM, Boggild AK, Powis J, McCready J, Tan DHS, Chan T, Coburn B, Kumar D, Humar A, Chan A, O'Neil B, Noureldin S, Booth J, Hong R, Smookler D, Aleyadeh W, Patel A, Barber B, Casey J, Hiebert R, Mistry H, Choong I, Hislop C, Santer DM, Lorne Tyrrell D, Glenn JS, Gehring AJ, Janssen HLA, Hansen BE. Peginterferon lambda for the treatment of outpatients with COVID-19: a phase 2, placebo-controlled randomised trial. Lancet Respir Med. 2021 Feb 5:S2213-2600(20)30566-X. doi: 10.1016/S2213-2600(20)30566-X. Epub ahead of print. PMID: 33556319.
15. Kissler SM, Fauver JR, Mack C, Tai C, Shiue KY, Kalinich CC, et al. Viral dynamics of SARS-CoV-2 infection and the predictive value of repeat testing. medRxiv. 2020:2020.10.21.20217042.
16. Chaccour C, Casellas A, Blanco-Di Matteo A, Pineda I, Fernandez-Montero A, Ruiz-Castillo P, et al. The effect of early treatment with ivermectin on viral load, symptoms and humoral response in patients with non-severe COVID-19: A pilot, double-blind, placebo-controlled, randomized clinical trial. EClinicalMedicine. 100720. doi: 10.1016/j.eclinm.2020.100720.
17. Pekosz A, Parvu V, Li M, Andrews JC, Manabe YC, Kodsi S, Gary DS, Roger-Dalbert C, Leitch J, Cooper CK. Antigen-Based Testing but Not Real-Time Polymerase Chain Reaction Correlates With Severe Acute Respiratory Syndrome Coronavirus 2 Viral Culture. Clin Infect Dis. 2021 Jan 20: ciaa1706. doi: 10.1093/cid/ciaa1706. Epub ahead of print. PMID: 33479756.
18. Kohmer N, Toptan T, Pallas C, Karaca O, Pfeiffer A, Westhaus S, Widera M, Berger A, Hoehl S, Kammel M, Ciesek S, Rabenau HF. The Comparative Clinical Performance of Four SARS-CoV-2 Rapid Antigen Tests and Their Correlation to Infectivity In Vitro. J Clin Med. 2021;10(2):E328. doi: 10.3390/jcm10020328. PMID: 33477365.
19. Rastawicki W, Gierczyński R, Juszczyk G, Mitura K, Henry BM. Evaluation of PCL rapid point of care antigen test for detection of SARS-CoV-2 in nasopharyngeal swabs. J Med Virol. 2021 Jan 6. doi: 10.1002/jmv.26765.
20. To KK, Tsang OT, Leung WS, Tam AR, Wu TC, Lung DC, et al. Temporal profiles of viral load in posterior oropharyngeal saliva samples and serum antibody responses during infection by SARS-CoV-2: an observational cohort study. Lancet Infect Dis. 2020;20(5):565-74.
21. Sri Santosh T, Parmar R, Anand H, Srikanth K, Saritha M. A Review of Salivary Diagnostics and Its Potential Implication in Detection of Covid-19. Cureus. 2020;12(4):e7708.
22. Williams E, Bond K, Zhang B, Putland M, Williamson DA. Saliva as a Noninvasive Specimen for Detection of SARS-CoV-2. J Clin Microbiol. 2020;58(8).
23. Thompson MG, Ferber JR, Odouli R, David D, Shifflett P, Meece JK, et al. Results of a pilot study using self-collected mid-turbinate nasal swabs for detection of influenza virus infection among pregnant women. Influenza Other Respir Viruses. 2015;9(3):155-60.
24. van Wesenbeeck L, Meeuws H, D'Haese D, Ispas G, Houspie L, Van Ranst M, et al. Sampling variability between two mid-turbinate swabs of the same patient has implications for influenza viral load monitoring. Virol J. 2014;11:233.
25. Corman VM, Landt O, Kaiser M, Molenkamp R, Meijer A, Chu DK, et al. Detection of 2019 novel coronavirus (2019-nCoV) by real-time RT-PCR. Euro Surveill. 2020;25(3).
26. Grein J, Ohmagari N, Shin D, Diaz G, Asperges E, Castagna A, et al. Compassionate Use of Remdesivir for Patients with Severe Covid-19. N Engl J Med. 2020;382(24):2327-36.
27. Piralla A, Daleno C, Pariani E, Conaldi P, Esposito S, Zanetti A, et al. Virtual quantification of influenza A virus load by real-time RT-PCR. J Clin Virol. 2013;56(1):65-8.

28. Kaulbach HC, White MV, Igarashi Y, Hahn BK, Kaliner MA. Estimation of nasal epithelial lining fluid using urea as a marker. J Allergy Clin Immunol. 1993;92(3):457-65.

Appendices

15. Appendix 1: Schedule of activities

Procedures	D0			D1	D2	D3	D4	D5	D6	D7	D10	D14	D28 (-2/ +7)	If new Tx/ symptoms
	Pre-1st dose	H0	H1											
Screening incl. Lateral Flow Ag test and urine pregnancy test	X													
Eligibility assessment	X													
Informed consent	X													
Randomisation	X													
Baseline information captured (inc symptoms, physical exam, vital signs, ECG and POC antibody test)	X													
Symptom checklist			X[1]	X	X	X	X	X	X	X	X	X	X	
Observed dose		X[1]		X[1]	X[1]	X[1]	X[1]	X[1]	X[1]					
Observation post dose			X[1]											
*Temperature**			X	X	X	X	X	X	X	X		X		
Virology														
*Oropharyngeal swabs***	X	X		X	X	X	X	X	X	X	X	X		X
Bloods														
FBC+diff	X[2]					X				X		X		
Biochemistry (including U&Es, LFTs LDH)	X[2]					X				X		X		
Fibrinogen/ FDP	X					X				X		X		
Ferritin	X					X				X		X		

TNF/IL-6/IL-1	X					X				X		X		
Serology and stored sample	X					X				X		X		
Drug level	X					X				X		X		
Host genotyping	X													

* Performed twice a day. For those taking drugs which are more than once per day, the patient will also record the time the dose was taken for any non-observed doses.
** Taken in duplicate. Time swab done recorded. Some patients may be asked to give a saliva sample as well (see section 7.1)

X[1]Depends on dosing schedule of the drug (see appendix 2). The first dose will be given at the designated trial facility. For drugs where further doses are required, at least one dose per day will be observed by the study team. The time the drug is taken will be recorded by the study team.

X[2] Results will be used to detect laboratory abnormalities included in the exclusion criteria (see appendix 4 exclusion criteria)

The final column, If new Tx/symptoms, refers to if the treatment is changed while the patient is within the 1st 7 days of the study, or if new symptoms develop between day 7 and day 28 (see section 7.8 Management of patients who become ill).

Note: PK sampling schedules for determination of antiviral drug level are mentioned in Appendix 2.

16. Appendix 2: Study drugs

The maximum duration of treatment is 7 days so many of the toxicity concerns related to chronic dosing are not relevant to this short course treatment

16.1. Hydroxychloroquine

Rationale

Although hydroxychloroquine and chloroquine have shown anti-SARS-CoV-2 activity *in vitro* (1-4), hydroxychloroquine in hospitalised patients did not result in clinical benefit in two large randomised controlled trials (5,6). Although this is strong evidence that it is ineffective in late stage disease, the evidence earlier in the disease process is equivocal including in early treatment. Meta-analysis of RCTs in pre-exposure prophylaxis is in the direction of benefit. There is therefore substantial uncertainty whether hydroxychloroquine has significant antiviral activity against SARS-CoV-2 in humans.

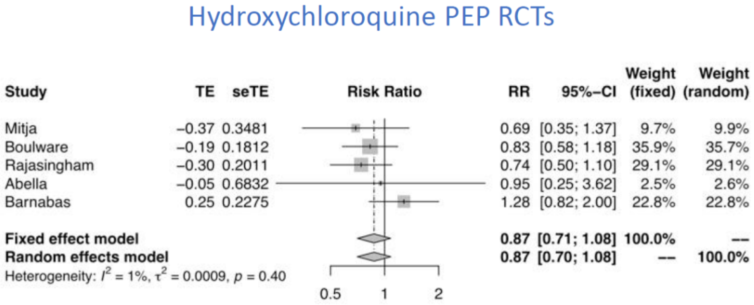

Composition and dose

The reference form of hydroxychloroquine is the Sanofi product, Plaquenil® (https://www.medicines.org.uk/emc/product/1764/smpc).

Hydroxychloroquine will either be obtained by the sponsor or the local teams from a reliable manufacturer. One tablet of hydroxychloroquine sulphate contains 200 mg of hydroxychloroquine sulphate which is equivalent to 155 mg hydroxychloroquine base. Hydroxychloroquine will be given for 7 days.

The dosing schedule of hydroxychloroquine is:

Day 0

- 2 tablets at time 0
- 2 tablets 12 hours later, then

Days 1 – 6

2 tablets per day

That is a total of 800 mg of hydroxychloroquine sulphate (salt) on the first day of treatment and 400 mg of hydroxychloroquine sulphate as a maintenance dose. The doses will be given with food to reduce gastric upset.

Pharmacokinetic characteristics

Hydroxychloroquine is generally well-absorbed, reaches a Cmax at a mean of ~2-3 hours, and following distribution and a multiexponential disposition profile, has a long terminal elimination half-life of ~54 days (5). The disposition of hydroxychloroquine in healthy volunteers is shown in Figure 3 (6).

Pharmacokinetic sampling schedule: (only if there is evidence of accelerated viral clearance)
All patients will need to be admitted for 12 hours for intense venous blood sampling around the **loading dose**.

The timings are:

- Pre-dose D0H0, then
- 1, 2, 3, 4, 6, 9, 12 and 24h post dose

Note that blood will be taken on D3 and D7 for routine haematology and biochemistry. The EDTA sample will be used to measure the drug concentrations.

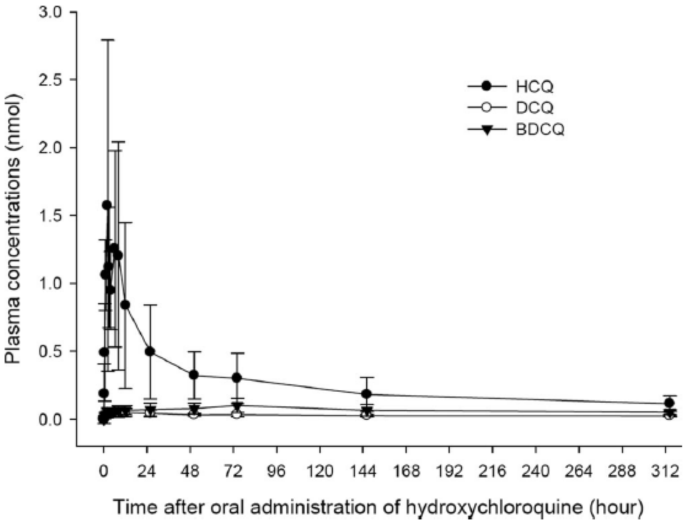

FIG. 3. Plasma concentrations (mean and standard deviation) of HCQ and its metabolites in the six healthy subjects in study Ia before and after the administration of a single oral dose of HCQ sulfate at 400 mg.

Toxicity
Hydroxychloroquine is well-tolerated and has been used for many years to treat patients with rheumatological diseases. The toxicity associated with long term use e.g. retinal disease, myopathy is not seen with short term use.

The most common side effects include dyspepsia, nausea, vomiting, headache and blurred vision; the latter is dose related and usually transient but patients should be warned about driving and operating machinery. Postural hypotension may occur in patients with a fever.

Cardiac toxicity
Hydroxychloroquine (and chloroquine) cause dose related QRS and QT prolongation but these are only clinically important in:

- Overdose (4)
- those with pre-existing QT prolongation
- patients with QT prolonging diseases e.g. myxoedema, ischaemic heart disease, hypokalaemia
- patients on QT prolonging drugs.

The large randomised controlled trials in COVID-19 (RECOVERY and SOLIDARITY) did not find an excess of arrhythmias in patients receiving high dose hydroxychloroquine.

More details regarding concomitant medication can be found at:

- https://www.bnf.org/products/bnf-online/ - available in the UK
- https://www.uptodate.com/drug-interactions/?source=responsive_home#di-druglist – this requires a subscription
- https://compendium.ch/fr/Patient - A Swiss website which deals with drug interactions
- https://crediblemeds.org/

Skin

These include:

exacerbation of psoriasis (case reports)

acute generalised exanthematous pustulosis (AGEP) that may be associated with fever and neutrophilia and resolves with discontinuation of the offending drug

itching / prickly sensation that is more common in dark skinned individuals

Contraindications

In our study, hydroxychloroquine is contraindicated in:

those with known hypersensitivity to hydroxychloroquine or other 4-aminoquinolines (e.g. amodiaquine)

patients with pre-existing retinopathy of the eye

severe liver disease, severe renal disease, epilepsy, porphyria and myasthenia gravis are relative contraindications but such patients would not be enrolled in this trial

Cautions

Long term use of hydroxychloroquine has caused hypoglycaemia in type II diabetes. Diabetic individuals will not be recruited.

Patients with a history of unexplained syncope and/ or family history of sudden unexplained cardiac death, should have an ECG is performed and should be enrolled only if the Bazett QTc <430 ms in males and <450 ms in females.

Relevant drug interactions

Antacids may reduce absorption of hydroxychloroquine so it is advised that a 4-hour interval be observed between hydroxychloroquine sulphate and antacid dosing

References

1. Liu J, Cao R, Xu M, Wang X, Zhang H, Hu H, et al. Hydroxychloroquine, a less toxic derivative of chloroquine, is effective in inhibiting SARS-CoV-2 infection in vitro. Cell Discov. 2020;6:16.
2. Yao X, Ye F, Zhang M, Cui C, Huang B, Niu P, et al. In Vitro Antiviral Activity and Projection of Optimized Dosing Design of Hydroxychloroquine for the Treatment of Severe Acute Respiratory Syndrome Coronavirus 2 (SARS-CoV-2). Clin Infect Dis. 2020.
3. Wang M, Cao R, Zhang L, Yang X, Liu J, Xu M, et al. Remdesivir and chloroquine effectively inhibit the recently emerged novel coronavirus (2019-nCoV) in vitro. Cell Res. 2020;30(3):269-71.
4. White NJ, Watson JA, Hoglund RM, Chan XHS, Cheah PY, Tarning J. COVID-19 prevention and treatment: A critical analysis of chloroquine andhydroxychloroquine clinical pharmacology. PLoS Med 2020; 17: e1003252.
5. RECOVERY group, Horby P, Mafham M, Linsell L, Bell JL, Staplin N, et al. Effect of Hydroxychloroquine in Hospitalized Patients with Covid-19. N Engl J Med. 2020;383(21):2030-40.
6. Consortium WHO Solidarity Trial, Pan H, Peto R, Henao-Restrepo AM, Preziosi MP, Sathiyamoorthy V, et al. Repurposed Antiviral Drugs for Covid-19 - Interim WHO Solidarity Trial Results. N Engl J Med. 2020; 384(6):497-511.

16.2. Remdesivir

Rationale

Remdesivir is a nucleoside analogue prodrug that shows *in vitro* inhibition of the viral RNA polymerase of SARS-CoV (1) and the Middle East Respiratory Syndrome (MERS-CoV) (2) and SARS-CoV-2 (1). In clinical trials, remdesivir has been associated with earlier clinical improvement,hospital discharge and reduced mortality (2-7). A retrospective study showed that administration of remdesivir in hospitalized patients significantly reduced viral load (8).

Composition and dose

Remdesivir will be either be obtained by the sponsor or the local teams. Much of the following is taken from the Gilead summary of product characteristics (SPC) (https://www.medicines.org.uk/emc/product/11597/smpc/print).

Each vial contains remdesivir 100 mg of remdesivir concentrate for solution for infusion. After reconstitution, each vial contains 5 mg/mL of remdesivir solution. The recommended dosage of remdesivir in patients 12 years of age and older and weighing at least 40 kg is:

- Day 1 – single loading dose of remdesivir 200 mg given by intravenous infusion
- Day 2 onwards – 100 mg given once daily by intravenous infusion

The SPC says “The total duration of treatment should be at least 5 days and not more than 10 days.” In our trial we will give remdesivir **for 5 days**.

Remdesivir is given by intravenous infusion in 0.9% saline; there are several options.

Infusion Bag Volume	Infusion Time	Rate of Infusion
250 mL	30 min	8.33 mL/min
	60 min	4.17 mL/min
	120 min	2.08 mL/min
100 mL	30 min	3.33 mL/min
	60 min	1.67 mL/min
	120 min	0.83 mL/min

In our trial, we will give the:

- loading dose in 210 mL of N saline over 60 minutes
- maintenance doses in 230 mL of N saline

Care should be taken to prevent contamination when preparing the remdesivir dose. Use **aseptic technique** and administer IV medicines immediately after preparation.

Recommended dilution instructions – Reconstituted remdesivir powder for concentrate for solution for infusion.

Remdesivir dose	Sodium chloride 9 mg/mL (0.9%) infusion bag volume to be used	Volume to be withdrawn and discarded from sodium chloride 9 mg/mL (0.9%) infusion bag	Required volume of reconstituted remdesivir
200 mg<br>(2 vials)	250 mL	40 mL	2 × 20 mL
100 mg<br>(1 vial)	250 mL	20 mL	20 mL

Pharmacokinetic properties

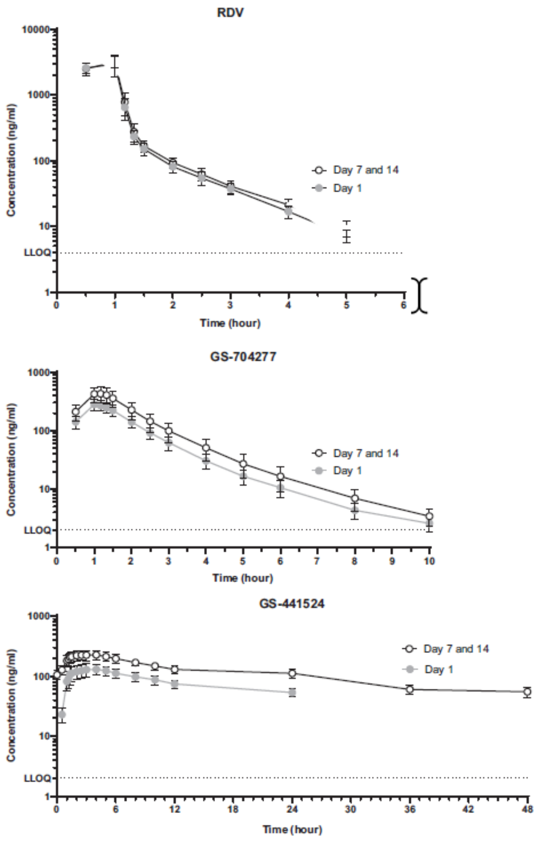

Figure 2 Plasma concentration-vs-time profiles following RDV multiple-dose administration; mean (±SD) values are plotted. LLOQ, lower limit of quantification; RDV, remdesivir.

The pharmacokinetic profile for remdesivir (RDV) and two of its metabolites are shown below (5). Peak plasma concentrations of remdesivir occur at the end of the infusion, declining rapidly thereafter with a half-life of approximately 1 hour. Peak plasma concentrations of the principal biologically active metabolite GS-441524 were observed at 1.5 to 2.0 hours post start of a 30 minute infusion. GS-441524 has a median elimination half-life of 27h.

Remdesivir undergoes extensive intracellular metabolism by esterases to produce several active antiviral metabolites.

Renal clearance is the major elimination pathway for GS-441524 whilst only 10% of RDV is found in urine. With its long half-life, GS-441524 accumulates ~1.9-fold after daily dosing (5).

Pharmacokinetic sampling schedule: (only if there is evidence of accelerated viral clearance)
All patients will need to be admitted for 12 hours for intense venous blood sampling around the **loading dose**.

The timings are:

- Pre-dose D0H0, then

- 1, 2, 3, 4, 6, 9, 12 and 24h post dose

Note that blood will be taken on D3 and D7 for routine haematology and biochemistry. The EDTA sample will be used to measure the drug concentrations.

Toxicity

Remdesivir is generally well-tolerated. The main toxicities are listed below.

Hypersensitivity including infusion-related and anaphylactic reactions

Signs and symptoms may include hypotension, hypertension, tachycardia, bradycardia, hypoxia, fever, dyspnoea, wheezing, angioedema, rash, nausea, vomiting, diaphoresis, and shivering.

Stop the infusion and treat as clinically indicated.

A hypersensitivity reaction contraindicates additional doses.

Transaminase elevations

Transaminase elevations have been observed in healthy volunteers and patients with COVID-19 (9).

The SPC says remdesivir should be discontinued in patients who develop:

- ALT ≥ 5 times the upper limit of normal during treatment with remdesivir
- ALT elevation accompanied by signs or symptoms of liver inflammation or increasing conjugated bilirubin, alkaline phosphatase, or international normalised ratio (INR)

In our study, remdesivir will be stopped if:

- ALT ≥ 5 x ULN
- ALT > 3 x ULN & total bilirubin > 2 x ULN (meeting the Hy's Law criteria) (9)
- Total bilirubin > 2 x ULN (suggesting a cholestatic liver injury)

Common symptoms & signs

These include:

- Headache
- Nausea
- Rashes e.g. maculopapular rash

Contraindications

Known hypersensitivity.

Renal impairment

Patients with eGFR ≥ 30 mL/min/1.73m^2 have received remdesivir for treatment of COVID-19 with no dose adjustment. Remdesivir should not be used in patients with eGFR <30 mL/min/1.73m^2. This is because remdesivir's main metabolite GS-441524 and the excipient, betadex sulfobutyl ether sodium, are renally cleared and accumulate in patients with decreased renal function which may also be related to a reduced volume of distribution (10).

Hepatic impairment

Although the pharmacokinetic characteristics of remdesivir have not been evaluated in liver disease, it should not be used in patients with impaired liver function given its potential liver toxicity.

Relevant drug interactions
No clinical interaction studies have been performed with remdesivir.

In vitro, remdesivir is a substrate for esterases in plasma and tissue, drug metabolising enzymes CYP2C8, CYP2D6, and CYP3A4, and is a substrate for Organic Anion Transporting Polypeptides 1B1 (OATP1B1) and P-glycoprotein (P-gp) transporters but there are no studies of *in vitro* drug interactions.

References

1. Brown AJ, Won JJ, Graham RL, Dinnon KH, 3rd, Sims AC, Feng JY, et al. Broad spectrum antiviral remdesivir inhibits human endemic and zoonotic deltacoronaviruses with a highly divergent RNA dependent RNA polymerase. Antiviral Res. 2019;169:104541.
2. Sheahan TP, Sims AC, Leist SR, Schafer A, Won J, Brown AJ, et al. Comparative therapeutic efficacy of remdesivir and combination lopinavir, ritonavir, and interferon beta against MERS-CoV. Nat Commun. 2020;11(1):222.
3. Grein J, Ohmagari N, Shin D, Diaz G, Asperges E, Castagna A, et al. Compassionate Use of Remdesivir for Patients with Severe Covid-19. N Engl J Med. 2020;382(24):2327-36.
4. Beigel JH, Tomashek KM, Dodd LE, Mehta AK, Zingman BS, Kalil AC, et al. Remdesivir for the Treatment of Covid-19 - Final Report. N Engl J Med. 2020;383(19):1813-26.
5. Humeniuk R, Mathias A, Cao H, Osinusi A, Shen G, Chng E, et al. Safety, Tolerability, and Pharmacokinetics of Remdesivir, An Antiviral for Treatment of COVID-19, in Healthy Subjects. Clin Transl Sci. 2020;13(5):896-906.
6. Diaz G, Christensen A, Pusch T, Goulet D, Chang S, Grunkemeier G et al. Remdesivir and Mortality in Patients With Coronavirus Disease 2019. Clinical Infectious Diseases. 2021;.
7. Gottlieb R, Vaca C, Paredes R, Mera J, Webb B, Perez G et al. Early Remdesivir to Prevent Progression to Severe Covid-19 in Outpatients. New England Journal of Medicine. 2022;386(4):305-315.
8. Biancofiore, A., Mirijello, A., Puteo, M., Di Viesti, M., Labonia, M., Copetti, M., De Cosmo, S. and Lombardi, R., 2022. Remdesivir significantly reduces SARS-CoV-2 viral load on nasopharyngeal swabs in hospitalized patients with COVID-19: A retrospective case–control study. Journal of Medical Virology, pp.1-6.
9. Navarro VJ, Senior JR. Drug-related hepatotoxicity. N Engl J Med. 2006;354(7):731-9.
10. Sorgel F, Malin JJ, Hagmann H, Kinzig M, Bilal M, Eichenauer DA, et al. Pharmacokinetics of remdesivir in a COVID-19 patient with end-stage renal disease on intermittent haemodialysis. J Antimicrob Chemother. 2021; 76(3):825-827.

16.2 Lopinavir/ritonavir

Rationale

Lopinavir is a human immunodeficiency virus 1 (HIV-1) protease inhibitor, which is combined with ritonavir to increase exposure to lopinavir. It is licensed in adults and children. It has been widely used including in pregnant women. Lopinavir has *in vitro* inhibitory activity against SARS coronavirus (SARS-CoV) and MERS-CoV (1). In common marmosets infected with MERS-CoV, animals treated with lopinavir/ritonavir had improved clinical, radiological, and pathological outcomes and reduced viral loads compared with untreated animals. The RECOVERY trial showed no beneficial effect of lopinavir/ritonavir in patients hospitalised with COVID-19, consistent with an earlier study in China, and closed randomisation to that treatment arm (2). However, the main therapeutic window for efficacy of an antiviral drug is earlier in the disease, and there remains uncertainty as to whether an antiviral effect for this drug exists.

Composition & dose

Kaletra® contains 200 mg of liponavir and 50 mg of ritonavir.

Suitable tablets will be obtained by the sponsor or the local teams from a reliable manufacturer.

The dosing schedule is: **2 tablets bd for 7 days**.

Dosing and Pharmacokinetic characteristics

The Pharmacokinetic properties of lopinavir/ritonavir have been evaluated in healthy adult volunteers and in HIV-infected patients; no substantial differences were observed between the two groups. Lopinavir is essentially completely metabolised by CYP3A. Ritonavir inhibits the metabolism of lopinavir, thereby increasing the plasma levels of lopinavir. Administration of lopinavir/ritonavir 400/100 mg twice-daily yields mean steady-state lopinavir plasma concentrations 15- to 20-fold higher than those of ritonavir in HIV-infected patients. The plasma levels of ritonavir are less than 7% of those obtained after the ritonavir dose of 600 mg twice-daily. Figure 1 displays the mean steady-state plasma concentrations of lopinavir and ritonavir after lopinavir/ritonavir 400/100 mg twice-daily with food for 3 weeks from a Pharmacokinetic study in HIV-infected adult subjects (n = 19). This is also the most commonly used and

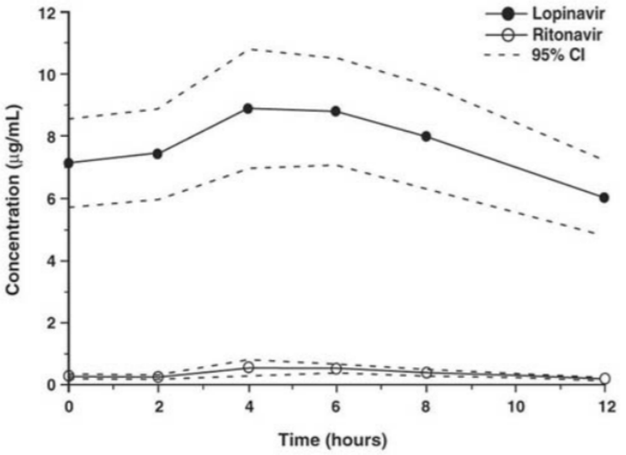

studied lopinavir/ritonavir dosing regimen for COVID-19 treatment (2).

Figure 1. Mean Steady-state Plasma Concentrations with 95% Confidence Intervals (CI) for HIV-Infected Adult Subjects (N = 19)

In a pharmacokinetic study in HIV-positive subjects (n = 19), multiple dosing with 400/100 mg KALETRA twice-daily with food for 3 weeks produced a mean ± SD lopinavir peak plasma concentration (Cmax) of 9.8 ± 3.7 µg/mL, occurring approximately 4 hours after administration. The mean steady-state trough concentration prior to the morning dose was 7.1 ± 2.9 µg/mL and minimum concentration within a dosing interval was 5.5 ± 2.7 µg/mL. Lopinavir AUC over a 12-hour dosing interval averaged 92.6 ± 36.7 µg• h/mL.

Lopinavir is extensively metabolised by the hepatic cytochrome P450 system, almost exclusively by the CYP3A isozyme. Ritonavir is a potent CYP3A inhibitor which inhibits the metabolism of lopinavir, and thereby increases its plasma levels (exposure). Ritonavir has also been shown to induce metabolic enzymes, resulting in the induction of its own metabolism. Pre-dose lopinavir concentrations decline with time during multiple dosing, stabilising after approximately 10 to 16 days.

Pharmacokinetic sampling schedule: (only if there is evidence of accelerated viral clearance)
All patients will need to be admitted for 12 hours for intense sampling.
The timings are:

- Pre-dose D0H0, then
- 1, 2, 3, 4, 6, 9, 12 and 24h post dose.

Note that blood will be taken on D3 and D7 for routine haematology and biochemistry. The EDTA sample will be used to measure the drug concentrations.

Toxicity
Adverse effects of lopinavir/ritonavir include gastrointestinal distress such as nausea and diarrhoea (up to 28%) and hepatotoxicity (2%-10%). In patients with COVID-19, these adverse effects may be exacerbated by viral infection because approximately 20% to 30% of patients have elevated transaminases at presentation with COVID-19.

The following adverse reactions, commonly related to the chronic use of the drug, are discussed in greater detail below:

- Lipid elevations
- Hyperglycaemia
- Pancreatitis
- Hepatotoxicity
- QT Interval Prolongation
- PR Interval Prolongation

Lipid elevations

Treatment with lopinavir/ritonavir has resulted in large increases in the concentration of total cholesterol and triglycerides.

Hyperglycaemia

New onset diabetes mellitus, exacerbation of pre-existing diabetes mellitus, and hyperglycaemia have been reported during post-marketing surveillance in HIV-1 infected patients receiving protease inhibitor therapy. Some patients required either initiation or dose adjustments of insulin or oral hypoglycaemic agents for treatment of these events. In some cases, diabetic ketoacidosis has occurred. In those patients who discontinued protease inhibitor therapy, hyperglycaemia persisted in some cases.

Pancreatitis

Pancreatitis should be considered if clinical symptoms (nausea, vomiting, abdominal pain) or abnormalities in laboratory values (such as increased serum lipase or amylase values) suggestive of pancreatitis occur. Although a causal relationship to lopinavir/ritonavir has not been established, marked triglyceride elevations are a risk factor for development of pancreatitis.

Hepatotoxicity

There have been post marketing reports of hepatic dysfunction, including some fatalities. These have generally occurred in patients with advanced HIV-1 disease taking multiple concomitant medications in the setting of underlying chronic hepatitis or cirrhosis. A causal relationship with lopinavir/ritonavir therapy has not been established.

Elevated transaminases with or without elevated bilirubin levels have been reported in HIV-1 mono-infected and uninfected patients as early as 7 days after the initiation of lopinavir/ritonavir in conjunction with other antiretroviral agents. In some cases, the hepatic dysfunction was serious; however, a definitive causal relationship with lopinavir/ritonavir therapy has not been established. Appropriate laboratory testing should be conducted prior to initiating therapy with lopinavir/ritonavir and patients should be monitored closely during treatment.

QT prolongation

Post marketing cases of QT interval prolongation and Torsade de Pointes have been reported although causality of lopinavir/ritonavir could not be established. Avoid use in patients with congenital long QT syndrome, those with hypokalaemia, and with other drugs that prolong the QT interval.

PR interval prolongation

Lopinavir/ritonavir prolongs the PR interval in some patients. Cases of second- or third-degree atrioventricular block have been reported. Hence, these drugs should be used with caution in patients with underlying structural heart disease, pre-existing conduction system abnormalities, ischemic heart disease or cardiomyopathies, as these patients may be at

increased risk for developing cardiac conduction abnormalities. The impact on the PR interval of co-administration of lopinavir/ritonavir with other drugs that prolong the PR interval (including calcium channel blockers, beta-adrenergic blockers, digoxin and atazanavir) has not been evaluated. As a result, co-administration of lopinavir/ritonavir with these drugs should be undertaken with caution, particularly with those drugs metabolized by CYP3A. Clinical monitoring is recommended.

Contraindications

In our study, lopinavir/ritonavir is contraindicated in:

hypersensitivity to lopinavir/ritonavir or any of its ingredients, including ritonavir.

co-administration with drugs highly dependent on CYP4503A.

co-administration with potent CYP450 3A inducers

More details regarding concomitant medication can be found at:

https://www.covid19-druginteractions.org/checker

https://crediblemeds.org/

https://compendium.ch/fr/Patient - this is a Swiss website dealing with drug interactions

https://www.bnf.org/products/bnf-online/ - available only in the UK

References

1. Chu CM, Cheng VC, Hung IF, Wong MM, Chan KH, Chan KS, et al. Role of lopinavir/ritonavir in the treatment of SARS: initial virological and clinical findings. Thorax. 2004;59(3):252-6.
2. Cao B, Wang Y, Wen D, Liu W, Wang J, Fan G, et al. A Trial of Lopinavir-Ritonavir in Adults Hospitalized with Severe Covid-19. N Engl J Med. 2020;382(19):1787-99.

16.3. Miglustat

Rationale

Miglustat is a drug that is in current clinical use for lysosomal storage disorders and has shown to be active against a variety of viral infections. A key mechanism by which iminosugars act as antivirals is their ability to disrupt glycoprotein folding, arising from the inhibition of ER α-glu I and II (1). Miglustat has been shown to inhibit SARS-CoV-2 in different cell types at concentrations compatible with those obtained for the treatment of Gaucher's disease and Niemann-Pick type C patients (2), although more recent evidence in the calu-3 cell line suggests that higher doses (those used in the treatment of HIV may be required to reach *in vivo* antiviral drug concentrations) (unpublished communication). Time of addition studies indicate that the inhibitory activity is at the post-entry level and affects release of infectious virus. The proper folding and release of the Spike protein, and progeny virus appear mostly affected.

Composition & dose

Zavesca® capsules contain 100 mg of miglustat.

Suitable tablets will be obtained by the sponsor or the local teams from the manufacturer.

The approved doses are:

- 100 mg TDS and 200 mg TDS are approved doses (3) but doses of up to 3000mg/ day have been used in HIV patients for up to 6 months.

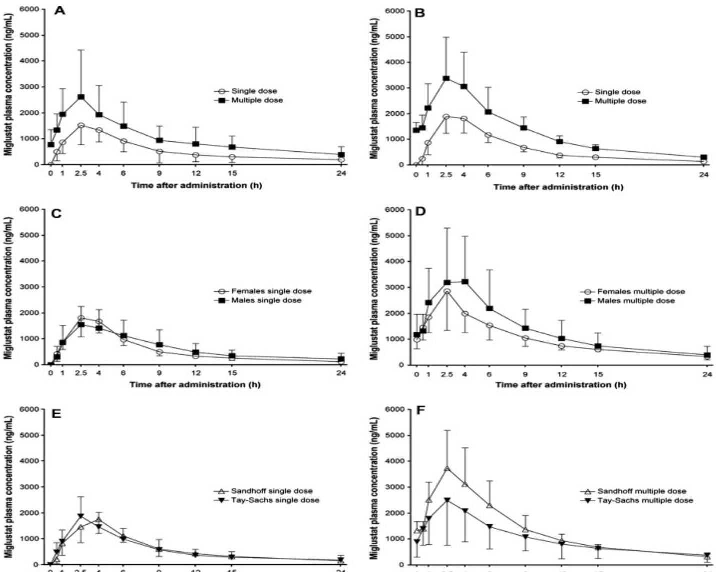

In our study the dose will be using the highest dose of **1g TDS PO for 7 days**

Pharmacokinetic characteristics

In a study of 28 adult patients with Gaucher's disease receiving 100mg three times daily the time of maximum plasma concentration and half-life were 2.5 and 6.3 h, respectively, with a maximum concentration of 0.86 µg/mL. Steady-state plasma concentrations were achieved after 4–6 weeks of treatment (3).

Miglustat plasma exposure reaches steady state at about 72 h and the ratio trough/peak is overall between 0.6 and 0.7.

Pharmacokinetic sampling schedule: (only if there is evidence of accelerated viral clearance)

All patients will need to be admitted for 12 hours for intense sampling.

The timings are:

- Pre-dose D0H0, then
- 1, 2, 3, 4, 6, 9, 12 and 24h

Note that blood will be taken on D3 and D7 for routine haematology and biochemistry. The EDTA sample will be used to measure the drug concentrations.

Toxicity

This drug is commonly used in chronic conditions, and the toxicities described reflect that situation. It was also evaluated at doses of up to 3000 mg/day (the dose being evaluated in this study for 7 days) for up to six months in Human Immunodeficiency Virus (HIV)-positive patients. In these studies, the adverse events included granulocytopenia, dizziness, and paraesthesia. Leukopenia and neutropenia have also been observed in a similar group of patients receiving 800 mg/day or above.

- Gastrointestinal disturbances

Gastrointestinal events, mainly osmotic diarrhoea, have been observed in more than 80% of patients, either at the outset of treatment or intermittently during it. The mechanism is most likely inhibition of intestinal disaccharidases in the gastrointestinal tract. In clinical practice, miglustat-induced gastrointestinal events have been observed to respond to individualised diet modification, to taking it between meals, and/or to use anti-diarrhoeal medicinal products such as loperamide. In some patients, temporary dose reduction may be necessary (4).

- Fertility

After a single study in rodents suggested that miglustat could interfere with spermatogenesis there were concerns over reproductive toxicity. However these concerns have been allayed by studies in humans which have not shown effects on spermatogenesis (5). Contraceptive measures should be used by women of childbearing potential as miglustat crosses the placenta and should not be used during pregnancy. Studies in animals have shown reproductive toxicity, including dystocia. The potential risk for humans is unknown. It should also not be taken during breastfeeding.

- Renal impairment

Due to limited experience, miglustat should be used with caution in patients with renal or hepatic impairment. This drug is not recommended in patients with severe renal impairment (creatinine clearance < 30 ml/min/1.73m^2).

Contraindications

Known hypersensitivity.

Cautions

Female patients should use contraception while receiving treatment and continue for one week after finishing. There are no relevant drug interactions

References

1. Alonzi DS, Scott KA, Dwek RA, Zitzmann N. Iminosugar antivirals: the therapeutic sweet spot. Biochem Soc Trans. 2017;45(2):571-82.
2. Williams SJ, Goddard-Borger ED. Alpha-glucosidase inhibitors as host-directed antiviral agents with potential for the treatment of COVID-19. Biochem Soc Trans. 2020;48(3):1287-95.
3. Maegawa GH, van Giersbergen PL, Yang S, Banwell B, Morgan CP, Dingemanse J, et al. Pharmacokinetics, safety and tolerability of miglustat in the treatment of pediatric patients with GM2 gangliosidosis. Mol Genet Metab. 2009;97(4):284-91.

4. Kuter DJ, Mehta A, Hollak CE, Giraldo P, Hughes D, Belmatoug N, et al. Miglustat therapy in type 1 Gaucher disease: clinical and safety outcomes in a multicenter retrospective cohort study. Blood Cells Mol Dis. 2013;51(2):116-24.
5. Amory JK, Muller CH, Page ST, Leifke E, Pagel ER, Bhandari A, Subramanyam B, Bone W, Radlmaier A, Bremner WJ. Miglustat has no apparent effect on spermatogenesis in normal men. Hum Reprod. 2007 Mar;22(3):702-7. doi: 10.1093/humrep/del414. Epub 2006 Oct 25. PMID: 17067996.

16.4. Ivermectin

Rationale

Ivermectin is a macrocyclic lactone and broad-spectrum antiparasitic, antibacterial, and antiviral activity. It has an excellent safety profile and with 3.5 billion treatments distributed by mass drug administration for onchocerciasis and lymphatic filariasis elimination in Africa and Latin America since 1987 (Mectizan Donation Program). The side effects of ivermectin largely relate to the body's reaction to the death of parasites (Mazzotti-type reaction) but is otherwise generally well-tolerated (1,2). Ivermectin has demonstrated broad-spectrum *in vitro* antiviral activity and is being used on a number of continents in the management of COVID-19. There are also reports from trials suggesting benefit and given the low cost, safety, tolerability and availability of this drug, it would be an attractive candidate in the prevention and treatment of COVID-19, if shown to have benefit in this illness.

Ivermectin has *in vitro* activity against the novel coronavirus SARS-CoV-2, which causes coronavirus disease (COVID-19). Ivermectin also inhibits influenza, HIV, Dengue, West Nile Virus, Yellow Fever virus, Japanese encephalitis, tick-borne encephalitis virus and Venezuelan equine encephalitis virus *in vitro* (2,3). The EC_{50} (the concentration to decrease the cytopathic effect of the virus by 50%) for SARS-CoV-2 on modified African green monkey kidney (Vero/hSLAM) cells was reportedly ~2µM and a 99.8% reduction in viral RNA at 48hrs in samples treated with 5µM concentrations (2), although the relevance of this *in vitro* cell line to human disease, like with other *in vitro* models, remains uncertain.

During the COVID-19 pandemic, due to early pre-print publications of the benefit of Ivermectin, this drug has been used widely at different stages of the disease, at different doses, for different durations and outside of clinical trials. RCTs of questionable quality seem to suggest improvements in virologic and clinical parameters, some demonstrating very large benefits.

Composition & dose

One tablet of ivermectin as STROMECTOL® contains 3 mg of ivermectin (https://mri.cts-mrp.eu/Human/Downloads/NL_H_3952_001_FinalSPC.pdf) where 3mg tablets are not available a corresponding dosing table will be created based on the dosing of 600mcg/kg bodyweight.

Ivermectin will either be obtained by the sponsor or the local teams from a reliable manufacturer.

The dose in this study is **600 micrograms/kg body weight given once daily for 7 days** (taken with food).

**Weight in kg**	**Number of 6 mg tablets**	**Dose in mg**	**mg/kg**
40 - <50	4	24	0.49 - 0.6
50 - <60	5	30	0.51 - 0.6

60 - <70	6	36	0.52 - 0.6
70 - <80	7	42	0.53 - 0.6
80 - <90	8	48	0.54 - 0.6
90 - <100	9	54	0.55 - 0.6
≥ 100	10	60	≤ 0.6

Pharmacokinetic characteristics

Ivermectin is incompletely absorbed (~50% bioavailable relative to an oral hydroalcoholic solution) following oral doses of ivermectin tablets, with a Tmax of ~4 hours.

Ivermectin has a molar mass of 875.1g/mmol, and as such 2µM (concentration at which anti-SARS-CoV-2 activity has been demonstrated) corresponds to a concentration of 1750 µg/L. Following oral administration of ivermectin with a standard oral dose of 12 mg (mean <200 µg/kg) of oral Ivermectin producing a C_{max} of 23.5-50µg/L (4,5). At this dose the C_{max} in plasma is some 35 times lower than the EC_{50} of the virus and an appreciable *in vivo* effect would not be expected to occur. However, in other experiments, *in vitro* effects of ivermectin do not adequately account for the *in vivo* effects observed. Baby hamster kidney (BHK-21) cells infected with pseudorabies virus exhibited an ivermectin EC_{50} of ~2µM at 72hours, which is similar to that of SARS-CoV-2, and would lead one to expect that a therapeutic response would not be possible. However, published in the same paper, single dose ivermectin (200 µg/kg) administered concomitantly or 12 hours after pseudorabies virus inoculation was able to suppress viral DNA synthesis and save 60% of infected mice. A 200 µg/kg ivermectin dose in mice has a human equivalent dosing (HED) of 16.3 µg/kg. The reason behind this *in vitro* to *in vivo* disparity is unclear but may relate to a more active ivermectin metabolite as is seen with this drug in other indications, as was suggested by a paper showing a 20- to 35-fold increase in mortality of mosquitoes which fed on blood from single dose ivermectin (400 µg/kg) treated volunteers compared to *in vitro* ivermectin spiked blood (6).

In humans, a phase III randomised, placebo-controlled, double blind trial showed a statistically significant difference in Dengue NS1 clearance times between ivermectin (400 or 600 µg/kg for three days) and placebo, although this did not translate into clinical efficacy (7). Again, this antiviral effect was seen despite *in vitro* EC_{50}s against dengue which would suggest adequate *in vivo* levels would not be achieved (an order of magnitude lower).

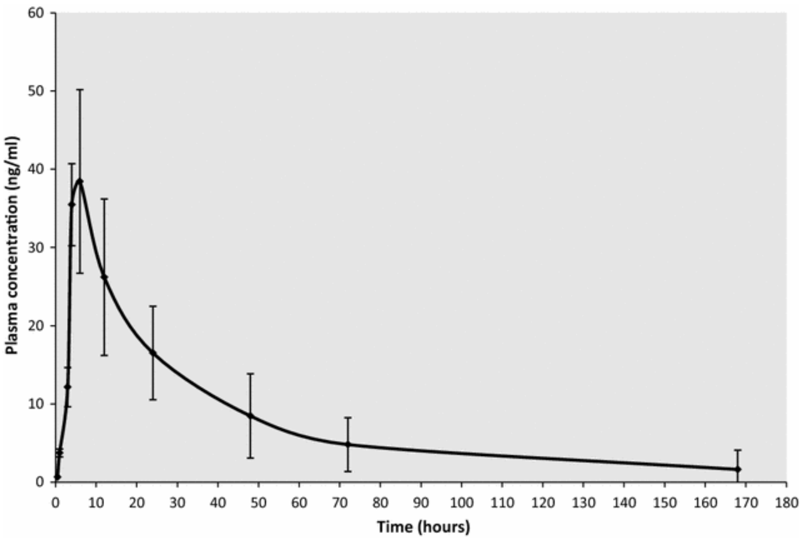

Administration of 30 mg (333 to 600 µg/kg) ivermectin following a high-fat meal resulted in an approximate 2.5-fold increase in bioavailability compared to fasted healthy volunteers.

Ivermectin is metabolised in humans, and ivermectin and/or its metabolites are excreted almost exclusively in the faeces over an estimated 12 days with less than 1% of the administered dose being excreted in the urine. The plasma half-life of ivermectin in man is about 12 hours (9.8-14.3 h) and that of the metabolites is about 3 days.

Pharmacokinetic sampling schedule: (only if there is evidence of accelerated viral clearance)
All patients will need to be admitted to hospital for 12 hours for intense sampling. The timings are:

- Pre-dose D0H0, then
- 1, 2, 3, 4, 6, 9, 12 and 24h

Note that blood will be taken on D3 and D7 for routine haematology and biochemistry. The EDTA sample will be used to measure the drug concentrations.

Toxicity
Risks related to ivermectin are very low. The side effects of ivermectin largely relate to the body’s reaction to the death of parasites (Mazzotti-type reaction) with Onchocerciasis but is otherwise generally well-tolerated, and would not be an issue if studied in a non-endemic region.

Contraindications
Hypersensitivity reaction to ivermectin

Cautions
Ivermectin has not been studied in patients with impaired hepatic function or impaired renal function.

Relevant Drug interactions

None

References

1. Zoure HG, Wanji S, Noma M, Amazigo UV, Diggle PJ, Tekle AH, et al. The geographic distribution of Loa loa in Africa: results of large-scale implementation of the Rapid Assessment Procedure for Loiasis (RAPLOA). PLoS Negl Trop Dis. 2011;5(6):e1210.
2. Crump A, Omura S. Ivermectin, 'wonder drug' from Japan: the human use perspective. Proc Jpn Acad Ser B Phys Biol Sci. 2011;87(2):13-28.
3. Mastrangelo E, Pezzullo M, De Burghgraeve T, Kaptein S, Pastorino B, Dallmeier K, et al. Ivermectin is a potent inhibitor of flavivirus replication specifically targeting NS3 helicase activity: new prospects for an old drug. J Antimicrob Chemother. 2012;67(8):1884-94.
4. Caly L, Druce JD, Catton MG, Jans DA, Wagstaff KM. The FDA-approved Drug Ivermectin inhibits the replication of SARS-CoV-2 in vitro. Antiviral Res. 2020:104787.
5. Gonzalez Canga A, Sahagun Prieto AM, Diez Liebana MJ, Fernandez Martinez N, Sierra Vega M, Garcia Vieitez JJ. The pharmacokinetics and interactions of ivermectin in humans--a mini-review. AAPS J. 2008;10(1):42-6.
6. Kobylinski KC, Jittamala P, Hanboonkunupakarn B, Pukrittayakamee S, Pantuwatana K, Phasomkusolsil S, et al. Safety, Pharmacokinetics, and Mosquito-Lethal Effects of Ivermectin in Combination With Dihydroartemisinin-Piperaquine and Primaquine in Healthy Adult Thai Subjects. Clin Pharmacol Ther. 2019; 107(5):1221-1230.
7. Yamasmith, E., et al., Efficacy and Safety of Ivermectin against Dengue Infection: A Phase III, Randomized, Double-blind, Placebo-controlled Trial, in the 34th Annual Meeting The Royal College of Physicians of Thailand- ‘Internal Medicine and One Health'. 2018: Chonburi, Thailand.

16.5. Nitazoxanide

Rationale

Nitazoxanide is a broad-spectrum antiprotozoal and antiviral drug first discovered in the 1980s. It has an excellent safety profile, the only contraindication to nitazoxanide being a hypersensitivity reaction to nitazoxanide. Nitazoxanide has demonstrated broad-spectrum *in vitro* antiviral activity including against SARS-CoV-2. There are also reports from trials suggesting benefit and given the low cost, safety, tolerability and availability of this drug, it would be an attractive candidate in the prevention and treatment of COVID-19, if shown to have benefit in this illness. Nitazoxanide has *in vitro* activity against the novel coronavirus SARS-CoV-2, which causes coronavirus disease (COVID-19). The EC50 on Vero E6 cells (the concentration to decrease the cytopathic effect of the virus by 50%) was reportedly 2.12µM (1). Nitazoxanide has a molar mass of 307.283g/mmol, and as such 2.12µM corresponds to a concentration of 0.65mg/L.

Although the above study on SARS-CoV-2 used nitazoxanide, the active metabolite tizoxanide inhibits influenza, respiratory syncytial virus, parainfluenza, coronavirus, rotavirus, norovirus, hepatitis B, hepatitis C, dengue, yellow fever, Japanese encephalitis virus and human immunodeficiency virus *in vitro* (2). Nitazoxanide was able to protect 90% of mice from death in a lethal challenge model of Japanese Encephalitis (3). The exact mechanism by which nitazoxanide exhibits its antiviral effects are uncertain, but augmented host responses as opposed to direct antiviral effects are postulated. Nitazoxanide is able to increase interferon responses in HCV/HIV coinfected individuals (4).

A double-blind randomised placebo-controlled trial of nitazoxanide in acute uncomplicated influenza found a statistically decreased duration of symptoms in the 600mg BD nitazoxanide group, but not the 300mg BD one (5). Additionally, viral titres were statistically decreased in the 600mg BD nitazoxanide group. Another study using the same doses in cases of severe respiratory infection caused by all respiratory viruses, did not show a statistical effect (6). Nitazoxanide is being assessed in clinical trials in COVID-19, and published results suggest an increased time to viral clearance, although this result may be confounded by differences in initial viral loads (7).

Composition & dose

One tablet of nitazoxanide as ALINIA® tablets contain 500 mg of nitazoxanide (https://www.accessdata.fda.gov/drugsatfda_docs/label/2016/021497s001,021498s004lbl.pdf).

Nitazoxanide will either be obtained by the sponsor or the local teams from a reliable manufacturer.

High dose nitazoxanide is given as 1.5 g bd.

The dose in this trial is: **1.5g PO twice daily for 7 days** (taken with food).

Pharmacokinetic characteristics

Following oral administration of nitazoxanide, the drug is rapidly hydrolysed to its active constituent, tizoxanide, and then conjugated to produce active tizoxanide glucuronide; nitazoxanide is not detected in plasma and > 99% of tizoxanide is bound to proteins. The bioavailabilities of tizoxanide and tizoxanide glucuronide are increased 2-fold with food. The mean Tmax of tizoxanide, is 2.5h with a half-life of 1 to 2h (8). The mean Tmax of the tizoxanide glucuronide is 4 to 6h. A dose of 1g PO BD of nitazoxanide produces a Cmax of 24mg/ml of tizoxanide and 26.4mg/ml tizoxanide glucuronide and the half-lives increase to 6.4 and 3.5 hours, respectively. Tizoxanide is excreted in the urine, bile and faeces, and tizoxanide glucuronide is excreted in urine and bile (its concentration is x10 that of plasma).

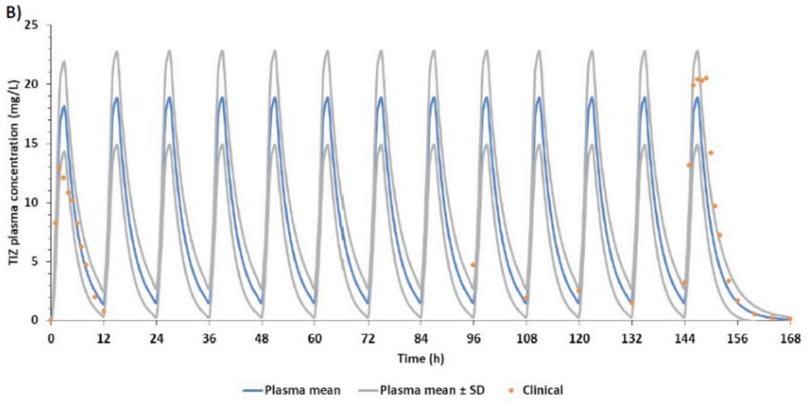

Pharmacokinetic sampling schedule: (only if there is evidence of accelerated viral clearance)

All patients will need to be admitted for 12 hours for intense sampling. The timings are:

- Pre-dose D0H0, then
- 1, 2, 3, 4, 6, 9, 12 and 24h

Note that blood will be taken on D3 and D7 for routine haematology and biochemistry. The EDTA sample will be used to measure the drug concentrations.

Toxicity

Nitazoxanide is a safe and well-tolerated drug. Reported toxicities include:

Gastrointestinal disorders: diarrhoea, gastroesophageal reflux disease

Nervous System disorders: dizziness

Respiratory, thoracic and mediastinal disorders: dyspnoea

Skin and subcutaneous tissue disorders: rash, urticaria

Risks related to nitazoxanide are very low. This is a very safe and generally well tolerated medication but adverse reactions relating to the central nervous system (headache and rare reports of dizziness), the gastrointestinal system (abdominal pain and nausea and rare reports of diarrhoea and gastro-oesophageal reflux), and genitourinary system (urine discolouration) have been described as well as post-marketing cases of dyspnoea, skin rash and urticaria. In one study, side effects of nitazoxanide did not differ from those of the placebo when used for the treatment of giardiasis, and does not cause side effects when used in healthy adults (9). This lack of significant side-effects is seen in doses up to 4g in humans. The LD50 of various animals is 10g/Kg. A study of 365 patients with AIDS receiving nitazoxanide 500-1500mg twice daily for a median duration of 62 days (1-1528 days) concluded that "No safety issues were identified at doses up to 3000 mg/day or for long durations of treatment" (10). These risks will be mitigated by excluding participation if people have had a previous serious adverse reaction to nitazoxanide.

Nitazoxanide may be used after the first trimester of pregnancy with severe symptoms of cryptosporidiosis. Adverse events have not been observed in animal reproduction studies although human data are not available. It is not known whether nitazoxanide enters into breast milk, and the manufacturer suggests balancing risk of exposure to the infant and benefit to the mother. We will exclude pregnant women and women breastfeeding from this study and ask female patients of child-bearing age to undergo a pregnancy test and take precautions not to become pregnant (contraception). Those who do become pregnant will be followed up. However, given the lack of evidence of harmful effects and its use later in pregnancy, the risk to a foetus seems low. The mitigating factors of a pregnancy test and advising contraception will make the likelihood of pregnancy within the trial and potential risk, even lower.

Contraindications and cautions

Known hypersensitivity. The Pharmacokinetics of nitazoxanide in patients with compromised renal or hepatic function have not been studied.

Relevant Drug interactions

None

<u>References</u>

1. Rossignol JF. Nitazoxanide: a first-in-class broad-spectrum antiviral agent. Antiviral Res. 2014;110:94-103.
2. Shi Z, Wei J, Deng X, Li S, Qiu Y, Shao D, et al. Nitazoxanide inhibits the replication of Japanese encephalitis virus in cultured cells and in a mouse model. Virol J. 2014;11:10.
3. Petersen T, Lee YJ, Osinusi A, Amorosa VK, Wang C, Kang M, et al. Interferon Stimulated Gene Expression in HIV/HCV Coinfected Patients Treated with Nitazoxanide/Peginterferon-Alfa-2a and Ribavirin. AIDS Res Hum Retroviruses. 2016;32(7):660-7.
4. El-Kowrany SI, El Ghaffar AEA, Shoheib ZS, Mady RF, Gamea GAM. Evaluation of nitazoxanide as a novel drug for the treatment of acute and chronic toxoplasmosis. Acta tropica. 2019;195:145-54.
5. Haffizulla J, Hartman A, Hoppers M, Resnick H, Samudrala S, Ginocchio C, et al. Effect of nitazoxanide in adults and adolescents with acute uncomplicated influenza: a double-blind, randomised, placebo-controlled, phase 2b/3 trial. Lancet Infect Dis. 2014;14(7):609-18.
6. Gamino-Arroyo AE, Guerrero ML, McCarthy S, Ramirez-Venegas A, Llamosas-Gallardo B, Galindo-Fraga A, et al. Efficacy and Safety of Nitazoxanide in Addition to Standard of Care for the Treatment of Severe Acute Respiratory Illness. Clin Infect Dis. 2019;69(11):1903-11.

7. Rocco PRM, Silva PL, Cruz FF, Junior M, Tierno P, Moura MA, et al. Early use of nitazoxanide in mild Covid-19 disease: randomised, placebo-controlled trial. Eur Respir J. 2021. Jan 14:2003725. doi: 10.1183/13993003.03725-2020.
8. Stockis A, Deroubaix X, Lins R, Jeanbaptiste B, Calderon P, Rossignol JF. Pharmacokinetics of nitazoxanide after single oral dose administration in 6 healthy volunteers. Int J Clin Pharmacol Ther. 1996;34(8):349-51.
9. Stockis A, Allemon AM, De Bruyn S, Gengler C. Nitazoxanide pharmacokinetics and tolerability in man using single ascending oral doses. Int J Clin Pharmacol Ther. 2002;40(5):213-20.
10. Rossignol JF. Nitazoxanide in the treatment of acquired immune deficiency syndrome-related cryptosporidiosis: results of the United States compassionate use program in 365 patients. Aliment Pharmacol Ther. 2006;24(5):887-94.

16.6. REGN-COV2

Rationale

REGN-COV2 is an antibody cocktail of two anti-SARS-CoV-2 neutralising antibodies Casirivimab and Imdevimab, which has been shown to reduce viral load in patients with COVID-19 (1). On the 21st of November 2020 the US Food and Drug Administration (FDA) issued an Emergency Use Authorization (EUA) in people who are older than 12 years, weigh more than 40Kg and were at high risk of progression to severe disease. The drug is currently being evaluated in the RECOVERY trial in late-stage hospitalised patients, but as with all antiviral drugs, the likely window of benefit is earlier in disease, when viral burdens are higher and before people are hospitalised, when inflammation predominates. Although REGN-COV-2 is currently expensive and requires IV administration- thereby limiting its use globally, this antibody cocktail currently has the best evidence for an antiviral effect, and will form the positive control for other interventions (i.e. interventions can be assessed in relation to the likely antiviral effect observed in this positive control arm). The hope would be that the cost, availability and feasibility of administration will improve and that this may become a more widely available intervention for COVID-19, which can also be used in LMICs. Unfortunately, the potential benefit of antibody therapy, including REGN-COV-2 is threatened by the appearance of mutant viruses, and there is now evidence of decreased binding affinity for the receptor binding domain (RBD) of Casirivimab, of the South Africa variant with the K417N and/or E484K mutations. As such the study will additionally allow monitoring of the effect of this antibody cocktail on viral clearance over time and with the appearance of different viral mutants, informing its on-going utility.

Composition & dose

The dosage in adults and in pediatric patients (12 years of age and older weighing at least 40 kg) is 600 mg of casirivimab and 600 mg of imdevimab administered together as a single intravenous infusion over at least 60 minutes. Casirivimab and imdevimab solutions must be diluted prior to administration. Casirivimab and imdevimab should be given together as soon as possible after positive results of direct SARS-CoV-2 viral testing and within 10 days of symptom onset.

The dose in this trial is: **600mg casirivimab/ 600mg imdevimab given once on D0**

REGN-COV2 will either be obtained by the sponsor or the local teams from the manufacturer.

For preparation and administration instructions please see:
https://www.fda.gov/media/143892/download

Pharmacokinetic characteristics

Pharmacokinetic profiles of casirivimab and imdevimab are expected to be consistent with the profile of other IgG1 mAbs.

Specific Populations- the effect of different covariates (e.g., age, sex, race, body weight, disease severity, hepatic impairment) on the pharmacokinetics of casirivimab and imdevimab is unknown. Renal impairment is not expected to impact the pharmacokinetics of casirivimab

and imdevimab, since mAbs with molecular weight >69 kDa are known not to undergo renal elimination.

Drug-Drug Interactions- casirivimab and imdevimab are mAbs which are not renally excreted or metabolized by cytochrome P450 enzymes; therefore, interactions with concomitant medications that are renally excreted or that are substrates, inducers, or inhibitors of cytochrome P450 enzymes are unlikely

Pharmacokinetic schedule: only if there is evidence of accelerated viral clearance
As a type B intervention (positive control), intensive pharmacokinetic sampling will not be conducted on those receiving REGN-COV2.
Toxicity
There is a potential for serious hypersensitivity reaction, including anaphylaxis, with administration of casirivimab and imdevimab. If signs or symptoms of a clinically significant hypersensitivity reaction or anaphylaxis occur, immediately discontinue administration and initiate appropriate medications and/or supportive therapy. Infusion-related reactions have been observed with administration of casirivimab and imdevimab. Signs and symptoms of infusion-related reactions may include: fever, chills, nausea, headache, bronchospasm, hypotension, angioedema, throat irritation, rash including urticaria, pruritus, myalgia, dizziness. If an infusion-related reaction occurs, consider slowing or stopping the infusion and administer appropriate medications and/or supportive care.

Contraindications and Cautions
There are no contraindications.

<u>Reference</u>

1. Weinreich DM, Sivapalasingam S, Norton T, Ali S, Gao H, Bhore R, et al. REGN-COV2, a Neutralizing Antibody Cocktail, in Outpatients with Covid-19. New England Journal of Medicine. 2020;384(3):238-51.

16.8 Nebulised Unfractionated Heparin (UFH)

Rationale

Nebulised UFH has demonstrated antiviral, anti-inflammatory, anticoagulant and mucolytic effects and is being trialled in various phases of COVID-19. In this trial, we aim to primarily establish the antiviral effect of this drug *in vivo*, although other effects of this drug may also be clinically relevant in COVID-19. In laboratory experiments, UFH blocked the binding and infectivity of SARS-CoV-2 to human bronchial epithelial cells and in a Vero E6 cell line, inhibition of SARS-CoV-2 infection occurred at therapeutically relevant concentrations. Use of UFH via the nebulisation allows for higher concentrations to be delivered to infected tissues, without concerns re: systemic effects of anticoagulation. UFH is a cheap and widely available drug which can be given nebulised in the community. If shown to be effective, this treatment may be studied in phase 3 studies to determine the effect on clinical endpoints, and affordable and widely available nebulising systems can be developed (1).

Composition & dose

25000 IU UFH every 6 hours using a vibrating mesh nebuliser for 7 days.

Pharmacokinetic characteristics

UFH is rapidly taken up by respiratory endothelium and clinically significant systemic effects are not expected.

Pharmacokinetic schedule: only if there is evidence of accelerated viral clearance

As we are using the nebulised route of administration, high levels of drug will be expected in the oropharynx and respiratory tract, but negligible levels in the systemic circulation. An intensive pharmacokinetic schedule will not be conducted.

Toxicity

Toxicity relating to use of UFH normally relates to systemic effects of the drug on coagulation (through prolongation of the aPTT and increased likelihood of bleeding) and reactions to the drug. At the proposed dose used in this study, nebulised UFH only modestly increases peak aPTT in patients who concomitantly receive IV or SC UFH and has no effect on the peak APTT in patients who receive IV or SC LMWH.

Contraindications (1)

- Heparin allergy or Heparin-induced thrombocytopaenia
- Known prolonged APTT > 120ms or bleeding diathesis
- Pulmonary bleeding or uncontrolled bleeding
- Platelet count < 20 x 10^9 /L
- Pregnant

Cautions

In addition to those with the above contraindications, caution should be taken by those using the nebulised system, as this may cause aerosolisation of virus and potentially increase the likelihood of transmission. As such, nebulisation if it occurs at the ward should occur in a negative pressure room with appropriate infection control procedures. If nebulisation occurs

at home, this should be done in a room with adequate ventilation to the outside, where the patient can guarantee that the room will not be entered by any other persons for at least 2 hours after nebulisation occurs. Ideally this will be conducted outside, at least 50 metres from the nearest person.

Drug interactions

The listed drug interactions for UFH relate to systemic use and are not relevant to its use as nebulised therapy. Those taking drugs with an increased risk of bleeding should be assessed and potentially excluded.

Reference: https://www.fda.gov/media/143892/download

Reference

1. van Haren FMP, Richardson A, Yoon HJ, Artigas A, Laffey JG, Dixon B, Smith R, Vilaseca AB, Barbera RA, Ismail TI et al: INHALEd nebulised unfractionated HEParin for the treatment of hospitalised patients with COVID-19 (INHALE-HEP): Protocol and statistical analysis plan for an investigator-initiated international metatrial of randomised studies. Br J Clin Pharmacol 2020.

16.9 Favipiravir

Rationale

Favipiravir is a small molecule antiviral which was developed in 2002 as an inhibitor of viral replication of influenza (1). Favipiravir is a pyrazinecarboxamide derivative, thought to act as a selective viral RNA-dependent RNA polymerase inhibitor. Favipiravir is a prodrug which is metabolized to its active form, favipiravir-ribofuranosyl-5'-triphosphate. Favipiravir has demonstrated *in vitro* activity against SARS-CoV-2 (2). An early open labelled control study compared Favipiravir and Lopinavir/ritonavir. It found that the favipiravir arm had a shorted viral clearance median time and significant improvement in chest CT compared to the control arm (improvement rate 91.43% compared to 62.22% - p=0.004) (3). A systematic review and meta-analysis were performed by Hassanipour et.al. (4). It showed a significant clinical improvement in favipiravir compared to control during the 7 days post hospitalization (RR = 1.24, 95% CI: 1.09–1.41; P = 0.001). Viral clearance was higher with favipiravir but it was not significant. Transfer to ICU and adverse events were not found to be significant. In those with mild to moderate COVID-19 there was no beneficial effect to mortality.

Composition & dose

In this study, patients will be given 1800mg of favipiravir (nine 200mg tablets) at H0 and 1800mg of favipiravir (9 200mg tablet) at H12. Thereafter the patient will take 800mg BD (four 200mg tablets) for a further 6 days.

Favipiravir will either be obtained by the sponsor or the local teams from a reliable manufacturer.

Pharmacokinetic characteristics

Oral favipiravir reaches maximal concentrations at 2 hours post oral administration and has a half-life of 2-5.5 hours. Protein binding occurs at 54%. The active parent drug is metabolised to the inactive metabolite T-705M1 mainly by aldehyde oxidase and partially by xanthine oxidase. PK analysis of continuous doses of favipiravir in nonhuman primate models exhibit nonlinear pharmacokinetics over time, with decreasing trough levels at D4, relative to D2.

Pharmacokinetic schedule: only if there is evidence of accelerated viral clearance

All patients will need to be admitted for 12 hours for intense sampling. The timings are:

- Pre-dose D0H0, then
- 1, 2, 3, 4, 6, 9, 12 and 24h

Note that blood will be taken on D3 and D7 for routine haematology and biochemistry. The EDTA sample will be used to measure the drug concentrations.

Toxicity

A review of the safety of favipiravir from 29 studies demonstrates a favourable safety profile. Side-effects which tend to be mild, include diarrhoea, as well as blood abnormalities including raised uric acid levels, low neutrophil count and raised liver function tests. Early concerns regarding QTc prolongation, were not born out in a study of healthy Japanese individuals using moxifloxacin as a positive control, where favipiravir showed no effect on QT intervals.

Animal models have demonstrated teratogenicity, and no human studies on pregnant or lactating women have been conducted.

Contraindications & cautions

Known hypersensitivity. See later section on drug-specific contraindications.

Patients should avoid excessive exposure to sunlight or artificial UV light

Drug interactions

Favipiravir inhibits the formation of acetaminophen sulphate *in vitro* and *in vivo*, resulting in a small magnitude increase in acetaminophen levels. As a result daily acetaminophen (paracetamol) should not exceed 3g/ day (5).

References

1. Furuta, Y., Takahashi, K., Fukuda, Y., Kuno, M., Kamiyama, T., Kozaki, K., Nomura, N., Egawa, H., Minami, S., Watanabe, Y., Narita, H. and Shiraki, K., 2002. In Vitro and In Vivo Activities of Anti-Influenza Virus Compound T-705. Antimicrobial Agents and Chemotherapy, 46(4), pp.977-981.
2. Wang M, Cao R, Zhang L, Yang X, Liu J, Xu M et al. Remdesivir and chloroquine effectively inhibit the recently emerged novel coronavirus (2019-nCoV) in vitro. Cell Research. 2020;30(3):269-271.
3. Cai, Q., Yang, M., Liu, D., Chen, J., Shu, D., Xia, J., Liao, X., Gu, Y., Cai, Q., Yang, Y., Shen, C., Li, X., Peng, L., Huang, D., Zhang, J., Zhang, S., Wang, F., Liu, J., Chen, L., Chen, S., Wang, Z., Zhang, Z., Cao, R., Zhong, W., Liu, Y. and Liu, L., 2020. Experimental Treatment with Favipiravir for COVID-19: An Open-Label Control Study. Engineering, 6(10), pp.1192-1198.
4. Hassanipour, S., Arab-Zozani, M., Amani, B., Heidarzad, F., Fathalipour, M. and Martinez-de-Hoyo, R., 2021. The efficacy and safety of Favipiravir in treatment of COVID-19: a systematic review and meta-analysis of clinical trials. Scientific Reports, 11(1).
5. Zhao Y, Harmatz JS, Epstein CR, Nakagawa Y, Kurosaki C, Nakamura T, Kadota T, Giesing D, Court MH, Greenblatt DJ. Favipiravir inhibits acetaminophen sulfate formation but minimally affects systemic pharmacokinetics of acetaminophen. Br J Clin Pharmacol. 2015 Nov;80(5):1076-85. doi: 10.1111/bcp.12644. Epub 2015 Jun 8. PMID: 25808818; PMCID: PMC4631180.

16.10 Molnupiravir

Rationale

Molnupiravir is a small molecule ribonucleoside prodrug of N-Hydroxycytidine (NHC). It is phosphorylated intracellularly into NHC triphosphate (NHC-TP). NHC-TP incorporates into viral RNA by viral RNA polymerase causing an accumulation of deleterious errors into the viral genome. This causes "error catastrophe" by increasing the rate of mutation in the viral genome to a level lethal to the virus and causing extinction [13].

Studies of mice infected with SARS-CoV-1 found that EDII-2801 (an orally bioavailable NHC-prodrug) prophylactically and therapeutically improved pulmonary function, and reduced virus titre [14]. An *in vivo* study on human lung-only mice found that molnupiravir dramatically inhibited the replication of SARS-CoV-2 [15]. A Phase 1 safety trial of doses up to 1600MG per day found molnupiravir was well tolerated with the incidence of adverse effects was highest in the placebo arm [16]. Another Phase 1 trial concluded molnupiravir was safe and well tolerated and advised 800MG BD for 5 days [17].

A Phase 2 trial compared viral clearance to a range of doses and found that by day 5, virus was not isolated for those receiving 400 or 800MG BD of molnupiravir (0/42 and 0/53 respectively), compared to 11.1% (6/54) in the placebo group (p = 0.03).

The drug manufacturer, Merck, Sharpe & Dohme (MSD), performed a double blind randomised, placebo-controlled trial, which gave molnupiravir within 5 days of symptoms to non-hospitalised, unvaccinated adults with mild/moderate COVID-19 and at least 1 risk factor for severe COVID-19. Dose was 800MG BD for 5 days. Primary efficacy end point was hospitalisation due to any cause or death at day 29. Interim results showed that 28 out of 285 (7.3%) patients given molnupiravir had been hospitalised or died, compared to 53 out of 377 (14.1%) in the placebo group (-6.8% difference, −11.3 to −2.4; P=0.001). In addition the all-randomised analysis also showed that molnupiravir treatment was associated with greater reductions from baseline in mean viral load than placebo at days 3, 5 and 10 [18].

However, when the data was presented to the FDA for approval, further analysis showed lower effectiveness. A press release showed data from all enrolled patients and found that the risk of hospitalisation in the molnupiravir group was 9.7% (68/699) compared to 6.8% (48/709) in the control group. The absolute risk reduction was 3.0% (0.1 to 5.9; p-value =0.0218). A 50% decrease from the interim results [19].

Composition & dose

Each Lagevrio® capsule contains 200 mg of molnupiravir, recommended dose is 800mg taken orally every 12 hours for 5 days. Molnupiravir is being made by MSD, as well as under license by a number of generics manufacturers.

Molnupiravir will either be obtained by the sponsor or the local teams from a reliable manufacturer (including high-quality generics manufacturers).

Should be given within 5 days of onset of symptoms.

Pharmacokinetic characteristics

Molnupiravir is a prodrug metabolised to the ribonucleoside analogue N-Hydroxycytidine (NHC) and phosphorylated to the active ribonucleoside triphosphate (NHC-TP). Following twice daily oral administration of 800 mg molnupiravir, median time to peak plasma NHC concentrations is 1.5 hours. The effective half-life of NHC is approximately 3.3 hours. It can be taken with or without food.

Pharmacokinetic sampling schedule: (only if there is evidence of accelerated viral clearance)
The timings are:

- Pre-dose D0H0, then
- 1, 2, 3, 4, 6, 9, 12 and 24h post dose

Note that blood will be taken on D3 and D7 for routine haematology and biochemistry. The EDTA sample will be used to measure the drug concentrations.

Toxicity
Highest dose tested in safety trials was 800MG BD, found to have a 0.9% probability of having 30% excess toxicity compared to control therefore considered safe [17].

Contraindications
Hypersensitivity

Cautions
No dose adjustment needed for renal impairment however the pharmacokinetics have not been evaluated with eGFR <30 mL/min, also not evaluated with hepatic impairment.
As per USA FDA advice, those on molnupiravir need to be aware of avoiding pregnancy. Females of child bearing potential need to use a reliable method of contraception for the duration of the treatment, and also for 4 days after the final dose of molnupiravir. Males of reproductive potential, if they are sexually active with females of child bearing potential, should use a reliable form of contraception during the treatment and at least 3 months after the final dose.

Therefore, in addition to the baseline pregnancy test, a repeat test on women of childbearing potential who are allocated to Molnupiravir will be done on D14.

Drug Interactions

None identified. There are no theoretical drug interactions in combination nirmatrelvir-ritonavir. Molnupiravir and its metabolites are not substrates of the CYP enzymes, and are broken down into uridine and cytidine through the normal intracellular pyrimidine catabolism pathways.

References

1. Singh AK, Singh A, Singh R, Misra A. Molnupiravir in COVID-19: A systematic review of literature. Diabetes & Metabolic Syndrome: Clinical Research & Reviews. 2021;15(6):102329.
2. Sheahan TP, Sims AC, Zhou S, Graham RL, Hill CS, Leist SR, et al. An orally bioavailable broad-spectrum antiviral inhibits SARS-CoV-2 and multiple endemic, epidemic and bat coronavirus. Biorxiv. 2020.
3. Wahl A, Gralinski LE, Johnson CE, Yao W, Kovarova M, Dinnon KH, et al. SARS-CoV-2 infection is effectively treated and prevented by EIDD-2801. Nature. 2021;591(7850):451-7.
4. Painter WP, Holman W, Bush JA, Almazedi F, Malik H, Eraut NC, et al. Human safety, tolerability, and pharmacokinetics of molnupiravir, a novel broad-spectrum oral antiviral agent with activity against SARS-CoV-2. Antimicrobial agents and chemotherapy. 2021;65(5):e02428-20.
5. Khoo SH, Fitzgerald R, Fletcher T, Ewings S, Jaki T, Lyon R, et al. Optimal dose and safety of molnupiravir in patients with early SARS-CoV-2: a Phase I, open-label, dose-escalating, randomized controlled study. Journal of Antimicrobial Chemotherapy. 2021;76(12):3286-95.
6. Jayk Bernal A, Gomes da Silva MM, Musungaie DB, Kovalchuk E, Gonzalez A, Delos Reyes V, et al. Molnupiravir for oral treatment of Covid-19 in nonhospitalized patients. New England Journal of Medicine. 2021.
7. Merck and Ridgeback Biotherapeutics Provide Update on Results from MOVe-OUT Study of Molnupiravir, an Investigational Oral Antiviral Medicine, in At Risk Adults With Mild-to-Moderate COVID-19 [press release]. 26/November/2021 2021.

16.11 Nirmatrelvir/ritonavir (e.g. PAXLOVID™)

Rationale

PAXLOVID™ is a therapeutic combination of 2 compounds, Nirmatrelvir and ritonavir, taken at the same time as separate pills. Nirmatrelvir (PF-07321332) is an oral covalent 3CL protease inhibitor of SARS-CoV-2 and ritonavir is an inhibitor of HIV-1 and HIV-2 protease. Ritonavir inhibits cytochrome P450A and CYP2D6 which inhibits the metabolism of nirmatrelvir.

Coronoviruses contain two proteases, main protease – Mpro (also known as 3CL protease – 3CLpro) and papain-like protease. Pfizer developed a SARS-CoV Mpro inhibitor in 2002 (PF-00835231) which demonstrated in vitro and in vivo activity against SARS-CoV-2 [20]. Other studies also found successful in vitro inhibition of SARS-CoV-2 replication in derived alveolar basal epithelial cells expressing ACE2 [21] and human bronchial epithelial cells [22]. This compound had insufficient bioavailability so the oral version PF-07321332 was developed [23].

A Phase 2/3 EPIC-HR randomised double blind control trial from Pfizer looked at non-hospitalised patients at risk of progressing to severe illness. Intervention was ritonavir and nirmatrelvir started within three or five days of symptom onset. Primary endpoint was COVID-19-related hospitalisation or all cause death up to day 28. For those receiving PAXLOVID™ within 3 days, 3/389 (0.7%) were hospitalised with zero deaths., compared to the placebo 27/385 hospitalised with 7 deaths, $p<0.0001$. For treatment within 5 days, 6/607 (6.7%) were hospitalised with no deaths, compared to 41/612 (with 10 deaths) in the placebo group ($p<0.0001$) [24].

Composition & dose

Administered as 300MG of nirmatrelvir (two 150MG tablets) with one 100 MG tablet of ritonavir given twice daily for 5 days.

Nirmatrelvir/ritonavir will either be obtained by the sponsor or the local teams from a reliable manufacturer (including high-quality generics manufacturers).

Pharmacokinetic characteristics

Ritonavir is administered with nirmatrelvir as a pharmacokinetic enhancer resulting in higher systemic concentrations and longer half-life of nirmatrelvir. This supports a twice daily administration. Nirmatrelvir (when given with ritonavir) is eliminated renally, with a half-life of 6.05 hours, peak concentration is at 3 hours. Ritonavir is metabolised in the liver, with a half-life of 6.15 hours, peak concentration is at 3.98 hours.

Pharmacokinetic sampling schedule: (only if there is evidence of accelerated viral clearance)

The timings are:

- Pre-dose D0H0, then
- 1, 2, 3, 4, 6, 9, 12 and 24h post dose

Note that blood will be taken on D3 and D7 for routine haematology and biochemistry. The EDTA sample will be used to measure the drug concentrations.

Toxicity

Repeat-dose toxicity studies up to 1 month duration of nirmatrelvir in rats and monkeys resulted in
no adverse findings. Ritonavir can cause retinal toxicity with long term use [25], although this is not relevant for the short-term use in the study. Ritonavir alone does not cause any issues related to HIV drug-resistance.

Contraindications

In our study, nirmatrelvir/ritonavir is contraindicated in:

hypersensitivity to nirmatrelvir/ritonavir or any of its ingredients, including ritonavir.

co-administration with drugs highly dependent on CYP4503 A.

co-administration with potent CYP450 3A inducers

More details regarding concomitant medication can be found at:

https://www.covid19-druginteractions.org/checker

https://crediblemeds.org/

https://compendium.ch/fr/Patient - this is a Swiss website dealing with drug interactions

https://www.bnf.org/products/bnf-online/ - available only in the UK

Cautions

Caution should be given in patients with pre-existing liver disease/hepatitis or live enzyme abnormalities. Dose reduction for renal impairment (eGFR ≥ 30 to <60 mL/min) is 150MG nirmatrelvir and 100 MG ritonavir.

Drug Interactions

Contraindicated with drugs that are highly dependent on CYP3A for clearance or are potent CYP3A inducers (see above). There are no theoretical drug interactions in combination molnupiravir.

References

1. Boras B, Jones RM, Anson BJ, Arenson D, Aschenbrenner L, Bakowski MA, et al. Discovery of a novel inhibitor of coronavirus 3CL protease as a clinical candidate for the potential treatment of COVID-19. BioRxiv. 2021:2020.09. 12.293498.
2. de Vries M, Mohamed AS, Prescott RA, Valero-Jimenez AM, Desvignes L, O'Connor R, et al. A comparative analysis of SARS-CoV-2 antivirals characterizes 3CLpro inhibitor PF-00835231 as a potential new treatment for COVID-19. Journal of Virology. 2021;95(10):e01819-20.
3. Barilli A, Visigalli R, Ferrari F, Di Lascia M, Riccardi B, Puccini P, et al. Organic Cation Transporters (OCTs) in EpiAirway™, a Cellular Model of Normal Human Bronchial Epithelium. Biomedicines. 2020;8(5):127.
4. Owen DR, Allerton CM, Anderson AS, Aschenbrenner L, Avery M, Berritt S, et al. An oral SARS-CoV-2 Mpro inhibitor clinical candidate for the treatment of COVID-19. Science. 2021:eabl4784.
5. Mahase E. Covid-19: Pfizer's paxlovid is 89% effective in patients at risk of serious illness, company reports. British Medical Journal Publishing Group; 2021.
6. Tu Y, Poblete RJ, Freilich BD, Zarbin MA, Bhagat N. Retinal toxicity with ritonavir. International journal of ophthalmology. 2016;9(4):640.

16.12 Sotrovimab

Rationale

Sotrovimab (VIR-7831) is an Fc-engineered IgG1 human monoclonal antibody developed from a parental antibody isolated from a survivor of the SARS outbreak in 2003 [26]. Sotrovimab targets a highly conserved epitope in the SARS-CoV-2 spike protein at a region that does not compete with angiotensin-converting enzyme 2 binding.

In-vitro testing has shown that sotrovimab is able to retain activity against alpha, beta, gamma, delta and kappa variants, and even omicron pseudotyped virus encoding the then most prevalent haplotype of the Omicron spike. The same study showed that Syrian Golden hamsters infected with wildtype SARS-CoV-2 had significantly decreased total viral load and infectious virus levels with sotrovimab [27].

A multicentre randomized, double-blind, multicentre, placebo-controlled, phase 3 study tested sotrovimab on non-hospitalised patients with symptomatic, mild to moderate COVID-19 and at least 1 risk factor for disease progression. Symptom onset was within 5 days, median age was 53. Primary efficacy outcome was all-cause hospitalization longer than 24 hours for acute illness management or death through day 29. Primary outcome was significantly reduced with sotrovimab (6/528 – 1%) compared to placebo (30/529 – 6%) by 79% (95% CI, 50% – 91%; P<.001) [28].

N.B. – REGN-COV2 (the initial positive control monoclonal antibody) was shown in vitro studies to fail to neutralise omicron [29], although the in-vivo significance of this is yet to be determined.

Composition & dose

Recommended dose is 500MG sotrovimab administered as a single intravenous infusion over 60 minutes. Patients should be monitored for at least 1 hour after administration.

0.9% Sodium Chloride of 5% dextrose for injection, one vial of sotrovimab is 500MG/8ML.

Pharmacokinetic characteristics

The mean systemic clearance is 125 mL/day, and the median terminal half-life is approximately 49 days. The geometric mean maximum plasma concentration (Cmax) after a 1-hour sotrovimab intravenous infusion to be 117.6 mcg/mL, and the geometric mean Day 29 concentration to be 24.5 mcg/mL.

Toxicity

Serious hypersensitivity reactions, including anaphylaxis, have been reported with administration of

sotrovimab. If signs or symptoms of a clinically significant hypersensitivity reaction or anaphylaxis occur during infusion, immediately discontinue administration and initiate appropriate medications and/or supportive care.

Contraindications

Hypersensitivity

Cautions

Renal impairment is not expected to impact the pharmacokinetics of sotrovimab since monoclonal antibodies with molecular weight >69 kDa do not undergo renal elimination. The effect of hepatic insufficiency is unknown. The effect of other covariates (e.g., sex, race, body weight, disease of severity on the pharmacokinetics is also unknown.

Pharmacokinetic schedule: only if there is evidence of accelerated viral clearance

As a type B intervention (positive control), intensive pharmacokinetic sampling will not be conducted on those receiving sotrovimab.

Drug Interactions

No formal drug interaction studies have been performed with sotrovimab. Sotrovimab is not renally
excreted or metabolized by cytochrome P450 (CYP) enzymes; therefore, interactions with concomitant medications that are renally excreted or that are substrates, inducers, or inhibitors of CYP enzymes are unlikely.

References

1. Pinto D, Park Y-J, Beltramello M, Walls AC, Tortorici MA, Bianchi S, et al. Cross-neutralization of SARS-CoV-2 by a human monoclonal SARS-CoV antibody. Nature. 2020;583(7815):290-5.
2. Cathcart AL, Havenar-Daughton C, Lempp FA, Ma D, Schmid M, Agostini ML, et al. The dual function monoclonal antibodies VIR-7831 and VIR-7832 demonstrate potent in vitro and in vivo activity against SARS-CoV-2. bioRxiv. 2021.
3. Gupta A, Gonzalez-Rojas Y, Juarez E, Casal MC, Moya J, Falci DR, et al. Effect of the Neutralizing SARS-CoV-2 Antibody Sotrovimab in Preventing Progression of COVID-19: A Randomized Clinical Trial. medRxiv. 2021.
4. Wilhelm A, Widera M, Grikscheit K, Toptan T, Schenk B, Pallas C, et al. Reduced neutralization of SARS-CoV-2 omicron variant by vaccine sera and monoclonal antibodies. MedRxiv. 2021.

16.13 Fluoxetine

Rationale

Fluoxetine is a selective serotonin reuptake inhibitor introduced in the 1980s and the most globally widely used antidepressant [30]. Recently it has been classified as a Functional Inhibitor of Acid SphingoMyelinAse (FIASMA) and considered for anti-viral properties. Acid SphingoMyelinAse is a glycosome which catalyses the degradation of sphingomyelin to phosphorycholine and ceramide; ceramide causes membrane changes mediating viral uptake [31]. ASM has shown to be activated by SARS-CoV-2. FIASMA's have been demonstrated to displace ASM from lysosomal membranes, causing partial degradation, reducing the viral membrane fusion with the host cell [32].

Fluoxetine has been shown to have *in vitro* activity against SARS-CoV-2 with an EC50 of 2.6 µM demonstrated at a concentration equivalent to normal treatment for depression (0.8ug/ml) [33]. Fluoxetine has also been shown to be effective against the alpha and beta variants [34], and can be effective without cytotoxic effect [35]. *in silico* data determined that 40MG per day would provide at least 85% of patients with the trough target plasma concentrations needed to reach the target EC90 concentration within 3 days [36].

Retrospective observational tries have demonstrated the potential use of fluoxetine against COVID-19. Patients already on fluoxetine taking it within the first 48 hours of admission were found to have a lower risk of intubation or death (combined endpoint) [37]. Another study expanded this to within 10 days before or 7 days after COVID-19 diagnosis and found a reduction in death with fluoxetine [38]. Finally, a Hungarian study gave anxious patients fluoxetine on admission and found in the fluoxetine group 13.6% died compared to 30.8% not receiving fluoxetine [39].

There have been two clinical trials on another FIASMA – fluvoxamine. A double blind randomised controlled trial of 152 participants used a fluvoxamine dose of 100MG TDS for 15 days for SARS-CoV-2 non-hospitalised patients. Of the 80 participants that received fluvoxamine, none of them died, compared to 6 in the control group [40]. The TOGETHER trial used a dose of 100MG BD for 10 days, 741 received fluvoxamine and 756 received the placebo. There were 17 deaths in the fluvoxamine group compared to 25 in the placebo, p=0.24 [41]. Hospitalisation was reduced with fluvoxamine, however the endpoint defined staying in the emergency department for > 6 hours as hospitalisation, when this was removed the difference was not significant.

The ANTICOV clinical trial is proposing fluoxetine 40MG/day + inhaled budesonide for 7 days. In Silico study recommended 40 MG/OD dose with provide target plasma concentrations in 3 days.

Dose

In this study, patients will be given 40MG OD on D0 and for a further 6 days.

Pharmacokinetic characteristics

Fluoxetine is well absorbed after oral intake with a large volume of distribution. Following a single oral 40 mg dose, peak plasma concentrations of fluoxetine from 15 to 55 ng/mL are observed after 6 to 8 hours. Food does not appear to affect systemic bioavailability. Fluoxetine is metabolized extensively in the liver to norfluoxetine, which has similar pharmacological activity. The main route of elimination is hepatic, to inactive metabolites excreted by the kidney.

After acute administration, the elimination half-life of fluoxetine is 1 to 3 days and after chronic administration 4 to 6 days. Norfluoxetine is longer with an elimination half-life of 4 to

16 days after acute and chronic administration. Steady state plasma levels are attained after 4 to 5 weeks of continuous drug administration.
Pharmacokinetic sampling schedule: (only if there is evidence of accelerated viral clearance) The timings are:

- Pre-dose D0H0, then
- 1, 2, 3, 4, 6, 9, 12 and 24h post dose

Note that blood will be taken on D3 and D7 for routine haematology and biochemistry. The EDTA sample will be used to measure the drug concentrations.

Adverse effects
Common adverse effects of fluoxetine affect the gastrointestinal system (nausea, diarrhoea and anorexia) and the nervous system (insomnia, somnolence and anxiety). Care must be taken in those with existing mental health problems. Serotonin syndrome can be prevented by avoiding overuse, and should not be an issue at these doses for these durations.
Contraindications
Known hypersensitivity
Use of MAOIs and thioridazine – fluoxetine must not be given within 14 days of discontinuation, and a 5-week gap must be given before starting an MAOI or thioridazine
Other antidepressants (e.g. Selective Serotonin Reuptake Inhibitors (SSRIs)), which increase the risk of serotonin syndrome.
Cautions
Since fluoxetine is metabolised in the liver hepatic impairment may cause a prolonged half-life.
Caution should be taken with those with a pre-existing psychiatric condition.
References

1. Rossi A, Barraco A, Donda P. Fluoxetine: a review on evidence based medicine. Annals of General Hospital Psychiatry. 2004;3(1):1-8.
2. Kornhuber J, Hoertel N, Gulbins E. The acid sphingomyelinase/ceramide system in COVID-19. Molecular Psychiatry. 2021:1-8.
3. Carpinteiro A, Edwards MJ, Hoffmann M, Kochs G, Gripp B, Weigang S, et al. Pharmacological inhibition of acid sphingomyelinase prevents uptake of SARS-CoV-2 by epithelial cells. Cell Reports Medicine. 2020;1(8):100142.
4. Zimniak M, Kirschner L, Hilpert H, Seibel J, Bodem J. The serotonin reuptake inhibitor Fluoxetine inhibits SARS-CoV-2. BioRxiv. 2020.
5. Fred MS, Kuivanen S, Ugurlu H, Casarotto PC, Levanov L, Saksela K, et al. Antidepressant and antipsychotic drugs reduce viral infection by SARS-CoV-2 and fluoxetine show antiviral activity against the novel variants in vitro. bioRxiv. 2021.
6. Schloer S, Brunotte L, Goretzko J, Mecate-Zambrano A, Korthals N, Gerke V, et al. Targeting the endolysosomal host-SARS-CoV-2 interface by clinically licensed functional inhibitors of acid sphingomyelinase (FIASMA) including the antidepressant fluoxetine. Emerging microbes & infections. 2020;9(1):2245-55.
7. Eugene AR. Fluoxetine pharmacokinetics and tissue distribution suggest a possible role in reducing SARS-CoV-2 titers. F1000Research. 2021;10(477):477.
8. Hoertel N, Sánchez-Rico M, Vernet R, Beeker N, Jannot A-S, Neuraz A, et al. Association between antidepressant use and reduced risk of intubation or death in hospitalized patients with COVID-19: results from an observational study. Molecular psychiatry. 2021:1-14.
9. Oskotsky T, Marić I, Tang A, Oskotsky B, Wong RJ, Aghaeepour N, et al. Mortality Risk Among Patients With COVID-19 Prescribed Selective Serotonin Reuptake Inhibitor Antidepressants. JAMA network open. 2021;4(11):e2133090-e.

10. Németh ZK, Szûcs A, Vitrai J, Juhász D, Németh JP, Holló A. Fluoxetine use is associated with improved survival of patients with COVID-19 pneumonia: A retrospective case-control study. Ideggyogyaszati Szemle. 2021;74(11-12):389-96.
11. Lenze EJ, Mattar C, Zorumski CF, Stevens A, Schweiger J, Nicol GE, et al. Fluvoxamine vs placebo and clinical deterioration in outpatients with symptomatic COVID-19: a randomized clinical trial. Jama. 2020;324(22):2292-300.
12. Reis G, dos Santos Moreira-Silva EA, Silva DCM, Thabane L, Milagres AC, Ferreira TS, et al. Effect of early treatment with fluvoxamine on risk of emergency care and hospitalisation among patients with COVID-19: the TOGETHER randomised, platform clinical trial. The Lancet Global Health. 2021.

16.14 Fluvoxamine

Rationale

Fluvoxamine is a selective serotonin reuptake inhibitor developed in 1983 and approved by the U.S. FDA in 1994. It is used to treat major depressive disorder and Obsessive-Compulsive Disorder. Recently it has been classified as a Functional Inhibitor of Acid SphingoMyelinAse (FIASMA) and considered for anti-viral properties. Acid SphingoMyelinAse is a glycosome which catalyses the degradation of sphingomyelin to phosphorycholine and ceramide; ceramide causes membrane changes mediating viral uptake [31]. ASM has shown to be activated by SARS-CoV-2. FIASMA's have been demonstrated to displace ASM from lysosomal membranes, causing partial degradation, reducing the viral membrane fusion with the host cell [32].

A double blind randomised controlled trial of 152 participants used a fluvoxamine dose of 100MG TDS for 15 days for SARS-CoV-2 non-hospitalised patients. Of the 80 participants that received fluvoxamine, none of them died, compared to 6 in the control group [40]. The TOGETHER trial used a dose of 100MG BD for 10 days. 741 received fluvoxamine and 756 received the placebo. There were 17 deaths in the fluvoxamine group compared to 25 in the placebo, p=0.24. Hospitalisation was reduced with fluvoxamine (119 [16%] of 756 vs 79 [11%] of 741); relative risk [RR] 0·68; 95% Bayesian credible interval [95% BCI]: 0·52–0·88). However, the endpoint defined staying in the emergency department for > 6 hours as hospitalisation, when this was removed the difference was not significant [41].

A quasi-randomised trial was performed in California after COVID-19 outbreak. During mass testing, patients were given the choice of fluvoxamine therapy. 50-100 MG loading dose, then 50MG BD for 14 days, follow up at 7 and 14 days. The fluvoxamine group were 5% non-white compared to 27% no therapy group. 38% of fluvoxamine patients were asymptomatic at day 0 compared to 58% in no therapy. Hospitalisation up to day 14 was 0/65 (0%) for fluvoxamine and 6/48 (12.5%) for no therapy (P=0.005) with 2 needing mechanical ventilation and one dying. At day 14 - ongoing symptoms were present in 0% with fluvoxamine compared with 60% (29 of 48) with no therapy (P < 0.001). The most common ongoing symptom were anxiety (n = 19), however it was not shown which group was more anxious. No serious adverse events occurred with fluvoxamine [42].

Dose

In this study, patients will be given 100mg TDS starting on D0 and for a further 6 days, as tolerated.

Traditionally treatment is initiated at the lowest possible dose (50 mg) given once daily at bedtime, and then increased to 100 mg daily at bedtime after a few days, as tolerated.

Effective daily dose is usually between 100 mg and 200 mg, up to a maximum of 300 mg.

In the clinical trials listed above dosing was 100mg TDS, 100mg BD and 50mg BD (with loading dose) respectively – a large range.

Pharmacokinetic characteristics

Fluvoxamine is well absorbed after oral administration. Following a single 100 mg oral dose, peak plasma levels of 31-87 ng/mL were attained 1.5 to 8 hours post-dose. Peak plasma levels are directly proportionate to dose of single oral doses.

Following single doses, the mean plasma half-life is 15 hours, and slightly longer (17-22 hours), during repeated dosing. Steady-state plasma levels are usually achieved within 10-14 days.

Fluvoxamine undergoes extensive hepatic transformation, to at least nine metabolites, which are excreted by the kidney.

Pharmacokinetic sampling schedule: (only if there is evidence of accelerated viral clearance)

The timings are:

- Pre-dose D0H0, then
- 1, 2, 3, 4, 6, 9, 12 and 24h post dose

Note that blood will be taken on D3 and D7 for routine haematology and biochemistry. The EDTA sample will be used to measure the drug concentrations.

Adverse effects

Common adverse effects of fluvoxamine are gastrointestinal complaints (nausea, constipation, anorexia, diarrhoea and dyspepsia) and central nervous system complaints (somnolence, dry mouth, nervousness, insomnia and agitation). Care must be taken in those with existing mental health problems. Serotonin syndrome can be prevented by avoiding overuse.

Contraindications

Known hypersensitivity

Use of MAOIs and thioridazine – fluvoxamine must not be given within 14 days of discontinuation, and a 5-week gap must be given before starting an MAOI or thioridazine

Other antidepressants (e.g. Selective Serotonin Reuptake Inhibitors (SSRIs), which increase the risk of serotonin syndrome.

Cautions

Patients with hepatic or renal insufficiency should begin treatment with a low dose and becarefully monitored.

Caution should be taken with those with a pre-existing psychiatric condition

References

1. Kornhuber J, Hoertel N, Gulbins E. The acid sphingomyelinase/ceramide system in COVID-19. Molecular Psychiatry. 2021:1-8.
2. Carpinteiro A, Edwards MJ, Hoffmann M, Kochs G, Gripp B, Weigang S, et al. Pharmacological inhibition of acid sphingomyelinase prevents uptake of SARS-CoV-2 by epithelial cells. Cell Reports Medicine. 2020;1(8):100142.
3. Lenze EJ, Mattar C, Zorumski CF, Stevens A, Schweiger J, Nicol GE, et al. Fluvoxamine vs placebo and clinical deterioration in outpatients with symptomatic COVID-19: a randomized clinical trial. Jama. 2020;324(22):2292-300.
4. Reis G, dos Santos Moreira-Silva EA, Silva DCM, Thabane L, Milagres AC, Ferreira TS, et al. Effect of early treatment with fluvoxamine on risk of emergency care and hospitalisation among patients with COVID-19: the TOGETHER randomised, platform clinical trial. The Lancet Global Health. 2021.
5. Seftel D, Boulware DR, editors. Prospective cohort of fluvoxamine for early treatment of coronavirus disease 19. Open Forum Infectious Diseases; 2021: Oxford University Press US.

16.15 AZD7442 (Evusheld)

Rationale

AZD7442 is a combination of two long acting antibodies – tixagevimab and cilgavimab. They were both derived from B cells donated by patients who recovered from SARS-CoV-2. Individually they prevent the spike protein from being attached to the ACE2 receptor, together they extend the half-life and reduce effector functions. In non-human primate models, prophylactic AZD7442 prevented infection from SARS-CoV-2, and therapeutic administration caused accelerated virus clearance from lungs. A phase 1 study in healthy participants found that a 300 MG IM injection (IM injections of 150MG of tixagevimab and 150MG of cilgavimab), provided SARS-CoV-2 neutralising titres 10-fold higher than that of convalescent serum for at least 3 months. They remained 3 fold higher after 9 months [43].

A phase 3 double blind placebo-controlled study (PROVENT trial) used Evusheld as immunoprophylaxis on unvaccinated adults without prior infection. The intervention group was given a single 300 MG AZD7442 dose for prevention of symptomatic positive SARS-CoV-2 RT-PCR. Primary efficacy analysis showed that the relative risk ratio was 0.77 (0.46 – 0.90) vs placebo (P<0.001) [44].

Another Phase 3 randomised double-blind controlled trial (STORM CHASER) gave unvaccinated adults with confirmed exposure to SARS-CoV-2 (within the last 8 days) a single 300 MG AZD7442 dose. AZD7442 reduced the risk of developing symptomatic COVID-19 by 73% (27 – 90%) [45].

The TACKLE phase 3, randomised, double blind, placebo-controlled trial used a 600MG IM dose of AZD7442 (IM injections of 300MG of tixagevimab and 300MG of cilgavimab). Eligible patients were those with mild-to-moderate COVID-19 and symptomatic for no longer than seven days. Primary end point was severe COVID-19 or death. In the intervention arm there were 18 recorded events (18/407) and 37 in the placebo arm (37/415) [46].

Composition & dose

Recommended dose by the US FDA is 300MG of tixagevimab and 300 MG of cilgavimab administered as consecutive IM injections at different sites. Patients should be monitored for at least 1 hour after administration.

Pharmacokinetic characteristics

Peak plasma time is 14 days for both antibodies, peak plasma concentration is 16.5 mcg/mL for tixagevimab and 15.3 mcg/ml for cilgavimab. The half-life is 87.9 days for tixagevimab and 82.9 days for cilgavimab.

Pharmacokinetic schedule: only if there is evidence of accelerated viral clearance

As a type B intervention (positive control), intensive pharmacokinetic sampling will not be conducted on those receiving Evusheld.

Toxicity

Serious hypersensitivity reactions, including anaphylaxis, have been observed with Human immunoglobulin G1 (IgG1) monoclonal antibodies like Evusheld. If signs and symptoms of a clinically significant hypersensitivity reaction or anaphylaxis occur while taking Evusheld, immediately discontinue administration and initiate appropriate medications and/or supportive care.

Contraindications

Hypersensitivity

Cautions

A higher proportion of subjects who received Evusheld versus placebo have reported myocardial infarction and cardiac failure serious adverse events. All of these subjects had

cardiac risk factors or a previous history of cardiovascular disease, there was no clear temporal pattern. A causal relationship between Evusheld and these events has not been established.

Drug Interactions

No formal drug interaction studies have been performed with Evusheld. Tixagevimab and cilgavimab are not renally excreted or metabolized by cytochrome P450 (CYP) enzymes. Interactions with concomitant medications that are renally excreted or that are substrates, inducers, or inhibitors of CYP enzymes are unlikely.

References

1. Loo Y-M, McTamney PM, Arends RH, Abram ME, Aksyuk AA, Diallo S, et al. The SARS-CoV-2 monoclonal antibody combination, AZD7442, is protective in non-human primates and has an extended half-life in humans. Science Translational Medicine. 2022:eabl8124.
2. Levin MJ, Ustianowski A, De Wit S, Launay O, Avila M, Seegobin S, et al., editors. LB5. PROVENT: Phase 3 Study of Efficacy and Safety of AZD7442 (Tixagevimab/Cilgavimab) for Pre-exposure Prophylaxis of COVID-19 in Adults. Open Forum Infectious Diseases; 2021: Oxford University Press US.
3. CHASER S. AstraZeneca: Update on AZD7442 STORM CHASER trial in post-exposure prevention of symptomatic COVID-19.
4. Mode D, Stockholm LC. AstraZeneca: AZD7442 reduced risk of developing severe COVID-19 or death in TACKLE Phase III outpatient treatment trial.

16.16 Ensitrelvir

Rationale

Ensitrelvir (S-217622) is a 3C-like protease inhibitor developed by Shionogi & Co., Ltd.. S-217622 is an oral novel small-molecule inhibitor for 3C-like protease developed through large-scale screening and structure based optimisation [47]. Coronaviruses contain two proteases, main protease – Mpro (also known as 3CL protease – 3CLpro) and papain-like protease. They cleave nascent viral polyproteins for maturation in host cells. Because these proteases play essential roles in the intracellular amplification stage of SARS-CoV-2 and lack human homologues, they are ideal targets for specific antivirals. S-217622 showed favourable bioavailability and tolerability in healthy adults in a phase I trial, and has been evaluated in phase II/III trial.

In vivo testing was performed on intranasally infected hamsters with omicron given S-217622. There was marked reduction in lung virus titres, no virus was detected in 3 of the 4 tested hamsters at day 4 post infection. There was also a 9.9 fold reduction in viral loads in the nasal turbinates at day 4 [48].

The data from a phase 2/3 study showed promising results. Japanese patients with mild/moderate COVID-19 were given placebo or a 5-day course of oral ensitrelvir fumaric acid (dosing 375mg on day 1 followed by 125mg daily, or 750mg on day 1 followed by 250mg daily). For the phase 2a trial, the primary outcome was the change from baseline in SARS-CoV-2 viral titre. The median time to viral clearance (first negative SARS-CoV-2 viral titre) was significantly shorter compared to placebo in the ensitrelvir 125 mg group (61.3 hours; reduction of 49.8 hours; P = 0.0159) and 250 mg group (62.7 hours, reduction of 48.4 hours; P = 0.0205)).

For safety assessment, treatment-emergent adverse events (TEAEs) were assessed and reported in 11 (52.4%) of the 125mg group, 16 (69.6%) of the 250mg group, and 9 (37.5%) in the placebo. They were all mild to moderate in severity, and the most common was a decrease in HDL cholesterol [49].

The phase 2b part of the trial was performed next. Patients were randomized (1:1:1) to receive oral ensitrelvir fumaric acid at the same doses as the phase 2a trial. The co-primary efficacy endpoints were total scores for 12 COVID-19 symptoms and SARS-CoV-2 viral titre.

The mean total score of the predefined 12 COVID-19 symptoms showed a decreasing trend with time after treatment initiation in all groups. The time-weighted average change from baseline up to 120 hours was significantly greater with ensitrelvir versus placebo in the subtotal scores for acute symptoms (250mg group, P=0.0070), for main clinical symptoms (250mg group, P=0.0149) and for respiratory symptoms (125mg and 250mg groups, P=0.0153 and 0.0033, respectively). No significant difference was seen in the time to first improvement of COVID-19 symptoms.

For viral titre – day 4 change from baselines for viral titre was significantly greater with ensitrelvir 125mg and 250mg (differences from placebo: −0.41, P=<0.0001 for both).

For safety, there were overall 48 (34.3%), 60 (42.9%), and 44 (31.2%) patients in the ensitrelvir 125 mg, ensitrelvir 250 mg, and placebo groups, respectively, reporting TEAEs (most were mild). Adverse Events were observed in 19 (13.6%), 31 (22.1%), and 7 (5.0%) patients in the ensitrelvir 125 mg, ensitrelvir 250 mg, and placebo groups, respectively. The majority resolved without sequelae. As with the phase 2a trial the most frequently reported were a decrease in HDL. Other common adverse events in the test groups were headaches, diarrhoea, rash and back pain.

There were two SAEs in the placebo group only. Asymptomatic dose-dependent, transient change in the HDL cholesterol, triglyceride, total bilirubin, and iron levels was observed on day 6 in the ensitrelvir groups [50].

Composition & dose

Each tablet contains ensitrelvir fumaric acid 125mg.

Dose is three 125mg tablets (375mg) on day 1, then one 125mg tablet for a total treatment course of 5 days.

Pharmacokinetic characteristics

The geometric mean of half-life ranged from 42.2 to 48.1 hours.

Pharmacokinetic schedule: only if there is evidence of accelerated viral clearance

All patients will need to be admitted for 12 hours for intense sampling. The timings are:

- Pre-dose D0H0, then
- 1, 2, 3, 4, 6, 9, 12 and 24h

Toxicity

Asymptomatic dose-dependent, transient change in the HDL cholesterol, triglyceride, total bilirubin, and iron levels have been observed.

Contraindications

Hypersensitivity

Ensitrelvir is contraindicated with medicinal products that are highly dependent on CYP3A for clearance and for which elevated concentrations are associated with serious and/or life-threatening reactions.

- Anticancer agents: ibrutinib, venetoclax
- Antigout agents: colchicine (in the participants with hepatic or renal impairment.)
- Antihyperlipidemic agents: lomitapide
- Antiplatelet agents: ticagrelor
- Antipsychotics: lurasidone
- Antiviral agents: anamorelin
- Cardiovascular agents: ivabradine
- Ergot derivatives: ergotamine
- PDE5 inhibitors: tadalafil
- Sedative/hypnotics: suvorexant

In addition, medicinal products that are potent CYP3A inducers which are considered to decrease the plasma concentration of S-217622 significantly and may reduce virologic response.

- Antibiotics: rifampicin
- Anticancer agents: apalutamide, enzalutamide, mitotane
- Anticonvulsants: carbamazepine, phenytoin
- Herbal products: St John's wort

Cautions

There are no studies in pregnant woman. The potential risk of ensitrelvir in pregnant women is unknown, breastfeeding should be avoided.

The adverse reactions identified in clinical studies are rash, pruritus, nausea, diarrhoea, headache and dyslipidaemia.

Drug Interactions
See contraindications above.
References
1. J Q. nCoV-2019 sequencing protocol v3 (LoCost) V.3. 2020.
2. Katoh K, Misawa K, Kuma K, Miyata T. MAFFT: a novel method for rapid multiple sequence alignment based on fast Fourier transform. *Nucleic Acids Res* 2002; **30**(14): 3059-66.
3. Nextclade: clade assignment, mutation calling and quality control for viral genomes. 2021. https://www.researchgate.net/journal/The-Journal-of-Open-Source-Software-2475-9066.
4. snipit GitHub. https://github.com/aineniamh/snipit.
5. Wilm A, Aw PP, Bertrand D, et al. LoFreq: a sequence-quality aware, ultra-sensitive variant caller for uncovering cell-population heterogeneity from high-throughput sequencing datasets. *Nucleic Acids Res* 2012; **40**(22): 11189-201.
6. Bloom JD, Neher RA. Fitness effects of mutations to SARS-CoV-2 proteins. *bioRxiv* 2023: 2023.01.30.526314.
7. Interactive plots of estimated fitness effects of SARS-CoV-2 amino-acid mutations. https://jbloomlab.github.io/SARS2-mut-fitness/.
8. Index of /goldenPath/wuhCor1/UShER_SARS-CoV-2/2023/03/09. http://hgdownload.soe.ucsc.edu/goldenPath/wuhCor1/UShER_SARS-CoV-2/2023/03/09/.
9. Turakhia Y, Thornlow B, Hinrichs AS, et al. Ultrafast Sample placement on Existing tRees (UShER) enables real-time phylogenetics for the SARS-CoV-2 pandemic. *Nat Genet* 2021; **53**(6): 809-16.
10. Watson JA, Kissler SM, Day NPJ, Grad YH, White NJ. Characterizing SARS-CoV-2 Viral Clearance Kinetics to Improve the Design of Antiviral Pharmacometric Studies. *Antimicrob Agents Chemother* 2022; **66**(7): e0019222.
11. Schilling WHK, Jittamala P, Watson JA, et al. Pharmacometrics of high-dose ivermectin in early COVID-19 from an open label, randomized, controlled adaptive platform trial (PLATCOV). *Elife* 2023; **12**.
12. Lange KL LR, Taylor JMG. . Robust Statistical Modeling Using the t Distribution. *J Am Stat Assoc* 1989; **989. 84(408), 881–896**.
13. Singh AK, Singh A, Singh R, Misra A. Molnupiravir in COVID-19: A systematic review of literature. *Diabetes & Metabolic Syndrome: Clinical Research & Reviews* 2021; **15**(6): 102329.
14. Sheahan TP, Sims AC, Zhou S, et al. An orally bioavailable broad-spectrum antiviral inhibits SARS-CoV-2 and multiple endemic, epidemic and bat coronavirus. *Biorxiv* 2020.
15. Wahl A, Gralinski LE, Johnson CE, et al. SARS-CoV-2 infection is effectively treated and prevented by EIDD-2801. *Nature* 2021; **591**(7850): 451-7.
16. Painter WP, Holman W, Bush JA, et al. Human safety, tolerability, and pharmacokinetics of molnupiravir, a novel broad-spectrum oral antiviral agent with activity against SARS-CoV-2. *Antimicrobial agents and chemotherapy* 2021; **65**(5): e02428-20.
17. Khoo SH, Fitzgerald R, Fletcher T, et al. Optimal dose and safety of molnupiravir in patients with early SARS-CoV-2: a Phase I, open-label, dose-escalating, randomized controlled study. *Journal of Antimicrobial Chemotherapy* 2021; **76**(12): 3286-95.
18. Jayk Bernal A, Gomes da Silva MM, Musungaie DB, et al. Molnupiravir for oral treatment of Covid-19 in nonhospitalized patients. *New England Journal of Medicine* 2021.

19. Merck & Co. I. Merck and Ridgeback Biotherapeutics Provide Update on Results from MOVe-OUT Study of Molnupiravir, an Investigational Oral Antiviral Medicine, in At Risk Adults With Mild-to-Moderate COVID-19. 2021.
20. Boras B, Jones RM, Anson BJ, et al. Discovery of a novel inhibitor of coronavirus 3CL protease as a clinical candidate for the potential treatment of COVID-19. *BioRxiv* 2021: 2020.09. 12.293498.
21. de Vries M, Mohamed AS, Prescott RA, et al. A comparative analysis of SARS-CoV-2 antivirals characterizes 3CLpro inhibitor PF-00835231 as a potential new treatment for COVID-19. *Journal of Virology* 2021; **95**(10): e01819-20.
22. Barilli A, Visigalli R, Ferrari F, et al. Organic Cation Transporters (OCTs) in EpiAirway™, a Cellular Model of Normal Human Bronchial Epithelium. *Biomedicines* 2020; **8**(5): 127.
23. Owen DR, Allerton CM, Anderson AS, et al. An oral SARS-CoV-2 Mpro inhibitor clinical candidate for the treatment of COVID-19. *Science* 2021: eabl4784.
24. Mahase E. Covid-19: Pfizer's paxlovid is 89% effective in patients at risk of serious illness, company reports. British Medical Journal Publishing Group; 2021.
25. Tu Y, Poblete RJ, Freilich BD, Zarbin MA, Bhagat N. Retinal toxicity with ritonavir. *International journal of ophthalmology* 2016; **9**(4): 640.
26. Pinto D, Park Y-J, Beltramello M, et al. Cross-neutralization of SARS-CoV-2 by a human monoclonal SARS-CoV antibody. *Nature* 2020; **583**(7815): 290-5.
27. Cathcart AL, Havenar-Daughton C, Lempp FA, et al. The dual function monoclonal antibodies VIR-7831 and VIR-7832 demonstrate potent in vitro and in vivo activity against SARS-CoV-2. *bioRxiv* 2021.
28. Gupta A, Gonzalez-Rojas Y, Juarez E, et al. Effect of the Neutralizing SARS-CoV-2 Antibody Sotrovimab in Preventing Progression of COVID-19: A Randomized Clinical Trial. *medRxiv* 2021.
29. Wilhelm A, Widera M, Grikscheit K, et al. Reduced neutralization of SARS-CoV-2 omicron variant by vaccine sera and monoclonal antibodies. *MedRxiv* 2021.
30. Rossi A, Barraco A, Donda P. Fluoxetine: a review on evidence based medicine. *Annals of General Hospital Psychiatry* 2004; **3**(1): 1-8.
31. Kornhuber J, Hoertel N, Gulbins E. The acid sphingomyelinase/ceramide system in COVID-19. *Molecular Psychiatry* 2021: 1-8.
32. Carpinteiro A, Edwards MJ, Hoffmann M, et al. Pharmacological inhibition of acid sphingomyelinase prevents uptake of SARS-CoV-2 by epithelial cells. *Cell Reports Medicine* 2020; **1**(8): 100142.
33. Zimniak M, Kirschner L, Hilpert H, Seibel J, Bodem J. The serotonin reuptake inhibitor Fluoxetine inhibits SARS-CoV-2. *BioRxiv* 2020.
34. Fred MS, Kuivanen S, Ugurlu H, et al. Antidepressant and antipsychotic drugs reduce viral infection by SARS-CoV-2 and fluoxetine show antiviral activity against the novel variants in vitro. *bioRxiv* 2021.
35. Schloer S, Brunotte L, Goretzko J, et al. Targeting the endolysosomal host-SARS-CoV-2 interface by clinically licensed functional inhibitors of acid sphingomyelinase (FIASMA) including the antidepressant fluoxetine. *Emerging microbes & infections* 2020; **9**(1): 2245-55.
36. Eugene AR. Fluoxetine pharmacokinetics and tissue distribution suggest a possible role in reducing SARS-CoV-2 titers. *F1000Research* 2021; **10**(477): 477.

37. Hoertel N, Sánchez-Rico M, Vernet R, et al. Association between antidepressant use and reduced risk of intubation or death in hospitalized patients with COVID-19: results from an observational study. *Molecular psychiatry* 2021: 1-14.
38. Oskotsky T, Marić I, Tang A, et al. Mortality Risk Among Patients With COVID-19 Prescribed Selective Serotonin Reuptake Inhibitor Antidepressants. *JAMA network open* 2021; **4**(11): e2133090-e.
39. Németh ZK, Szûcs A, Vitrai J, Juhász D, Németh JP, Holló A. Fluoxetine use is associated with improved survival of patients with COVID-19 pneumonia: A retrospective case-control study. *Ideggyogyaszati Szemle* 2021; **74**(11-12): 389-96.
40. Lenze EJ, Mattar C, Zorumski CF, et al. Fluvoxamine vs placebo and clinical deterioration in outpatients with symptomatic COVID-19: a randomized clinical trial. *Jama* 2020; **324**(22): 2292-300.
41. Reis G, dos Santos Moreira-Silva EA, Silva DCM, et al. Effect of early treatment with fluvoxamine on risk of emergency care and hospitalisation among patients with COVID-19: the TOGETHER randomised, platform clinical trial. *The Lancet Global Health* 2021.
42. Seftel D, Boulware DR. Prospective cohort of fluvoxamine for early treatment of coronavirus disease 19. Open Forum Infectious Diseases; 2021: Oxford University Press US; 2021. p. ofab050.
43. Loo Y-M, McTamney PM, Arends RH, et al. The SARS-CoV-2 monoclonal antibody combination, AZD7442, is protective in non-human primates and has an extended half-life in humans. *Science Translational Medicine* 2022: eabl8124.
44. Levin MJ, Ustianowski A, De Wit S, et al. LB5. PROVENT: Phase 3 Study of Efficacy and Safety of AZD7442 (Tixagevimab/Cilgavimab) for Pre-exposure Prophylaxis of COVID-19 in Adults. Open Forum Infectious Diseases; 2021: Oxford University Press US; 2021. p. S810-S.
45. CHASER S. AstraZeneca: Update on AZD7442 STORM CHASER trial in post-exposure prevention of symptomatic COVID-19.
46. Mode D, Stockholm LC. AstraZeneca: AZD7442 reduced risk of developing severe COVID-19 or death in TACKLE Phase III outpatient treatment trial.
47. Unoh Y, Uehara S, Nakahara K, et al. Discovery of S-217622, a Noncovalent Oral SARS-CoV-2 3CL Protease Inhibitor Clinical Candidate for Treating COVID-19. *Journal of Medicinal Chemistry* 2022.
48. Uraki R, Kiso M, Imai M, et al. Therapeutic efficacy of antibodies and antivirals against a SARS-CoV-2 Omicron variant. 2022.
49. Mukae H, Yotsuyanagi H, Ohmagari N, et al. A Randomized Phase 2/3 Study of Ensitrelvir, a Novel Oral SARS-CoV-2 3C-like Protease Inhibitor, in Japanese Patients With Mild-to-Moderate COVID-19 or Asymptomatic SARS-CoV-2 Infection: Results of the Phase 2a Part. *medRxiv* 2022.
50. Mukae H, Yotsuyanagi H, Ohmagari N, et al. Efficacy and safety of ensitrelvir in patients with mild-to-moderate COVID-19: the phase 2b part of a randomized, placebo-controlled, phase 2/3 study. *medRxiv* 2022.

1. Unoh Y, Uehara S, Nakahara K, Nobori H, Yamatsu Y, Yamamoto S, et al. Discovery of S-217622, a Noncovalent Oral SARS-CoV-2 3CL Protease Inhibitor Clinical Candidate for Treating COVID-19. Journal of Medicinal Chemistry. 2022.
2. Uraki R, Kiso M, Imai M, Yamayoshi S, Ito M, Ujie M, et al. Therapeutic efficacy of antibodies and antivirals against a SARS-CoV-2 Omicron variant. 2022.
3. Mukae H, Yotsuyanagi H, Ohmagari N, Doi Y, Imamura T, Sonoyama T, et al. A Randomized Phase 2/3 Study of Ensitrelvir, a Novel Oral SARS-CoV-2 3C-like Protease Inhibitor, in Japanese Patients With Mild-to-Moderate COVID-19 or Asymptomatic SARS-CoV-2 Infection: Results of the Phase 2a Part. medRxiv. 2022.

4. Mukae H, Yotsuyanagi H, Ohmagari N, Doi Y, Sakaguchi H, Sonoyama T, et al. Efficacy and safety of ensitrelvir in patients with mild-to-moderate COVID-19: the phase 2b part of a randomized, placebo-controlled, phase 2/3 study. medRxiv. 2022.

17 Appendix 3 – Sample size simulations

Sample size projections were done via simulation. Each simulation used a pharmacodynamic model of viral clearance (4) that was fitted to prospectively collected viral load data in 46 individuals. For simplicity, we assumed there was no site dependent effects.

Interim analyses were performed after 50 patients were enrolled and then for every subsequent 25 patients. In the simulation, out of the 5 interventions 4 were ineffective (no increase in viral clearance) and 1 was effective (10% increase in viral clearance). Figures 7 & 8 show the type 1 and 1-type 2 errors for different choices of stopping rule thresholds, respectively.

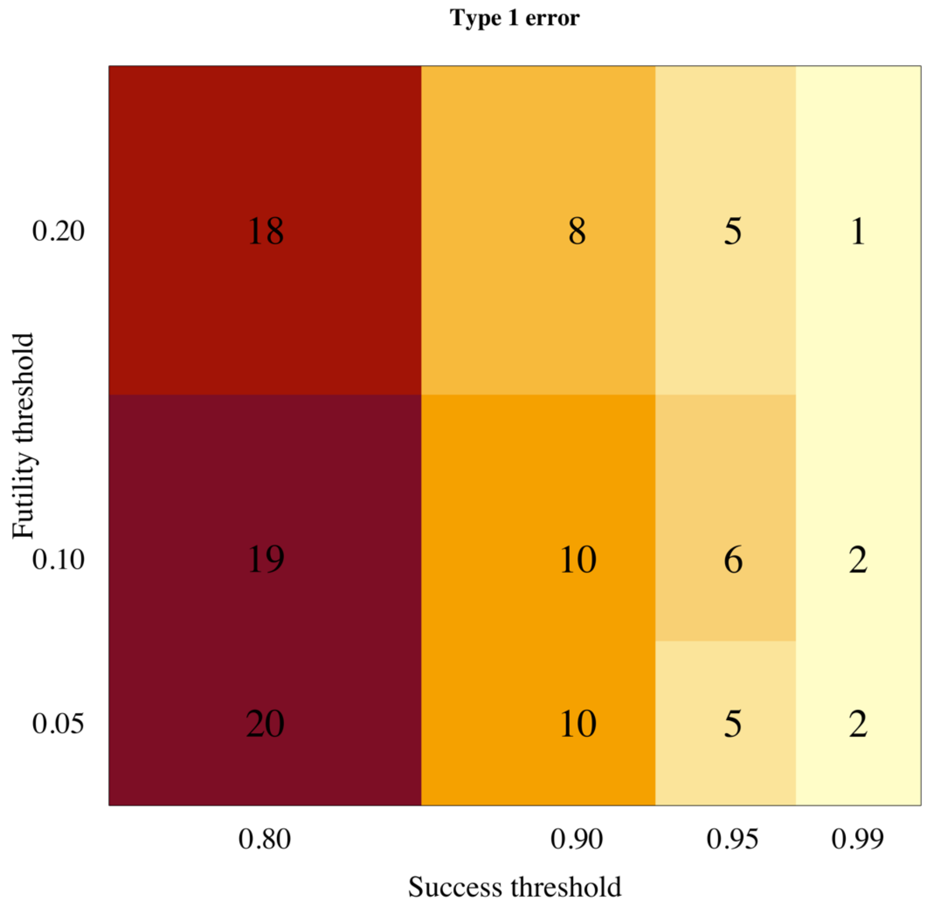

Figure 7 Estimated type 1 error

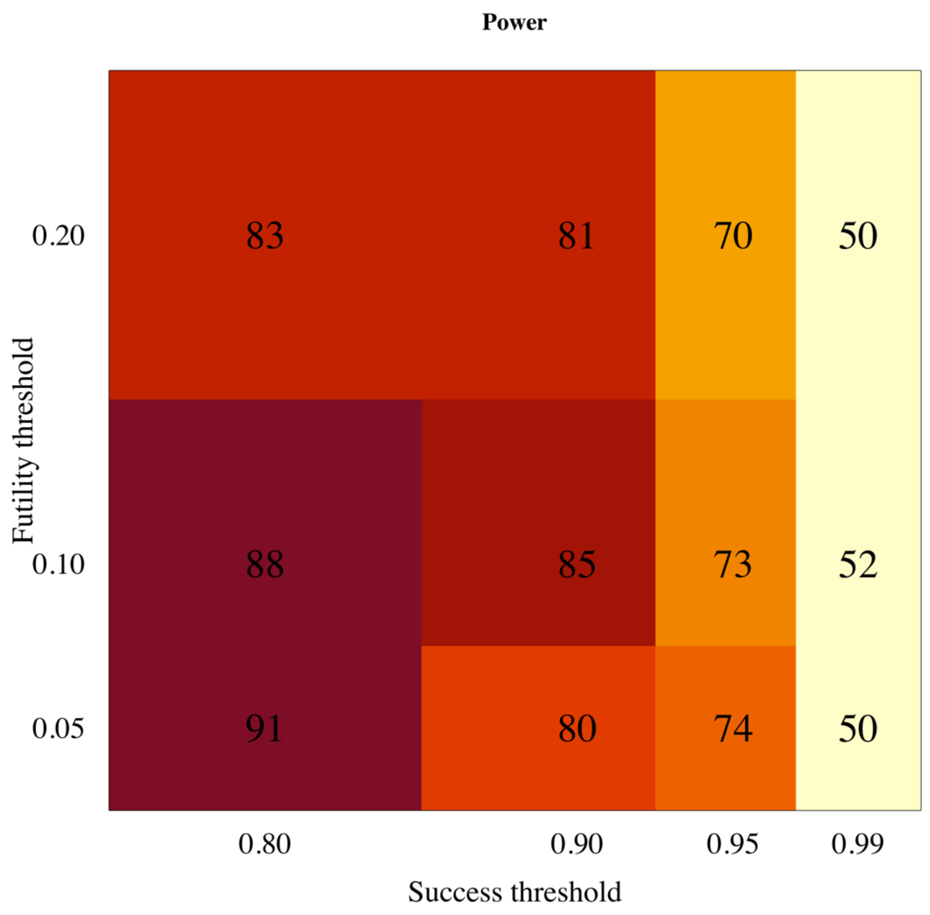

Figure 8 Estimated power (1-type 2 error)

18 Appendix 4 – Exclusion criteria

The exclusion criteria in the Master Protocol are expanded upon here. The exclusion criteria in **bold** below have a corresponding section in this appendix where more details are given. The patient may not enter the study if ANY of the following apply:

- **Taking any concomitant medications or drugs†**
- **Presence of any chronic illness/ condition requiring long term treatment, or other significant comorbidity**
- **Laboratory abnormalities discovered at screening**
- For females: pregnancy, actively trying to become pregnant, or lactation
- **Contraindication to taking, or known hypersensitivity reaction to any of the proposed therapeutics**
- Currently participating in another COVID-19 therapeutic or vaccine trial
- Evidence of pneumonia (although imaging is NOT required)

† healthy women on the oral contraceptive pill are eligible to join the study

1. Taking any concomitant medications or drugs

The study will exclude all patients taking **regular drugs** including herbal drugs for the treatment of COVID-19, but some patients may take over the counter drugs or other vitamin and herbal supplements if considered safe in the judgement of the site investigator upon screening assessment. Patients should be counseled to end and avoid all non-essential medications during study participation. In certain circumstances where a patient is on a regular medication, which does not reflect an excluded underlying illness and in the decision of the study PI is not likely to interfere with the study outcomes, or predispose the patient to any increased risk, a decision can be made to enrol. In all cases, the risks/ benefits of this decision should be documented, as well as the person(s) making the decision.
Drug interactions change rapidly as new data become available. If in any doubt, please refer to some of the web sites below:
https://www.covid19-druginteractions.org/checker

https://crediblemeds.org/

https://compendium.ch/fr/Patient - this is a Swiss website dealing with drug interactions

https://www.bnf.org/products/bnf-online/ - available only in the UK

https://www.uptodate.com/drug-interactions/?source=responsive_home#di-druglist – this requires a subscription

2. Presence of any chronic illness/ condition requiring long term treatment, or other significant comorbidity

The protocol excludes almost all individuals with a chronic illness of any severity and those with an illness that requires long term treatment. In addition, the following are considered

significant comorbidities due to their known association with worse outcomes in COVID-19 according to the MOPH guideline; 1. Chronic respiratory disease, 2. Cardiovascular disease, 3. Chronic kidney disease, 4. Stroke, 5. Obesity, 6. Cancer, 7. Diabetes.
BMI ≥ 35 kg/m^2

Diet controlled diabetes mellitus

Reduced kidney function – eGFR < 70 mls/min/1.73m^2*

Any known underlying liver disease, HIV infection and other immunocompromised condition.

Asplenia

Any underlying bleeding disorder e.g. haemophilia, von Willibrand's disease

Suffering with any other disease which in the opinion of the investigator poses undue risk to the potential patient

*eGFR for males is calculated based on the Chronic Kidney Disease Epidemiology Collaboration (Andrew S Levey, Lesley A Stevens et al. 2009)

https://qxmd.com/calculate/calculator_251/egfr-using-ckd-epi

3. Laboratory abnormalities discovered at screening

These are:

haemoglobin < 8 g/dL

platelet count < 50,000/uL

ALT > x 2 ULN

total bilirubin > 1.5 x ULN

eGFR < 70 mls/min/1.73m^2

4. Contraindication to taking, or known hypersensitivity reaction to any of the proposed therapeutics:

Where a contraindication is mentioned in the above sections, or an above contraindication incorporates drug-specific contraindications below, it is not repeated again in this section, unless further information is given which warrants some repetition. For a complete list of drug-specific contraindications please see the section for that drug in Appendix 2.

To repeat, a known hypersensitivity reaction to ANY of the proposed therapeutics below (even if the patient were to be randomised to a different treatment arm or control) is an exclusion criterion from entering into the study.

The following are relevant contraindications (i.e. in potentially eligible patients):
Hydroxychloroquine

patients with pre-existing retinopathy of the eye

patients with known hypersensitivity to any 4-aminoquinoline compounds

epilepsy

Prolonged QTc (see Hydroxychloroquine specific appendix)

Remdesivir

No additional contraindications

Ivermectin

No additional contraindications

Nitazoxanide

No additional contraindications

Miglustat

For females of childbearing age, contraception should be used throughout the study and for 2 weeks post last dose

Lopinavir/ritonavir

Concomitant use with drugs that are metabolised by CYP 3A

REGN-COV2

No additional contraindications

Nebulised Unfactionated Heparin (UFH)

- Heparin allergy or Heparin-induced thrombocytopaenia
- Known prolonged APTT > 120ms or bleeding diathesis
- Pulmonary bleeding or uncontrolled bleeding

Favipiravir

- Gout or history of gout
- For females of childbearing age, they should use effective contraception during the study and for 7 days after the last dose. For males, contraception should be used during the study and for 7 days post last dose and they should abstain from sex with a pregnant women.

Molnupiravir

As per USA FDA advice, those on molnupiravir need to be aware of avoiding pregnancy. Females of child bearing potential need to use a reliable method of contraception for the duration of the treatment, and also for 4 days after the final dose of molnupiravir. Males of reproductive potential, if they are sexually active with females of child bearing potential, should use a reliable form of contraception during the treatment and at least 3 months after the final dose

Nirmatrelvir/ritonavir

No additional contraindications

Sotrovimab

No additional contraindications

Fluoxetine

No additional contraindications

Fluvoxamine

No additional contraindications

AZD7442(Evusheld)

No additional contraindications

Ensitrelvir

No additional contraindications

Molnupiravir and Nirmatrelvir/Ritonavir combination

No interactions, no additional contraindications to individual drugs

References for exclusion criteria

Andrew S Levey, Lesley A Stevens, Christopher H Schmid, Yaping Lucy Zhang, Alejandro F Castro, Harold I Feldman, John W Kusek, Paul Eggers, Frederick Van Lente, Tom Greene and J. Coresh (2009). "A new equation to estimate glomerular filtration rate." Annals of Internal Medicine **150**: 604-612.

Williamson, E. J., A. J. Walker, K. Bhaskaran, S. Bacon, C. Bates, C. E. Morton, H. J. Curtis, A. Mehrkar, D. Evans, P. Inglesby, J. Cockburn, H. I. McDonald, B. MacKenna, L. Tomlinson, I. J. Douglas, C. T. Rentsch, R. Mathur, A. Y. S. Wong, R. Grieve, D. Harrison, H. Forbes, A. Schultze, R. Croker, J. Parry, F. Hester, S. Harper, R. Perera, S. J. W. Evans, L. Smeeth and B. Goldacre (2020). "Factors associated with COVID-19-related death using OpenSAFELY." Nature **584**(7821): 430-436.

20. Appendix 5 – Amendment History

Amendment No.	Protocol Version No.	Date issued	Author(s) of changes	Details of Changes made
1	3.0	11 July 2022	Professor Sir Nicholas J White and Dr William Schilling	1. Co-investigators changed<br>2. Sponsor address changed.<br>3. Synopsis: removed "Study Patient" row<br>4. Selection criteria changed throughout<br>5. Synopsis: Clarified in "Planned Sample Size" that no fixed sample size<br>6. Synopsis: Changed "Planned Study Period" from "2 years" to "3 years"<br>7. Synopsis: Updated "interventions" information of type A and B throughout<br>8. Synopsis: Updated "Control" throughout<br>9. Synopsis: Updated "Rational" throughout<br>10. Objectives and/or endpoint wording changed throughout<br>11. Section "3 Background and rationale": Updated information of small molecule medications and updated to reflect current global vaccine and COVID-19 situation including omicron variant.<br>12. Section "3.1 Proposal": Emphasis of why this type of trial is most suited and clarification of the expected outcome of the interim analysis.<br>13. Section "5 Study design": Updated information about the places to recruit the participants, site and current study design.<br>14. Section "5.1 Interventions": Pick the winner no longer applies, language changed to reflect addition of small molecule drugs and updated Figure 4<br>15. Section "7 Study set-up and procedures": Updated study procedures. Swabs will be done in duplicate before receiving the first dose of the intervention (or if on no intervention arm).<br>16. Section "7.1 Virological sampling": Clarification that duplicate means one swab on each tonsil, increased viral transport medium (VTM), updated the laboratory procedures

				and added saliva collection process in some participants<br>17. Section “7.5 Baseline assessments”: if the results haven’t returned the participant can be given the first dose then excluded later if there are laboratory abnormalities. The procedures are also updated.<br>18. Section “7.6.1 Randomisation D0”: Updated the ratio of randomization as no antiviral treatment will be fixed at “at least 20%” throughout the study and the randomization ratios will be uniform for all available interventions.<br>19. Section “7.6.2 Day 1 - day 7”: Updated the procedures during day 1- day 7<br>20. Section “7.7 Intensive pharmacokinetic sampling”: Explained more details about the study drugs for intensive PK sampling.<br>21. Section “7.8 Management of patients who become ill”: If treatment arm is changed, duplicate swabs will be taken again prior to any new treatment.<br>22. Section “9.1 Definition/Serious Adverse Event (SAE)”: Removed “and not otherwise receiving medical treatment”<br>23. Section “10.1 Overview of adaptive study design and overall approach”: Added new information.<br>24. Section “10.2 Results of the first interim analysis”: This new section and Figure 6 have been added.<br>25. Section “10.3 Bayesian hierarchical model of viral clearance”: Statistical explanation has been updated.<br>26. Section “Estimating the optimal structural model for clearance rate”: Deleted. No-longer required as the methodology has been validated.<br>27. Section “10.5 Sample size estimation, randomisation and statistical considerations for the Bayesian group sequential design”: Information have been updated.

				28. Section "10.7 Final analysis of primary outcome": Linear and non-linear models are being used, package *rstan* is being used instead of *rstanarm*. Analysis code available from github, link was given.<br>29. Section "13 Ethical and regulatory considerations": Clarification of what treatment will be given to no antiviral treatment arm.<br>30. Section "13.7 Benefits": Maximum amount per patient (£400 GBP) has been removed.<br>31. Section "14 References": References have been updated.<br>32. Section "15 Appendix 1 Schedule of activities": "If new treatment" column and explanation below the table have been added/updated.<br>33. Section "16 Appendix 2 Study drugs":<br>• Hydroxychloroquine, lopinavir/ritonavir, miglustat, nitazoxanide and nebulized unfractionated heparin (UFH): Reference order updated.<br>• Remdesivir: Changed for more up to date information with more references.<br>• Ivermectin: Dosing table changed for higher mg of tablets and reference order updated.<br>• REGN-COV2: Dose changed from 1,200 mg to 600 mg of casirivimab and 1,200 mg to 600 mg of imdevimab and reference order updated.<br>• Favipiravir: Introduction changed for more up to date information including references.<br>• Molnupiravir, nirmatrelvir/ritonavir (PAXLOVID™), sotrovimab, fluoxetine, fluvoxamine and AZD7442 (evusheld): Monograph added (Section 16.10 – 16.15).<br>34. Section "18 Appendix 4 Exclusion criteria":<br>• Update information to comply with the exclusion criteria<br>• Added line to include herbal drugs in regular drugs<br>• Added line of the decision of the study PI whether to enrol participants taking regular medications.

				• Add in the list of the most severe comorbidities, and include HIV and other immunocompromised condition.<br>• Changed the BMI from ≥ 30 to ≥ 35 kg/m^2.<br>• Changed the eGFR limit of exclusion from <80 to <70 mls/min/1.73m^2<br>• Added contraindications for hydroxychloroquine (prolong QTc), molnupiravir, nirmatrelvir/ritonavir (PAXLOVID™), sotrovimab, fluoxetine, fluvoxamine and AZD7442 (evusheld).<br>• Added that study may be done as an inpatient or an outpatient throughout.<br>35. Administrative changes
2	4.0	17 August 2022	Professor Sir Nicholas J White and Dr William Schilling	1. Funder: Update funder name including grant reference number<br>2. Section "1: Synopsis": Updated "Planned Sample Size" by adding expected total participant and study countries<br>3. Section "1: Synopsis": Updated "interventions" information of type A<br>4. Section "3 Background and rationale": Added information about 'viral rebound' and clarify more about assessment<br>5. Section "5.1 Interventions": Added "Ensitrelvir and a combination of Molnupiravir and Nirmatrelvir/Ritonavir" in type A and "Evusheld" in type B<br>6. Section "7.6 Days of Study": Added Day 10 activities to capture asymptomatic viral rebound and safety concern for viral rebound<br>7. Section "7.8 Management of patients who become ill": Added new procedure to determine the viral rebound which is important with certain antivirals (e.g. Paxlovid) and may impact effectiveness of these against onward viral transmission.<br>8. Section "9.3 Procedures for recording adverse events": Added safety evaluation for combination drugs<br>9. Section "10.5 Sample size estimation, randomisation and statistical considerations for the Bayesian group sequential design": Added tentative

				sample size (60 patients) in each arm for an effect size of 12.5% increase in viral clearance<br>10. Section "11.2 Data handling and record keeping": Extended the retention of the records to "at least five years"<br>11. Section "15 Appendix 1 Schedule of activities":<br>• Added column "D10"<br>• Changed column title from "If new treatment" to "if new treatment/symptoms"<br>• Clarified the definition of "if new treatment/symptoms"<br>12. Section "16 Appendix 2 Study drugs":<br>• Molnupiravir: Updated section "drug interaction"<br>• Nirmatrelvir/ritonavir (e.g. PAXLOVID™): Updated section "drug interaction"<br>• Ensitrelvir: Added monograph (Section 16.16).<br>13. Section "18 Appendix 4 Exclusion criteria": Added contraindications for ensitrelvir and molnupiravir and nirmatrelvir/ritonavir combination<br>14. Administrative changes

Finding Treatments for COVID-19: A Trial of Antiviral Pharmacodynamics in Early Symptomatic COVID-19 (PLATCOV): Statistical Analysis Plan

January 12, 2023

Registered at clinicaltrials.gov number **NCT05041907**

Version 3.0; refers to Master Protocol version V0.1

Written by:
Dr James Watson (Statistician)
Signature: Date: 12 January 2023

Reviewed by and approved by:

Prof Sir Nicholas White (Co-PI)
Signature: Date: 12 January 2023

Dr William Schilling (Co-PI)
Signature: Date: 13 January 2023

Contents

1 **Summary of changes since version 1** 3

2 **Trial Overview** 4
- 2.1 Main research questions 4

3 **Study Methods** 5
- 3.1 Trial design 5
- 3.2 Randomisation 5
  - 3.2.1 Sample size projections 5
  - 3.2.2 Sample size: before trial commencement 6
  - 3.2.3 Updated sample size calculations 6
- 3.3 Framework 7

4 **Statistical interim analyses and stopping rules** 7
- 4.1 Frequency and timing of the interim analyses 7
- 4.2 Interim analysis decision making 8
- 4.3 Timing of final analysis 8
- 4.4 Timing of outcome assessments 8

5 **Statistical Principles** 9
- 5.1 Posterior estimates 9
- 5.2 Adherence and protocol deviations 9
- 5.3 Analysis populations 9

6 **Trial population** 9
- 6.1 Screening data 9
- 6.2 Eligibility 9
- 6.3 Withdrawal and follow-up 10
- 6.4 Baseline patient characteristics 10

7 **Analysis** 11
- 7.1 Outcome definitions 11
  - 7.1.1 Viral load quantification 11
- 7.2 Analysis Methods 11
  - 7.2.1 Statistical model for the analysis of the serial viral load data 11
  - 7.2.2 Covariate adjustment 12
  - 7.2.3 Resolution of fever and symptoms 13
- 7.3 Pharmacokinetic analyses 13
- 7.4 Subgroup analyses 13
- 7.5 Prior distributions 13
- 7.6 Sensitivity analyses 14
- 7.7 Missing data 14
- 7.8 Harms 15
- 7.9 Statistical software and analysis implementation 15

S1 **Updated sample size simulations** 16

S2 **Casirivimab/imdevimab** 16

S3 **Tixagevimab/cilgavimab** 16

1 Summary of changes since version 1

Version 2.0 The first interim analysis was triggered on the 2nd of March 2022 when qPCR data from the first 50 patients was received. Following the first interim analysis, the Trial Steering Committee and the Data Safety and Monitoring Board recommended changing the minimum efficacy threshold from a 5% increase in viral clearance to a 12.5% increase. This would result in a lower number of patients required to make a futility decision and a larger number of patients required to make a success decision for treatments with no antiviral effects and antiviral effects similar to those estimated from published data on molnupiravir, respectively.

We also changed the primary analysis from modelling the viral load on the CT scale with batch effects to modelling the viral load on the log copies per mL scale, with standard curve transformation done using control samples from each batch. The interim analysis suggested this was more computationally stable and easier to interpret.

Version 2.1 We have added a section regarding the primary analysis of the pharmacokinetic data gathered from all patients.

Version 2.2 We have added a section regarding the analysis of the time to resolution of fever, time to resolution of symptoms, and proportion with normal lymphocyte counts. We updated the current status of the trial (interventions being randomised and the current positive control).

Version 3.0 Version 3 reflects a major update to the trial design and analysis. The following major changes were made:

- We have added a non-inferiority comparison relative to the positive control. Interventions which meet the success criteria are then entered into a non-inferiority comparison with the positive control using a pre-defined margin of -10%. Stopping occurs for inferiority (probability greater than 0.9 that the effect is less than the margin) or non-inferiority (probability greater than 0.9 that the effect is above the margin).
- We have increased the margin for futility/success for the comparison with the negative control (no study drug) from 12.5% to 20%. This results in smaller sample sizes required to show futility for interventions which have no effect. This reflects the updated trial aims which is to characterise effective antivirals and compare them with the 'gold-standard' antiviral treatments.
- We have changed the schedule of interim analyses (now done by arm, specified as every additional 10 patients and 10 controls recruited).

Minor changes are:

- We have removed the rapid serology test from the set of covariates used in the covariate-adjusted model. By May 2022 nearly all patients in Thailand were testing positive, in addition the Brazil site did not use these tests.
- We have updated the variant-specific subgroup analysis for the casirivimab/imdevimab monoconal antibody arm; and we have added a section on the variant-specific subgroup analysis for the tixagevimab/cilgavimab monoclonal antibidy arm.

2 Trial Overview

An effective, well tolerated, safe, affordable and generally available treatment of COVID-19 that prevented progression to severe disease would be of enormous global health benefit. There are many potential antiviral therapeutics for COVID-19, mainly consisting of repurposed small molecule drugs. At time of writing, however, there is strong evidence for clinical benefit only for the monoclonal antibodies (notably the monoclonal casirivimab/imdevimab in pre-Omicron SARS-CoV-2 variants [1]) and the small molecule drugs remdesivir [2], molnupiravir [3], and nirmatrelvir [4]. The clinical benefits of these interventions were determined from large phase 3 studies in high-risk individuals enrolled shortly after the onset of a symptomatic SARS-CoV-2 infection. The primary endpoint was most commonly either hospitalisation or death from COVID-19 (usually less than a 10% event rate). As vaccine availability has become widespread in addition to high levels of natural immunity from infection, conducting these types of clinical trials is more and more difficult as the event rates for the primary endpoints become increasingly low (e.g. the PANORAMIC trial where the event rate for the molnupiravir comparison was 0.8% [5]). This calls for improved study designs which allow for the assessment of antiviral interventions in an efficient manner (hundreds rather than tens of thousands of patients). Currently there is no consensus pharmacometric methodology to determine which antivirals should be prioritised for large phase 3 and 4 evaluation, and how to compare current candidates [6].

PLATCOV is a multi-country platform adaptive trial which will develop and validate a methodology for the quantitative assessment of antiviral effects in low-risk patients with high viral burdens and uncomplicated COVID-19. We choose patients with a low risk of progression as this justifies a no treatment arm; we target patients with high viral loads as this is a subgroup in whom antiviral effects can be detected more easily. We have already evaluated and stopped ivermectin [7], remdesivir [8], the monoclonal casirivimab/imdevimab [8], and favipiravir (unpublished).

This statistical analysis plan (SAP) covers analyses for both the interim and the final reports. We developed the SAP following the Guidelines for the Content of Statistical Analysis Plans in Clinical Trials [9]. It includes pre-specified decision rules for continuing or stopping individual trial arms based on effectiveness or futility.

2.1 Main research questions

Each clinical site in this platform trial will test multiple interventions simultaneously (depending on local regulations and drug availability). There are two control arms: (i) no study drug (negative control), this consists of no intervention other than antipyretics; (ii) nirmatrelvir/ritonavir (positive control), this can change in the future depending on pre-specified rules. Interventions of two types will be compared with the two control arms:

- Small molecule drugs: either novel or newly available or repurposed antiviral drugs (currently randomizing patients to nitazoxanide, fluoxetine, molnupiravir)
- Monoclonal antibodies: currently tixagevimab/cilgavimab.

There are distinct primary objectives for each intervention type. For the newly available antivirals and repurposed drugs, we want to characterise their antiviral activity by comparing viral clearance dynamics with no treatment. Many of these drugs are already used and recommended in some countries. Showing that they do not have clinically significant antiviral activity is as important as showing that they do. For interventions that do have a clear measurable antiviral activity, we will then compare their activity to the current gold-standard antiviral treatment (since August 2022 this is nirmatrelvir/ritonavir). This will be a non-inferiority comparison using a pre-specified threshold.

For monoclonal antibodies included on the platform, we are primarily interested in tracking their performance over time with respect to new SARS-CoV-2 variants (comparison with the no treatment group).

The secondary objectives of this study are:

- To characterise the determinants of viral clearance in early symptomatic SARS-CoV-2 (e.g. estimate the contribution of age, baseline serology, virus genotype, and prior vaccination);
- To determine optimal dosing regimens for drugs shown to have considerable antiviral activity (where deemed feasible a pharmacokinetic-pharmacodynamic sub-study will be performed);

- To compare fever clearance time and time to symptom resolution with respect to no treatment for interventions that are shown to have a measurable antiviral effect.

The tertiary objective of this study is to characterise the relationship between viral clearance and the risk of subsequent hospitalisation or death by day 28. However, the event rate in the enrolled population is likely to be extremely low (we hope 0%, thus far it has been 0 out of >700 patient enrolled) so it is very unlikely that we will be able to demonstrate any link between viral clearance and progression to severe COVID-19.

3 Study Methods

3.1 Trial design

This is a multi-centre, multi-country, open label, randomised, controlled, adaptive platform trial of antiviral interventions in early symptomatic SARS-CoV-2. There are two distinct control arms. The negative control consists of no study drug other than antipyretic; the positive control is nirmatrelvir/ritonavir (this can change if better molecules are identified). Interventions currently included in the platform are: nitazoxanide, fluoxetine, tixagevimab/cilgavimab, molnupiravir. We stopped randomisation to ivermectin (futility) in April 2022 [7]; remdesivir (success) in June 2022; casirivimab/imdevimab (no more drug) in October 2022; favipiravir (futility) in October 2022. Nirmatrelvir/ritonavir became the positive control in August 2022 when it met the success stopping rule with evidence of very strong antiviral activity. At each site there is equal allocation to all interventions but with a minimum of 20% of patients randomised to the negative control arm.

3.2 Randomisation

Randomisation is performed via a centralised web-app designed by MORU software engineers using RShiny, hosted on a MORU server. Each study nurse responsible for randomising patients has unique login credentials provided by email from the main study statistician (James Watson). Randomisation sheets are pre-generated for each study site separately using blocks of size K (where K is equal to 3 times the number of interventions available at the site) with an additional 100/K% fuzziness (randomly interchanging one of the allocations per block to avoid the study nurse knowing exactly what the Kth allocation will be).

As of December 2022, we will do factorial randomisation for the small molecule drugs and the monoclonal antibodies (in sites where the monoclonals are available).

Randomisation sheets and randomisation event logs are stored on a secure Dropbox folder (professional version that has full version control) which is accessed directly by the RShiny app. Only the study statistician James Watson and the MORU IT manager have read/write privileges to this Dropbox folder. For cross checking purposes, the randomisation app also records the patient age and sex. Each randomisation event is logged with the corresponding time-stamp and the identity of the nurse who performed the randomisation. Each time the set of interventions available at a given study site changes, a new randomisation sheet is generated, overwriting the previous one.

3.2.1 Sample size projections

The sample size is adaptive (there is no fixed sample size). For each intervention the final sample size is based pre-specified stopping rules which use a futility/success margin λ_1 for comparisons with the no study drug arm, and a non-inferiority margin λ_2 for comparisons with the positive control arm. In addition, because there are theoretical boundary cases whereby the sample size needs to be very large in order to meet the stopping rule (when the effect is exactly equal to the margin), there is a maximum sample size of 120 patients per intervention arm. An intervention arm is stopped before 120 either (i) because it meets the futility criteria (relative to the no study drug arm, defined as Probability[effect $< \lambda_1$]>0.9); or (ii) because it both meets the success criteria (relative to the no study drug arm, defined as Probability[effect $> \lambda_1$]>0.9) and a non-inferiority criteria or an inferiority criteria (relative to the positive control arm, defined as Probability[effect $>$ -λ_2]>0.9 or Probability[effect<-λ_2]>0.9, respectively). As of version 3 of the analysis plan, stopping for success requires both meeting the success rule relative to the no study drug arm and concluding

either non-inferiority or inferiority relative to the positive control arm, with comparisons done in that order.

The average sample sizes thus depend on the values of λ_1 and λ_2. Given the model-dependent nature of the analysis, simulation is required to estimate the expected distribution of sample sizes for varying effect sizes. The following sections outline how values for λ_1 (and now λ_2) were selected and changed over the course of the study.

3.2.2 Sample size: before trial commencement

Our previous simulation approach attempted to set a futility and success margin (along with a probability threshold) that would result in control of the type 1 error at approximately 10% and control of the type 2 error at approximately 20% (see study protocol for exact details). The simulations assumed that the decline in viral loads was log-linear (linear on the cycle threshold (CT) scale), using measurement error estimated from an open access database of prospectively followed individuals with frequent viral load measurement [10, 6]. We calibrated plausible effect sizes using preliminary data from the casirivimab/imdevimab phase 2 studies (this suggested increases of approximately 20% in the slope of the viral clearance on the CT scale) [11]. The first interim analysis at 50 patients suggested that these simulations had underestimated inter-individual variability in viral clearance, and had underestimated plausible antiviral effect sizes. We changed the futility/success threshold from 5% to 12.5% in response.

3.2.3 Updated sample size calculations

In November 2022 we re-ran a series of simulations to re-assess sample size requirements and optimal stopping rule thresholds using the data from the first 500 patients enrolled in the PLAT-COV trial. The simulations assessed thresholds $\lambda_1 \in \{1.125, 1.15, 1.2\}$ (i.e. 12.5%, 15% or 20%) for the futility/success assessments relative to the no study drug arm; and thresholds $\lambda_2 \in \{0.905, 0.875, 0.85\}$ (i.e. -9.5%, -12.5%, or -15%) for the non-inferiority assessments relative to the positive control arm. We used the linear model fit to the first 500 patients to simulate trial data. 100 iterations were done for each permutation of λ_1, λ_2 and for three hypothesised effect sizes (0, 40% and 60% increase relative to no study drug), i.e. 2700 simulations in total. Preliminary simulations showed that most false positive or false negative results (stopping early for futility an intervention that has effect of 40 or 60%, or stopping early for success an intervention that has an effect of 0%) occurred for interim analyses done on 10 patients per arm. For this reason, we updated the interim analysis plan so that the first interim analysis is triggered at 20 patients per arm, with subsequent analyses for each additional 10 per arm. For the non-inferiority comparisons, they start when there are at least 40 patients per arm, and only if the success stopping rule has been met (Figure 1). Each model fit only used simulated data from the intervention and the relevant control arm (the negative control for the futility/success stopping rules, the positive control for the non-inferiority stopping rules).

Choosing optimal λ_1 The results of the simulations for the success and futility stopping rules are shown in Supplementary Figures S1-S2. Comparable results were obtained as for the previous sample size simulations [6]. For $\lambda_1 = 1.125$ (12.5%) and an intervention that had no effect, the median sample size was 40 patients per arm with a 4% false positive results and 28% of inconclusive trials at 120 patients per arm. Increasing λ_1 to 1.2 (20%) would reduce the median sample size to 30 patients per arm and result in approximately 1% false positive results and 18% of inconclusive trials at 120 patients per arm.

For an intervention with an effect of 40% (i.e similar to remdesivir in our trial [8]), the median sample size was 30 patients per arm for a threshold of 12.5% and 50 patients per arm for a threshold of 20%. In all cases, false negative results were less than 3% of simulations, and inconclusive results went from 14% for a threshold of 12.5% to 34% for a threshold of 20%.

For an intervention with an effect of 60% (i.e similar to nirmatrelvir/ritonavir in our trial), the median sample size was 20 patients per arm, regardless of the λ_1 threshold. False negative results were less than 1% of simulations, and inconclusive results went from 1% for a threshold of 12.5% to 9% for a threshold of 20%.

Following discussion with the trial steering committee, it was decided to increase the λ_1 threshold to 20% in order to stop poorly performing arms quickly (this reflects how the priorities of the trial have changed as the pandemic has progressed).

Choosing optimal λ_2 The results of the simulations for the non-inferiority stopping rules are shown in Supplementary Figures S3-S4.

In simulations which met the stopping rule for success (Probability[effect>λ_1]>0.9), stopping for non-inferiority or inferiority was then assessed for each of the three threshold values. Only in 1 simulation with an effect of 0% was the non-inferiority threshold met (<0.3% false positive result).

An effect of 40% relative to no study drug arm is very close to/on the boundaries defined by the λ_2 values, assuming that the positive control has an effect of 60% relative to the no study drug. For all three boundary values, around half the simulations met the non-inferiority criterion, 10% were inconclusive and 40% met the inferiority criterion.

For an effect of 60% relative to the no study drug arm (i.e. same as the positive control), 70% to 78% of simulations met the non-inferiority criterion for the -9.5% (i.e. most stringent) to the -15% (least stringent) thresholds, respectively. Around 6% of simulations were inconclusive at 120 patients, and 24 to 17% resulted in a false inferiority result for the -9.5% (i.e. most stringent) to the -15% (least stringent) thresholds, respectively.

Following discussion with the trial steering committee, it was decided to initially set the λ_2 threshold at -10%. This results in faster stopping of interventions clearly less effective than the positive control and longer stopping for interventions with an effect close to the positive control.

3.3 Framework

All primary analyses and stopping decisions will be made using models including data from **contemporaneous controls only**. As this is an adaptive trial, temporal drift (due to mutations in the virus, changes in the patient characteristics, etc.) can confound comparisons with non-contemporaneous controls. However, we will also fit models to the full data set (using all controls), with additional covariate factors for the different trial epochs.

All primary comparisons for futility/success will be made with respect to the negative control arm; all primary comparisons for non-inferiority will be made with respect to the positive control arm (no "across intervention" arm comparisons in the primary analysis). In the case of a site not randomising patients to the negative control or the positive control (because of local objection or availability), that site will only provide indirect data to support the treatment estimates (under the hierarchical model structure). All decisions concerning efficacy will be based on super-superiority relative to the negative control arm (λ_1) whereby the probability of an effect is defined under the model as the posterior probability that the increase in viral clearance relative to the control arm is greater than λ_1 (20% from now on).

For interventions which show a measurable antiviral effect (i.e. meet the stopping rule for success), we will then compare their antiviral effect to the positive control arm (currently nirmatrelvir/ritonavir), using the non-inferiority threshold of -10%. (λ_2)

4 Statistical interim analyses and stopping rules

We plan frequent interim analyses (Figure 1). This is to allow for near-real time monitoring of accrued data in order to detect possible issues in patient recruitment, viral load swabbing, or with the PCR assays at the different sites. The stringent margin for success (greater than 20% increase) results in a very low type 1 error. In addition, meeting the success threshold triggers a subsequent non-inferiority comparison with the positive control arm and where deemed appropriate an intensive PK-PD sub-study to determine dose-response effects thus gathering additional efficacy data for that intervention. If there is strong *a priori* evidence of antiviral efficacy (e.g. molnupiravir, nirmatrelvir) additional dense blood samples for population PK-PD modelling will be obtained from enrolled patients willing to have daily blood sampling (all patients in the study have blood samples taken at baseline, day 3 and day 7 trough levels, and day 14).

4.1 Frequency and timing of the interim analyses

Decision making analyses will be done by arm (only using the data from that arm and the concurrent negative or positive controls). From version 3 of the SAP, the first interim analysis for a given arm will be performed when a minimum of 20 patients randomised to that arm (and 20 concurrent negative controls) have available and analysable qPCR data (PCR data will lag behind

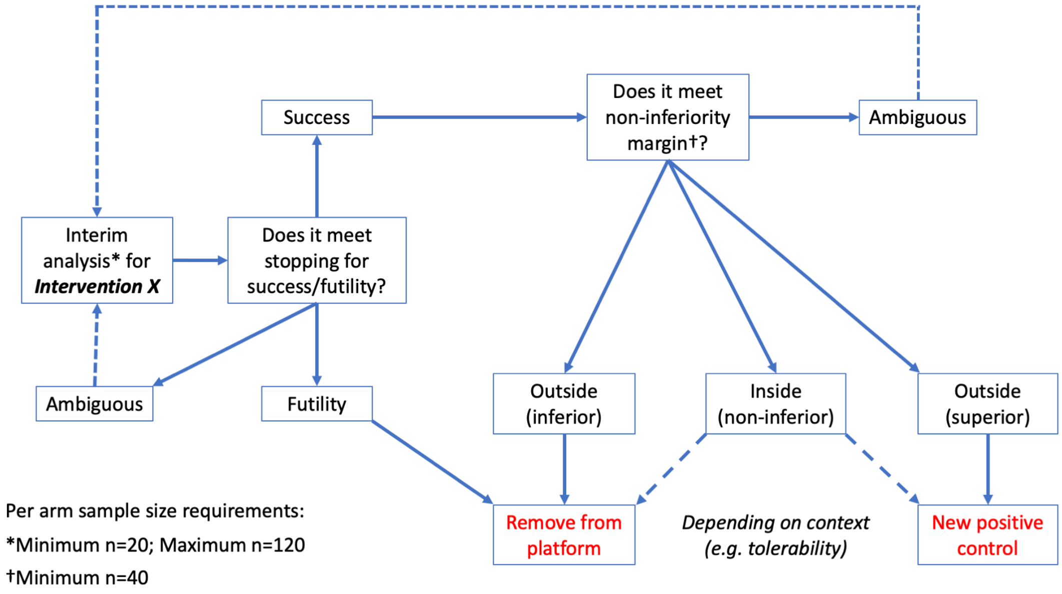

Figure 1: Planned interim analyses and decision rules for removing an arm from the platform or making an arm the new positive control.

actual recruitment, usually by approximately 3 weeks). Subsequent analyses will be performed for every additional 10 patients (10 randomised to the intervention and 10 to the control arm).

4.2 Interim analysis decision making

Figure 1 shows the decision algorithm for removing an intervention from the platform or replacing the positive control (current nirmatrelvir/ritonavir). Only the study statistician (James Watson) will be unblinded to all interim analyses. Each interim analysis report will sent to the Data Safety and Monitoring Board (DSMB) along with a summary of whether the futility or success thresholds for any of the interventions have been met. The DSMB will then make recommendations for continuing or stopping the intervention arms. Concurrent data from other trials (e.g. safety data) may be used in making these decisions. If an arm is stopped for futility or an intensive PK-PD study is triggered, then all the data pertaining to the negative control arm and the intervention arm will be presented to the Trial Steering Committee (TSC), along with the DSMB recommendations. The interim analysis of the first 50 patients with available qPCR data was presented unblinded to the TSC in order to determine necessary changes to the statistical analysis plan.

4.3 Timing of final analysis

As this is a platform trial, no overall final analysis is planned. For each intervention studied, we define the "final analysis" as the point when the last patient randomized to that arm passes their day 28 follow-up.

4.4 Timing of outcome assessments

For the primary and secondary endpoints we will use all viral load measurements taken up until day 7. Time will be defined as time since randomisation (units of days, including all timepoints < 8 days since randomisation as there will be some variation in exact clock times of the follow-up swabs).

For fever and symptom resolution, time to resolution will be calculated from daily measurements taken over the first week with right censoring for unobserved events (withdrawal or no resolution by 7 days).

The tertiary endpoint (all cause hospitalisation) will be taken up until day 28.

5 Statistical Principles

5.1 Posterior estimates

Treatment effects will be estimated under a Bayesian framework using a hierarchical model with weakly informative prior distributions. For the hyperparameters, the prior distributions are chosen for computational reasons to aid model convergence. For the key population level parameters, we will use priors based on analysis of open access data [6]. The prior on the treatment effect is driven by plausible maximum effect sizes given published data on viral decreases in patients randomised to casirivimab/imdevimab or placebo [1].

5.2 Adherence and protocol deviations

This study is open label and thus it is possible that some patients who are randomized to the no study drug arm, or to study interventions perceived to be ineffective, may be given alternative rescue treatment if their symptoms persist and the treating physician is worried that they will deteriorate clinically. This can introduce confounding between the treatment allocation and outcome. Notably, if multiple patients randomized to the negative control were in fact given the positive control before day 7, this could impact the viral loads during follow-up and thus bias the estimates of the rate of viral clearance in the negative control arm. For all protocol deviations regarding treatment (either stopping treatment for safety reasons or switching treatment arms), we will consider all viral load measurements taken after the deviation as censored, i.e. we will estimate viral load clearance rates only using the data during the period of protocol compliance. We will summarise the number of treatment protocol deviations by site.

5.3 Analysis populations

The primary analysis will be in a modified intention-to-treat (mITT) population, including follow-up data only from the period of treatment adherence. A minimum of 3 days of follow-up data are necessary in order to be included in the mITT population (e.g. patients who discontinue before day 2 will not be included).

An optional secondary analysis can be performed in the per-protocol population, defined as patients who had 100% adherence to the allocated intervention arm. Specific reasons for treatment discontinuation will be outlined and recorded in order to assess confounding bias. This will only be done in specific cases where there is a good reason for discontinuation unrelated to antiviral effect (i.e. side-effect such as gastrointestinal issues as seen with high dose nitazoxanide).

The safety population will include all patients who have received at least one dose of the intervention.

6 Trial population

Figure 2 shows an example CONSORT diagram summarising the number of patients screened; the reasons for exclusion; the number of enrolled patients randomized to each arm; and the number of patients who complete treatment and follow-up and who are included in the modified intention to treat analysis (mITT).

6.1 Screening data

We will summarise the reasons for exclusion for the screened patients not eligible for enrolment. This will not be done for the interim analysis, only for the final analysis once an arm is stopped for futility or success.

6.2 Eligibility

The key trial eligibility criteria are as follows:

- Previously healthy adults, aged 18 to 50 years (low risk of developing severe COVID-19);
- SARS-CoV-2 positive by lateral flow antigen test OR a positive PCR test for SARS-CoV-2 within the last 24 hours with a CT value of less than 25 (all viral targets);

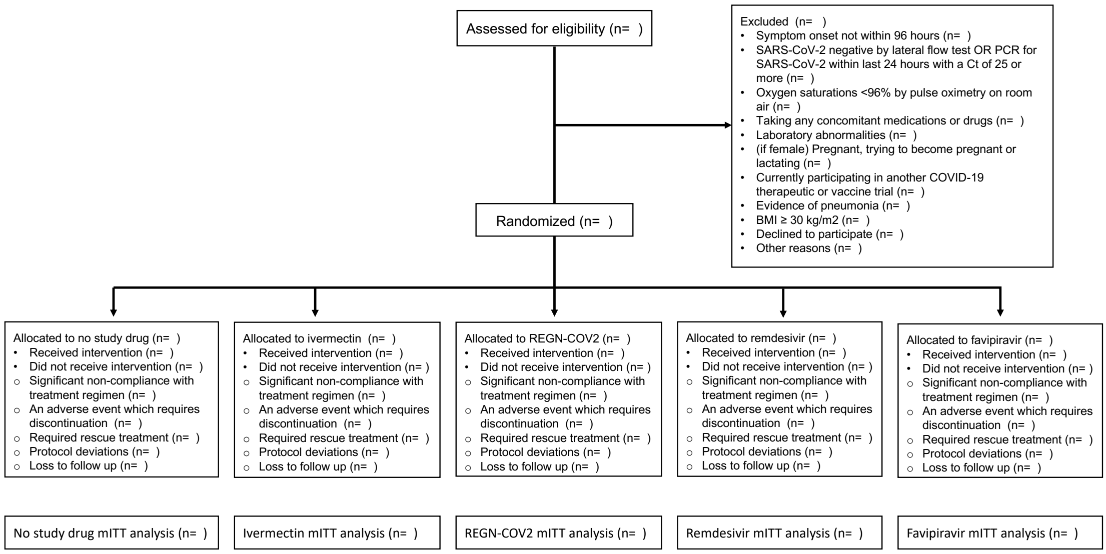

Figure 2: Example CONSORT diagram summarising screening, reasons for excluding patients and randomisation.

- Symptoms of COVID-19 (including fever, or history of fever) for less than 4 days (96 hours);
- Oxygen saturation ≥96% measured by pulse-oximetry at time of screening.

6.3 Withdrawal and follow-up

Level of withdrawal will be tabulated and cover the following aspects:

- Discontinuation from any of the study interventions
- Withdrawal from study follow-up
- Withdrawal from entire study and requests that data is not used.

Data will be tabulated for the timing of withdrawal from follow up or lost to follow up. The number of withdrawals and reason will be recorded. A participant may withdraw from the intervention, or be withdrawn for the following reason.

- Withdrawal of consent by participant
- Alteration of participants circumstances or condition which gives justification for discontinuation as decided by investigator.

A table will summarise the loss to follow up and withdrawals during the study with the corresponding reasons.

6.4 Baseline patient characteristics

The following key baseline characteristics will be summarised, stratified by site:

- Age and sex;
- Baseline viral load (in copies per mL: the mean value of the 4 swabs taken at randomisation);
- Baseline quantitative serum antibody titres (when available);
- Vaccination status (vaccine type, number of doses: expressed as not vaccinated, partially vaccinated and fully vaccinated);

- Days since onset of symptoms.

These will be summarised by their mean or median and standard deviation/range for the continuous variables and by their mean for the binary variables.

7 Analysis

7.1 Outcome definitions

The primary outcome is the inferred rate of viral clearance expressed as a slope coefficient (the mean posterior estimate and the 95% credible interval) for the daily change in the log viral load (on the $\log_{10}$ copies per mL scale). This will be inferred under a Bayesian mixed effects (hierarchical) model using the $\log_{10}$ viral load measurements up until day 7. The observed CT values will be transformed to copies per mL using the set of control samples from each batch.

7.1.1 Viral load quantification

All viral load quantification will be done using ThermoFisher TaqCheck™ SARS-CoV-2 Fast PCR assay. Each PCR assay is run on a 96 well plate, composed of all the aliquots from the transport media of the swabs from 4 patients (10 timepoints each done in duplicate, i.e. 20 samples per patient) and one control set composed of 6 spiked control samples of known viral densities over the range 10^7 to 10^2 viral copies per mL, each done in duplicate (12 in total). For each plate (i.e. batch) we can thus estimate a standard curve (which defines the conversion from the CT value to a number of viral copies per mL). At each interim analysis we will perform a quality control check by comparing the estimated standard curves from each plate, both within sites and across sites (each country will run the PCR assays separately). In addition, we will use the standard curve data to estimate heteroscedasticity in the viral load estimation. We expect the PCR measurement error to increase as the viral load decreases. Heteroscedasticity can be shown visually by plotting the true viral load densities in the control duplicated samples against their observed difference in CT value. We can then estimate the variance in the differences as a function of the true viral density.

To help adjust for variation in the human cell content of the swabbed sample the qPCR method assesses viral densities and also human cell densities using RNaseP as a human cell marker.

7.2 Analysis Methods

7.2.1 Statistical model for the analysis of the serial viral load data

The primary analysis will consist of fitting Bayesian hierarchical (mixed effects) linear models to the $\log_{10}$ viral load data up until day 7. The treatment effect is defined as the proportional change (expressed as a multiplicative term) in the population slope of the daily change in log viral load. We will model the data on the copies per mL scale, after conversion from CT values using controls from each 96 well plate. The standard curve transformation will be done by fitting a linear mixed effects (random slope and random intercept for each plate) model to the control data: regressing the CT values on the known log viral densities. This borrows information across plates but allows for batch effects.

The general model likelihood takes the following form:

$$y_{i,t,k} \sim \text{Student}\left(\lambda, \alpha_0 + \alpha_i + \alpha_k + \alpha_{cov} + \gamma x_{i,t} + \beta_0 e^{\beta_k + \beta_i + \beta_{cov} + \beta_{T(i)}} t, \sigma^2\right)$$

where:

- $y_{i,t,k}$ is the log viral load ($\log_{10}$ copies per mL) for patient i at time t (in days since randomisation) from site k.
- $T(i)$ is the randomized treatment allocation for individual i.
- σ^2 is the variance of the measurement error.
- λ is the degrees of freedom of the student-t error model.

- The slope of viral clearance decomposes into 5 multiplicative terms: the population mean slope β_0; the site random effect β_k (e^{β_k} is thus the proportional change in slope); the individual random effect β_i; the sum of the covariate effects β_{cov}; and the treatment effect (fixed across sites and individuals) $\beta_{T(i)}$. All comparisons are made relative to the no antiviral treatment control arm, so we set $\beta_{control} = 0$.

- The intercept term (baseline log viral load) decomposes into 4 terms: the population intercept α_0; the site random effect α_k; the individual random effect α_i; and the sum of any covariate effects α_{cov}.

- $x_{i,t}$ is the relative human RNaseP quantification (RNaseP ΔCT value: this is proportional to the log number of human cells) for patient i at time t. The parameter γ thus provides an adjustment for human cell content in each swab. We scale $x_{i,t}$ so that it has mean 0.

The analysis will three model types. The primary model is the linear model with adjustment for virus variant and site. A secondary model then incorporates the covariate adjustment terms α_{cov} and β_{cov} (see below for their definition). If analysis only has data from one site, then the site random effect term α_k will be dropped. The final model (model 3) is a non-linear version of models 1 and 2 (see below). Efficacy estimation is made based on model 2 (log-linear model with covariate adjustment).

We have chosen a student-t distribution for the model likelihood (with the number of degrees of freedom λ estimated from the data) as this is robust against departures from normal (Gaussian) error, and against model mis-specification, notably concerning the assumption of log-linear decline in viral loads. Prior analyses carried out in order to prepare this statistical analysis plan suggest that a considerable number (e.g. up to 5%) of viral load densities can depart substantially from the expected distribution under a simple log-linear model with Gaussian error [6]. This can partially be explained by patterns of bi-exponential decay in viral loads. It is unclear if the slope of the second phase of elimination in a bi-exponential decline is affected by interventions. We therefore choose not to fit bi-exponential models as this makes the interpretation of the treatment effect more difficult and prior modelling suggested no major impact in terms of treatment effect estimation [6].

Goodness of fits will be assessed using leave-one-out validation [12] and Bayesian R^2 approximation [13].

7.2.2 Covariate adjustment

In addition to the human RNaseP adjustment, the primary analyses will adjust for the following covariates (through the parameters $\alpha_{cov}, \beta_{cov}$):

- Age (in years - scaled to have mean zero);

- A quantitative measure of serological status using a pre-specified antibody (not yet decided). These data are not yet available.

- Previous SARS-CoV-2 vaccination (as an ordinal variable: 0: no doses; 1: partially vaccinated with only one dose: 2: fully vaccinated with 2 doses; 3: 3 or more doses);

- The SARS-CoV-2 lineage or sub-lineage. The reference strain is the Delta lineage, with Omicron sub-divided into descendant lineages (BA.1, BA.2 etc). Given the difficulty in prespecifying exact subgroupings, we allow some flexibility (e.g. considering BA.2.75 as separate from previous BA.2 lineages).

- The trial "epoch" (only for analyses including non fully overlapping intervention arms): this is defined as the discrete intervals of time at which new interventions enter or exit the study. This is to adjust for temporal effects which could confound across arm comparisons made for non-contemporaneously recruiting arms.

If any of the binary covariates show no variation (e.g. all patients are vaccinated) then we will drop the term from the model. We will fit both the covariate adjusted model and the non-adjusted model in all instances as to check for sensitivity to the covariate model. A sensitivity analysis regarding temporal drift will be done by analysing each arm individually along with contemporaneously enrolled controls.

7.2.3 Resolution of fever and symptoms

Time to resolution of fever and time to resolution of symptoms will be analysed by comparing Kaplan-Meier curves (right censored data). Resolution of fever is defined as an axillary temperature $\leq$37.0C for both measurements taken over one day (at least 24 hours). Resolution of symptoms is defined as no reported symptoms. An additional analysis will look at the proportion of patients with lymphocyte count within the normal ranges at D3 and D7. Differences in time to resolution for both endpoints will be tested using the log-rank test. Differences will be quantified by the change in the median time to resolution.

7.3 Pharmacokinetic analyses

All patients will have 4 plasma samples taken at: randomisation, day 3, day 7 (all three are pre-dose), and finally day 14. Drug concentrations will be quantitated from these samples in order to explore dose-response relationships between drug (or metabolite) exposure and outcome (slope of viral clearance). There are two objectives for the pharmacokinetic data:

- Firstly, to verify that plasma concentrations are within the expected range (using published pharmacokinetic data from equivalent patient groups and dosing);
- Secondly, to explore whether there is any correlation between plasma concentrations and outcome (rate of viral clearance).

The first objective will use published pharmacokinetic data to assess whether drug levels are within the expected range. For the second objective, the primary analysis will be performed using the day 3 level (a trough level). We will fit a linear model between the day 3 level (log transformed) and the mean viral clearance slope estimate from the main analysis in patients allocated to the intervention arm. Significance is defined as a two-sided test at the 5% level.

7.4 Subgroup analyses

The primary pre-specified subgroup analysis will look at the relationship between the efficacy of the monoclonal antibodies (previously the Regeneron antibody cocktail casirivimab/imdevimab [8], currently tixagevimab/cilgavimab) and the virus genotype. Previously we had pre-specified subgroups based on the major WHO lineages, however, given the recent trends in SARS-CoV-2 evolution, this approach does not make much sense anymore. Instead we will use *in vitro* assay data to define a set of canonical non-synonymous amino-acid changes which are predicted to substantially change the efficacy of the monoclonal in question. For each monoclonal in the platform, we will write a specific appendix outlining exact subgroup definitions (amino acid calls are based on the nanopore sequencing data).

Subgroup analyses will be done by fitting an interaction term $\delta x_{var}(i) e^{\beta_{T(i)}}$ for the relevant $T(i)$; where $x_{var}(i)$ is the virus genotype defined subgroup infecting individual i.

Secondary subgroup analyses will be carried out only for treatment arms that show an antiviral effect (meet the success criteria). We will look at subgroup effects for (listed in order of priority):

- SARS-CoV-2 variant of concern;
- Serological status on admission (using quantitative serum antibody);
- Vaccination status;
- Age and sex;

For the analysis of tixagevimab/cilgavimab, see Supplementary section **??**.

7.5 Prior distributions

In all the following, for the normal distributions, the second term corresponds to the scale (standard deviation not the variance). The viral load values are on the $\log_{10}$ scale (i.e. for the intercept term 6 would correspond to 1,000,000 copies per ml).

Population level parameters

$$\alpha_0 \sim \text{Normal}(6, 2) \quad \text{(population intercept)}$$

$$\beta_0 \sim \text{Normal}(-0.5, 1) \quad \text{(population slope)}$$

$$\sigma \sim \text{Normal}(1.5, 3) \quad \text{(standard deviation of measurement error)}$$

$$\gamma \sim \text{Normal}(0, 1) \quad \text{(human RNaseP adjustment)}$$

$$\beta_{T(i)} \sim \text{Normal}(0, 0.5) \quad \text{log treatment effect (subgroups also)}$$

Covariate effects

$$\beta_{cov} \sim \text{Normal}(0, 1) \quad \text{(covariate effect on slope)}$$

$$\alpha_{cov} \sim \text{Normal}(0, 1) \quad \text{(covariate effect on intercept)}$$

Random effects and hyperparameters

$$\Omega \sim \text{Cholesky}(2) \quad \text{(correlation matrix for individual random effects)}$$

$$\Sigma \sim \text{Exponential}(1) \quad \text{(standard deviation for individual random effects)}$$

$$\lambda \sim \text{Exponential}(1) \quad \text{(t-distribution degrees of freedom)}$$

7.6 Sensitivity analyses

Sensitivity to the prior specification will be assessed by multiplying all prior standard deviation terms by 5. Sensitivity to the assumption of log-linear decline will be assessed by fitting an 'up-down' model of viral dynamics [14] (where "up" refers to viral multiplication and "down" the subsequent clearance). Under this model the likelihood is (dropping batch, site, and covariate terms for notational simplicity, these are added analogous to the previous log-linear model) given by:

$$y_{i,t} \sim \text{Student}\left(\lambda, \alpha_0 + \alpha_i + \log\left\{\frac{\beta_1^i + \beta_2^i}{\beta_2^i e^{-\beta_1^i[t - t_{\max}^i]} + \beta_1^i e^{\beta_2^i[t - t_{\max}^i]}}\right\}, \sigma^2\right)$$

Under this model, the term $\alpha_0 + \alpha_i$ is the individual peak log viral load which occurs at time $t_{\max}^i$ (this is the time relative to randomisation, if negative it is therefore unobserved). The parameters β_1^1, β_2^i are the growth and clearance rates for individual i, respectively.

If some patients are enrolled very early in the course of their illness, it is possible that their viral loads increase over the first days of follow-up. This would mean that the clearance rate estimated under a simple log-linear decline model would be biased towards a shallower slope. Randomisation protects against confounding bias as the sample size increases, however for small sample sizes unequal allocation of 'pre-peak' versus 'post-peak' individuals could influence treatment effect estimates. Treatment effects are inferred in the same way as for the log-linear model (proportional change in the population slope coefficients), by acting on β_2 (note that in reality, the treatment effect most likely would change all three parameters $\alpha_0, \beta_1, \beta_2$, however this would mean inferring three separate treatment effects as the model is non-mechanistic).

7.7 Missing data

Missing viral load data will not be imputed. The model will be fit to all available viral load densities up until day 7 or day of treatment deviation. For all analyses, missing data on the key baseline variables (vaccination, virus genotype, antibody rapid test) will be imputed at random using the observed site-specific covariate distribution multiple times (5 times, the model will be refit). We do not expect much missing data (eg <1%). If delays in genotyping result in missing variant data, we will impute the variant data using genetic surveillance data.

7.8 Harms

Safety analyses will include all patients who have received at least one dose of the intervention. The safety and tolerability data will be pooled from all the sites that receive the same study intervention. The safety and tolerability will be assessed by comparing the frequency of adverse events and serious adverse events when compared to the control intervention.

All adverse event summaries will refer to treatment emergent adverse events, i.e. adverse events that newly started or increased in intensity after study drug administration (or from hour 0 in the control group receiving no intervention). Adverse events will be graded according to CTCAE V5.0. Adverse Events (AEs) of grade 3 and above will be recorded, the grading is as follows - 1 = mild, 2 = moderate, 3 = severe, 4 = life-threatening, 5 = fatal. All Serious Adverse Events (SAEs) will be recorded, a serious adverse event was any untoward medical occurrence that resulted in death, was life threatening, required inpatient hospitalization or prolongation of existing hospitalization, resulted in persistent or significant disability/incapacity or consisted of a congenital anomaly or birth defect.

We will tabulate AE of grade 3 and higher, SAEs, and deaths, by treatment arm with comparisons made relative to the no study drug arm.

None of the drugs evaluated will be pre-registration or pre-approval, so adverse effect profiles for each are already well known.

7.9 Statistical software and analysis implementation

All analyses will be be in done in R. Interim analyses and the final analyses will be done using RMarkdown scripts, pre-coded to ensure full transparency. All code for the statistical analysis is available on the following github repository: `https://github.com/jwatowatson/PLATCOV-SAP`. This ensures full version control by tracking all changes made to the analysis code.

The statistical models for the analysis of the serial viral load data are written in *stan* and fitted via the *rstan* interface [15]. These are available on the github repository. For each model, we will run 6 parallel chains for 10,000 iterations, discarding half for burn-in and thinning every 40 (thus 1,000 posterior draws).

Convergence of Bayesian fits will be assessed visually by examining the traceplots of the key model parameters and assessing the Rhat values for each parameter (Gelman-Rubin statistic).

References

[1] Weinreich DM, Sivapalasingam S, Norton T, Ali S, Gao H, Bhore R, et al. REGEN-COV Antibody Combination and Outcomes in Outpatients with Covid-19. New England Journal of Medicine. 2021;385(23):e81. doi:10.1056/NEJMoa2108163.

[2] Gottlieb RL, Vaca CE, Paredes R, Mera J, Webb BJ, Perez G, et al. Early Remdesivir to Prevent Progression to Severe Covid-19 in Outpatients. New England Journal of Medicine. 2022;386(4):305–315. doi:10.1056/NEJMoa2116846.

[3] Jayk Bernal A, Gomes da Silva MM, Musungaie DB, Kovalchuk E, Gonzalez A, Delos Reyes V, et al. Molnupiravir for oral treatment of Covid-19 in nonhospitalized patients. New England Journal of Medicine. 2021;.

[4] Hammond J, Leister-Tebbe H, Gardner A, Abreu P, Bao W, Wisemandle W, et al. Oral Nirmatrelvir for High-Risk, Nonhospitalized Adults with Covid-19. New England Journal of Medicine. 0;0(0):null. doi:10.1056/NEJMoa2118542.

[5] Butler CC, Hobbs FDR, Gbinigie OA, Rahman NM, Hayward G, Richards DB, et al. Molnupiravir plus usual care versus usual care alone as early treatment for adults with COVID-19 at increased risk of adverse outcomes (PANORAMIC): an open-label, platform-adaptive randomised controlled trial. The Lancet. 2023;doi:10.1016/S0140-6736(22)02597-1.

[6] Watson JA, Kissler SM, Day NPJ, Grad YH, White NJ. Characterizing SARS-CoV-2 Viral Clearance Kinetics to Improve the Design of Antiviral Pharmacometric Studies. Antimicrobial Agents and Chemotherapy. 2022;66(7):e00192–22. doi:10.1128/aac.00192-22.

[7] Schilling WH, Jittamala P, Watson JA, Ekkapongpisit M, Siripoon T, Ngamprasertchai T, et al. Pharmacometric assessment of the in vivo antiviral activity of ivermectin in early symptomatic COVID-19. medRxiv. 2022;doi:10.1101/2022.07.15.22277570.

[8] Jittamala P, Schilling WH, Watson JA, Luvira V, Siripoon T, Ngamprasertchai T, et al. Clinical antiviral efficacy of remdesivir and casirivimab/imdevimab against the SARS-CoV-2 Delta and Omicron variants. medRxiv. 2022;doi:10.1101/2022.10.17.22281161.

[9] Gamble C, Krishan A, Stocken D, Lewis S, Juszczak E, Doré C, et al. Guidelines for the Content of Statistical Analysis Plans in Clinical Trials. JAMA. 2017;318(23):2337–2343. doi:10.1001/jama.2017.18556.

[10] Kissler SM, Fauver JR, Mack C, Olesen SW, Tai C, Shiue KY, et al. Viral dynamics of acute SARS-CoV-2 infection and applications to diagnostic and public health strategies. PLoS biology. 2021;19(7):e3001333.

[11] Weinreich DM, Sivapalasingam S, Norton T, Ali S, Gao H, Bhore R, et al. REGN-COV2, a Neutralizing Antibody Cocktail, in Outpatients with COVID-19. New England Journal of Medicine. 2020;doi:10.1056/NEJMoa2035002.

[12] Vehtari A, Gelman A, Gabry J. Practical Bayesian model evaluation using leave-one-out cross-validation and WAIC. Statistics and computing. 2017;27(5):1413–1432.

[13] Gelman A, Goodrich B, Gabry J, Vehtari A. R-squared for Bayesian Regression Models. The American Statistician. 2019;73(3):307–309. doi:10.1080/00031305.2018.1549100.

[14] Singanayagam A, Hakki S, Dunning J, Madon KJ, Crone MA, Koycheva A, et al. Community transmission and viral load kinetics of the SARS-CoV-2 delta (B. 1.617. 2) variant in vaccinated and unvaccinated individuals in the UK: a prospective, longitudinal, cohort study. The Lancet Infectious Diseases. 2021;.

[15] Stan Development Team. RStan: the R interface to Stan; 2020. Available from: http://mc-stan.org/.

S1 Updated sample size simulations

S2 Casirivimab/imdevimab

The casirivivab/imdevimab hypothesised subgroup is defined as: (i) G to S amino acid change in the 446 residue OR (ii) any amino acid change in the 444 residue .

S3 Tixagevimab/cilgavimab

The tixagevimab/cilgavimab hypothesised resistant subgroup is defined by: (i) any amino-acid change from wild-type in the 486 residue of the spike protein AND (ii) an amino-acid change in the 346 residue OR the 444 residue.

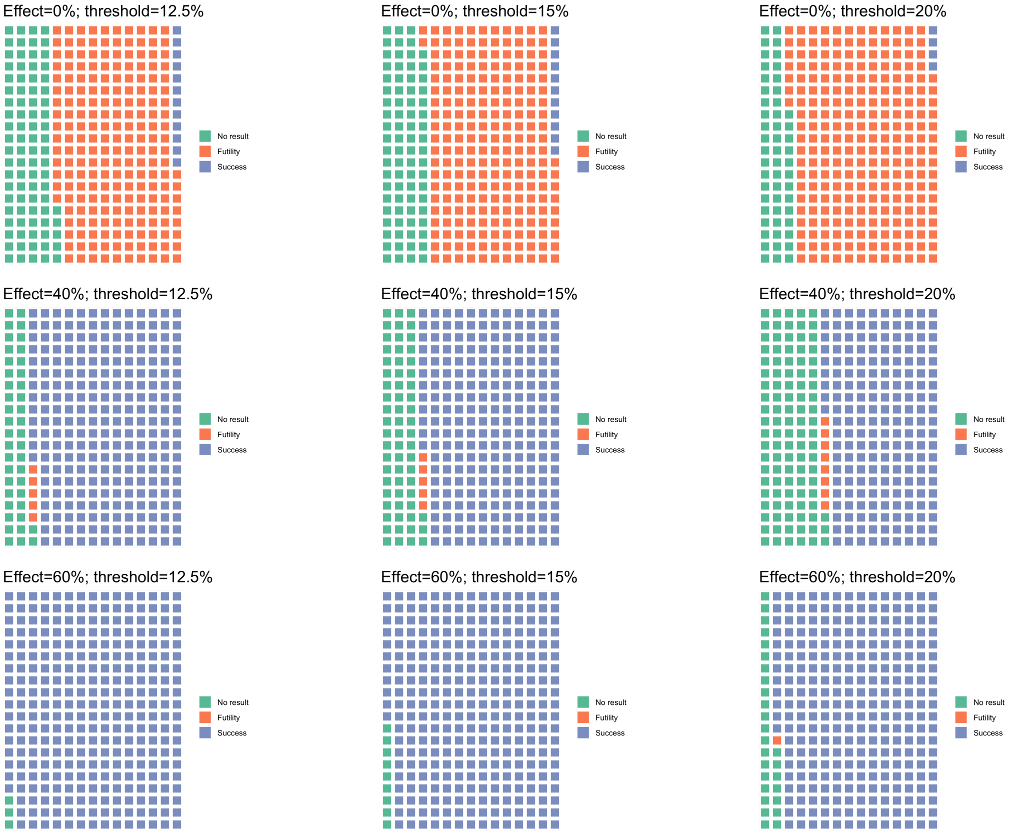

Figure S1: Waffle plots showing the proportion of each outcome (success: blue; futility: orange; no result by 120 patients: green) for the success versus futility stopping rule. 100 simulations were run for each permutation of the effect size and λ_1 threshold.

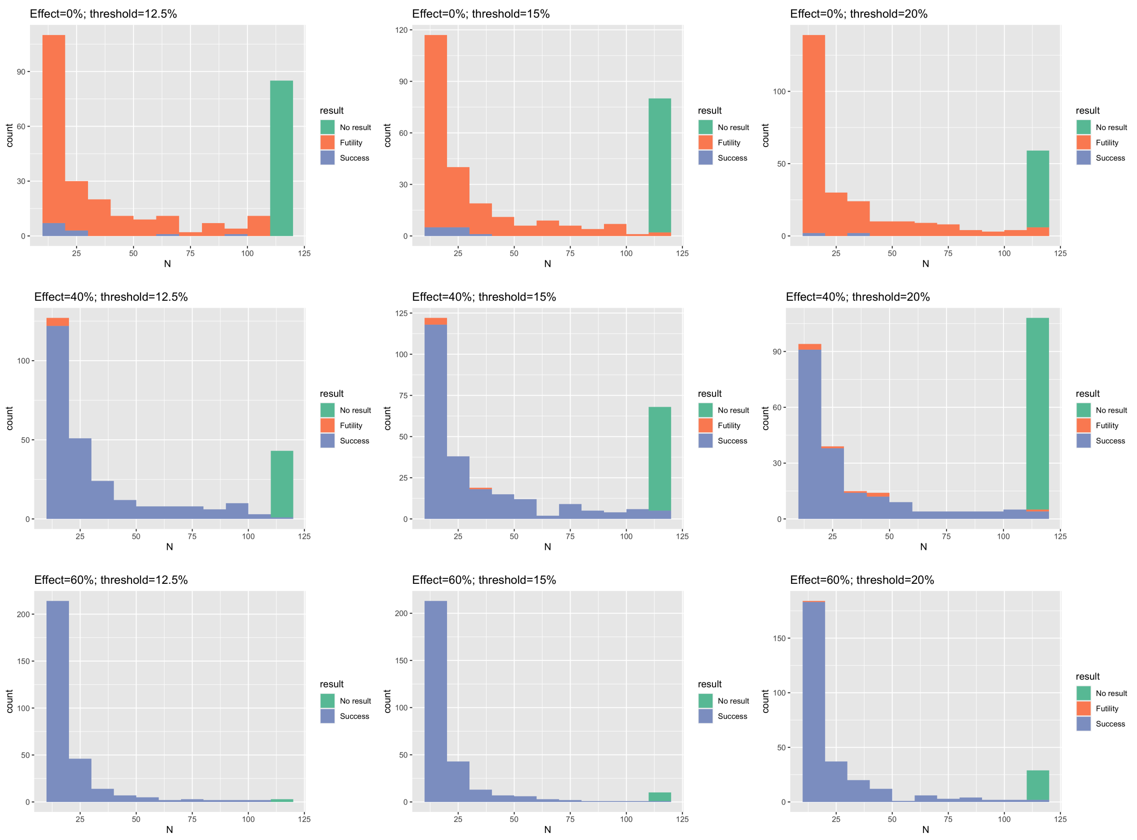

Figure S2: Histogram of sample size until stopping rule is met, same colors as in Figure S1.

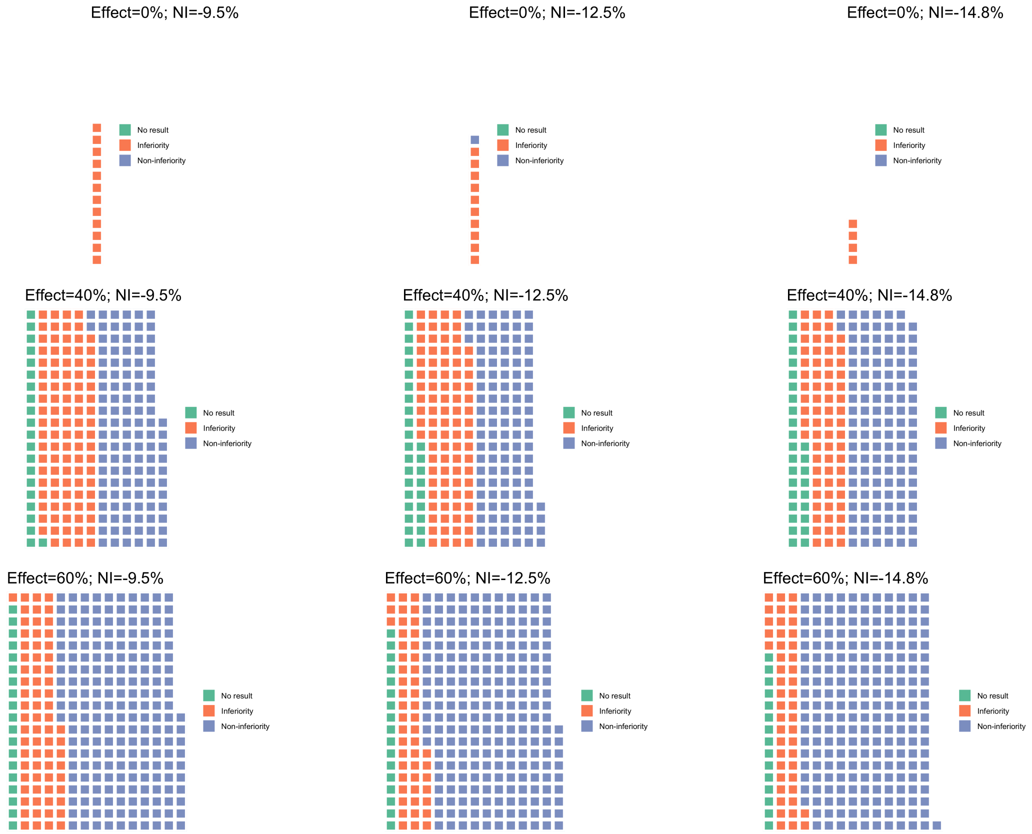

Figure S3: Waffle plots showing the proportion of each outcome (non-inferiority: blue; inferiority: orange; no result by 120 patients: green) for the non-inferiority stopping rule. 100 simulations were run for each permutation of the effect size and λ_2 threshold.

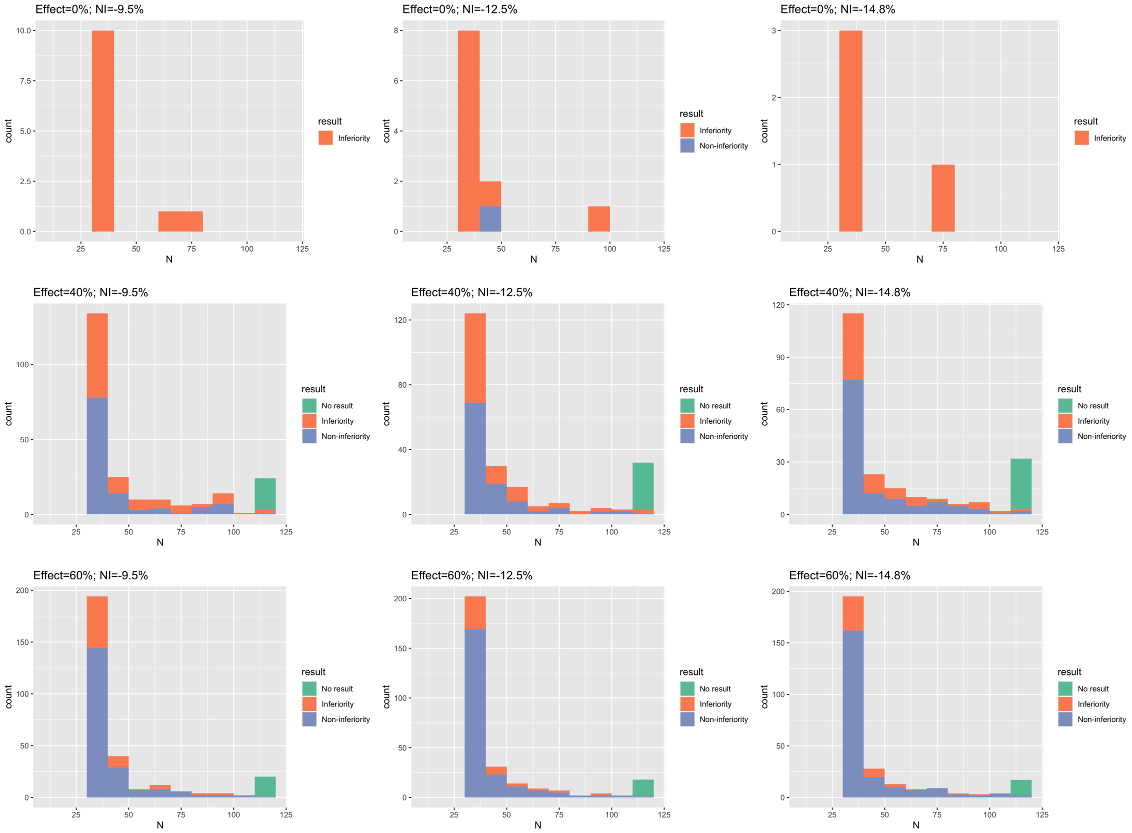

Figure S4: Histogram of sample size until stopping rule is met, same colors as in Figure S3.
